# Supplementary material for: Automated sequence design of 2D wireframe DNA origami with honeycomb edges
Source: Nat Commun. 2019 Nov 28;10:5419. doi: 10.1038/s41467-019-13457-y (PMC6882874; doi:10.1038/s41467-019-13457-y)
Supplement: Supplementary file 1 — Supplementary Information [file 41467_2019_13457_MOESM1_ESM.pdf]

Supplementary Information

Automated Sequence Design of 2D Wireframe DNA origami with Honeycomb Edges

Jun *et al.*

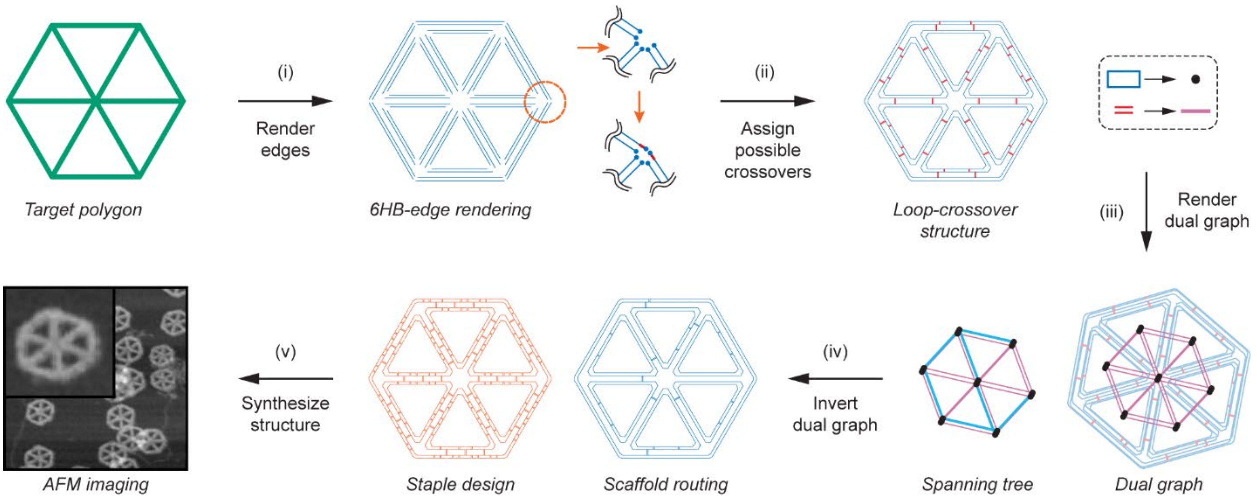

Supplementary Figure 1 Schematic illustrating design algorithm for the 6HB-based hexagonal DNA origami. The arbitrary target geometry is based on a polygon mesh, with discretized line segments (step i) to represent six DNA duplexes per wireframe edge with the endpoints joined (step ii) to form closed loops with geometrically allowable scaffold double crossovers between them. The dual graph of the loop-crossover structure is obtained (step iii) by converting each closed scaffold loop to a node and each possible scaffold double crossover connecting them to an edge. The minimum spanning tree of the dual graph was then determined and inverted (step iv), defining the DNA scaffold routing. Staple sequences generated (step v) by the algorithm were used with the input scaffold to synthesize the 2D DNA origami in one-pot thermal annealing.

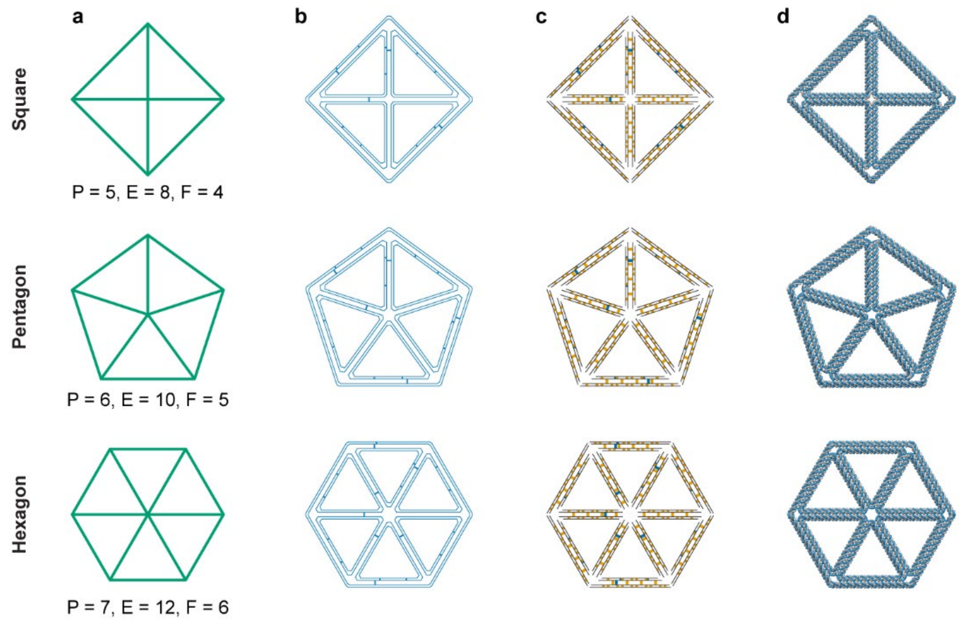

Supplementary Figure 2 Target geometries, scaffold routing paths, crossover models, and atomic models for polygons. **a** The target geometries. The letters, P, E, and F denote the number of points, edges, and faces, respectively. **b** Scaffold routing path. The continuous blue loop is the single-stranded DNA scaffold that routes throughout the entire origami object of arbitrary shape. **c** Scaffold double crossovers (blue) determined by the spanning tree algorithm and staple double crossovers (orange). **d** Atomic model.

Supplementary Table 1 Design parameters for polygons with the DX and 6HB edges. # indicates the type of scaffolds in **Supplementary Table 4**.

Edge type	Target geometry	Edge length	**Scaffold**			**Staples**		
			Required length	# of double crossovers	# of unpaired nucleotides	# of staples	# of double crossovers	# of unpaired nucleotides
**DX**	Square	84-bp	1,756-nt (#2)	4	20	42	32	92
	Pentagon	84-bp	1,986-nt (#2)	5	46	48	35	120
	Hexagon	84-bp	2,185-nt (#2)	6	43	51	42	120
**6HB**	Square	84-bp	5,902-nt (#1)	14	190	133	214	244
	Pentagon	84-bp	6,475-nt (#1)	17	125	154	233	352
	Hexagon	84-bp	7,157-nt (#1)	20	95	168	262	382
	Hexagon	63-bp	5,645-nt (#1)	20	95	123	190	382

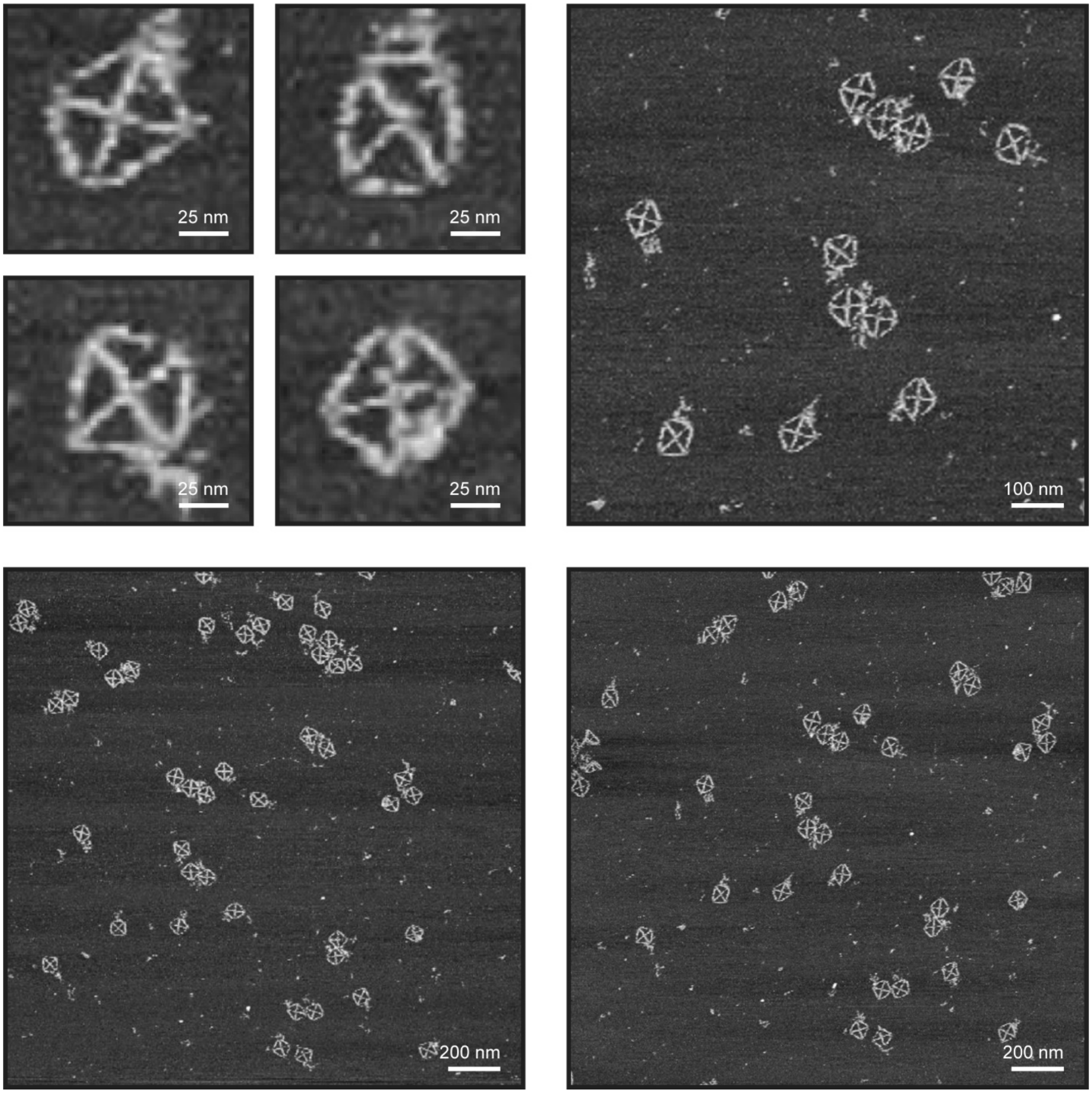

Supplementary Figure 3 AFM imaging of DX-based square DNA origami of 84-bp edge-length.

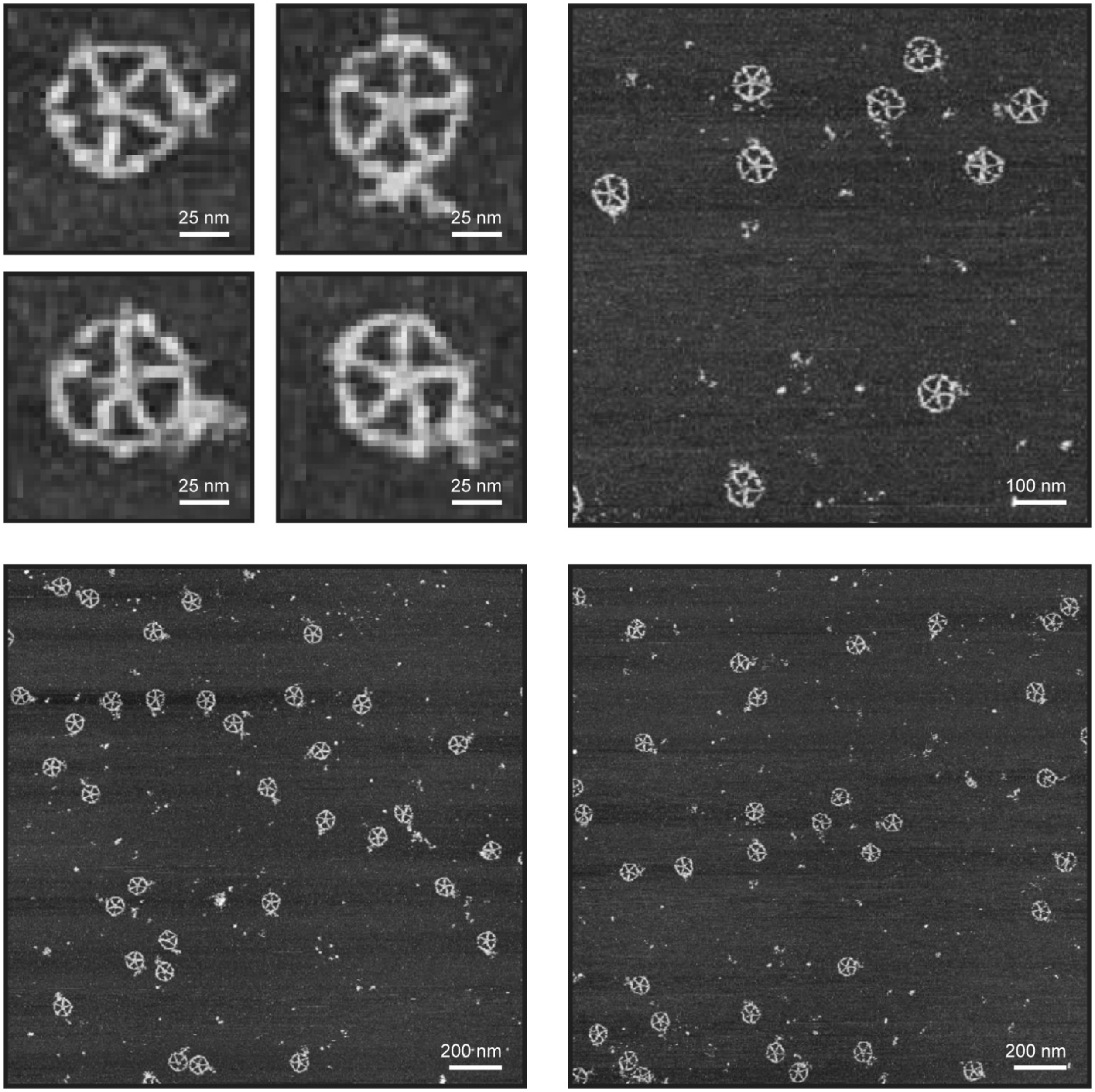

Supplementary Figure 4 AFM imaging of DX-based pentagonal DNA origami of 84-bp edge-length.

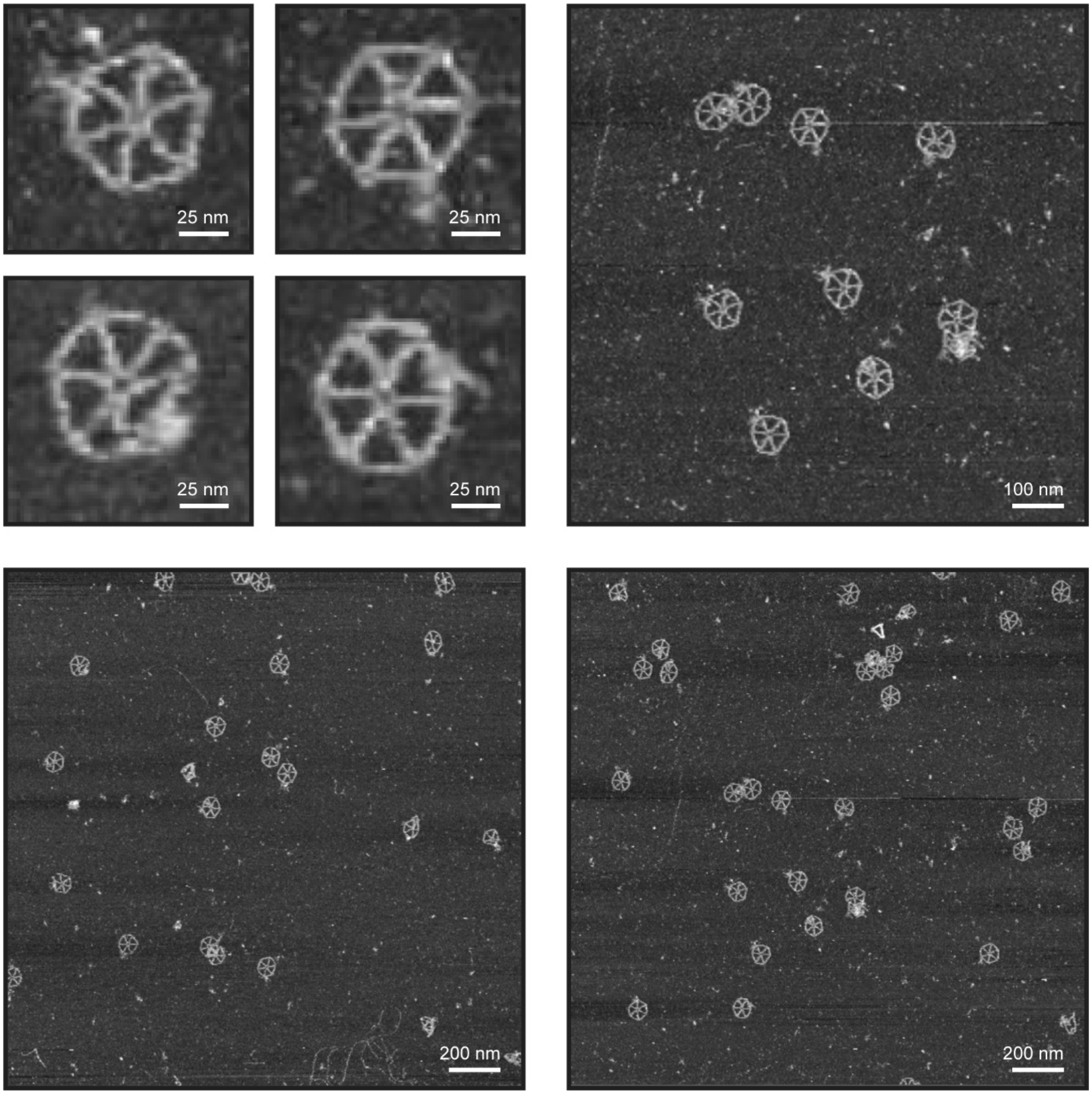

Supplementary Figure 5 AFM imaging of DX-based hexagonal DNA origami of 84-edge-length.

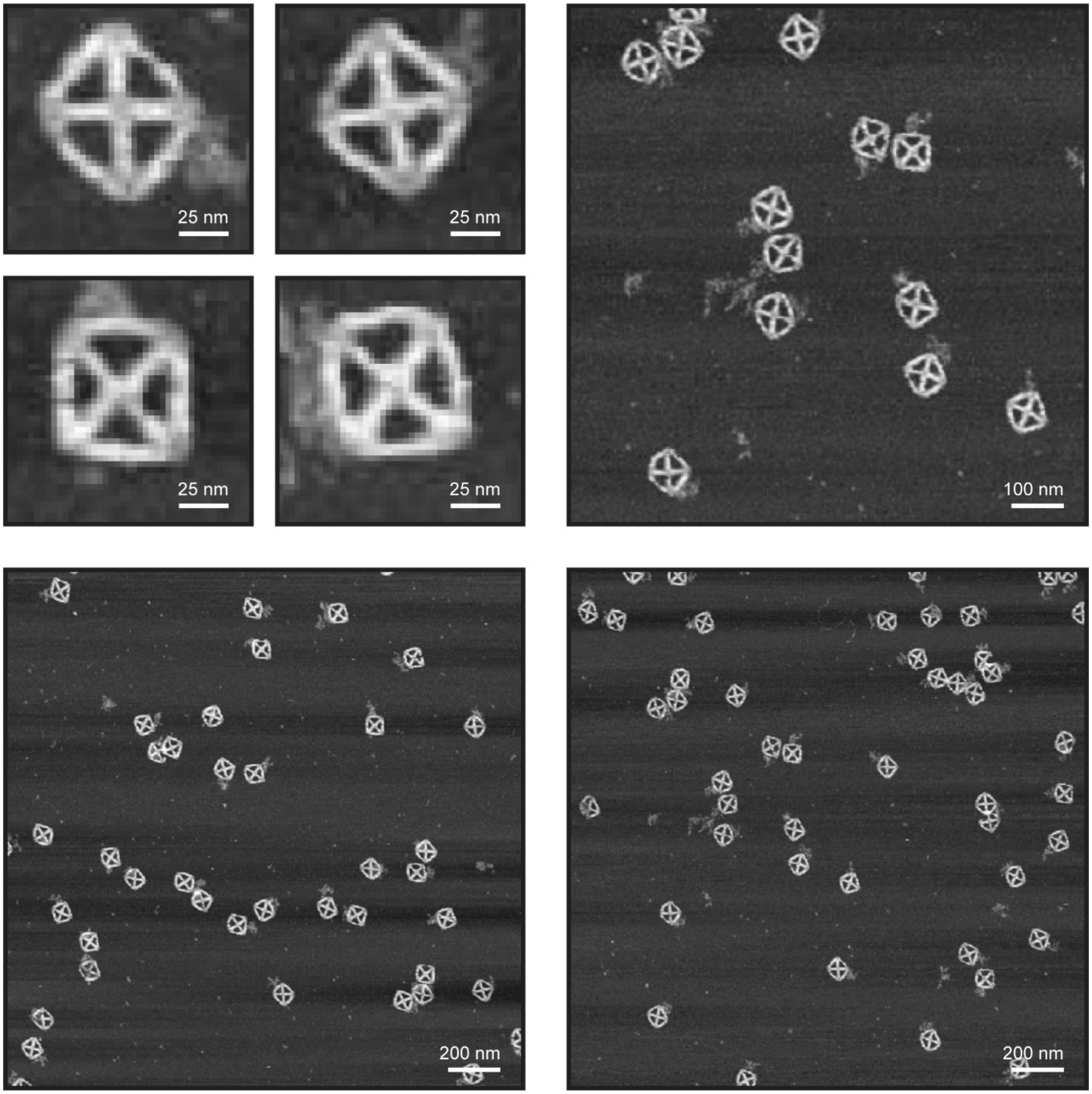

Supplementary Figure 6 AFM imaging of 6HB-based square DNA origami of 84-bp edge-length.

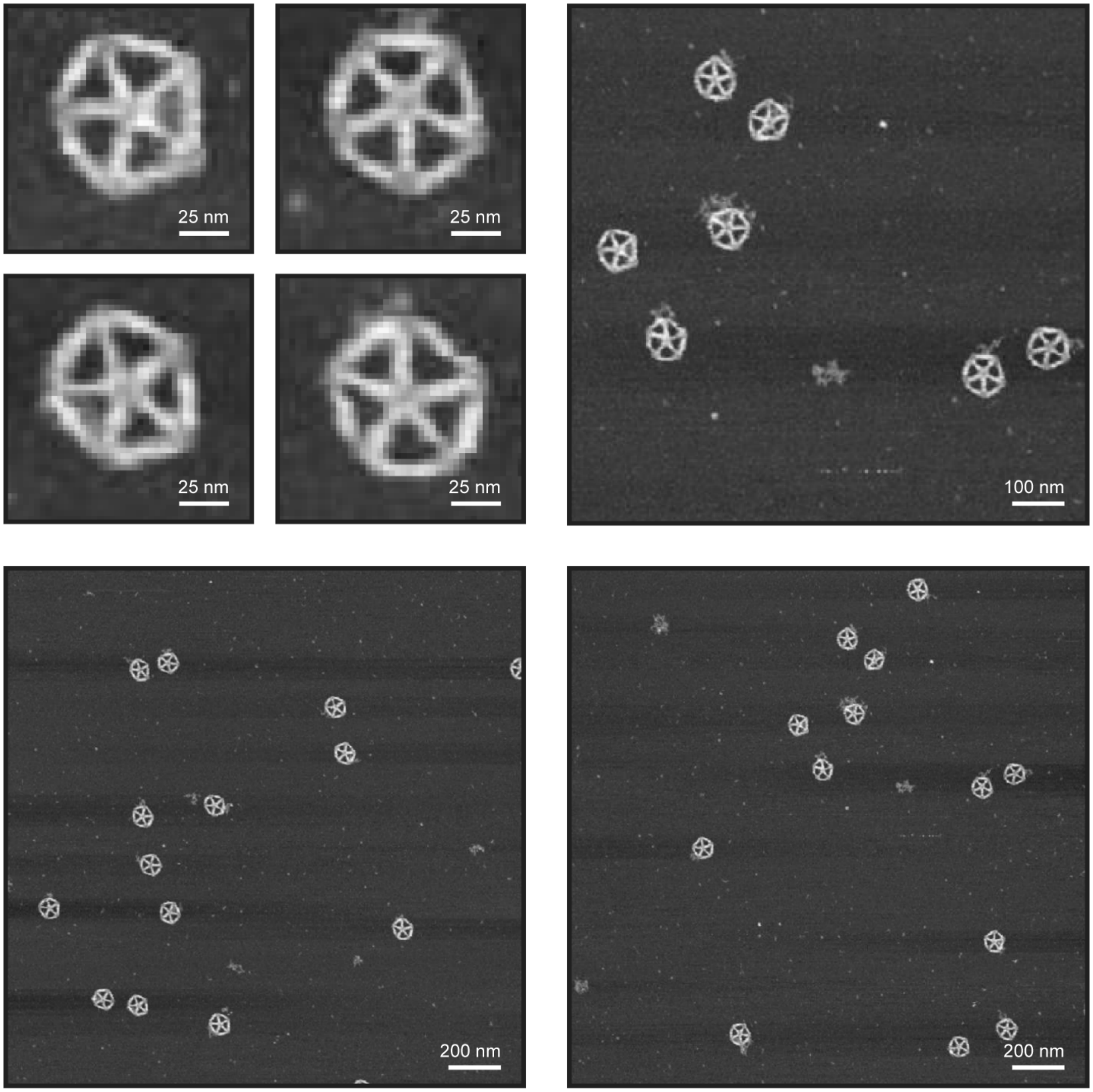

Supplementary Figure 7 AFM imaging of 6HB-based pentagonal DNA origami of 84-bp edge-length.

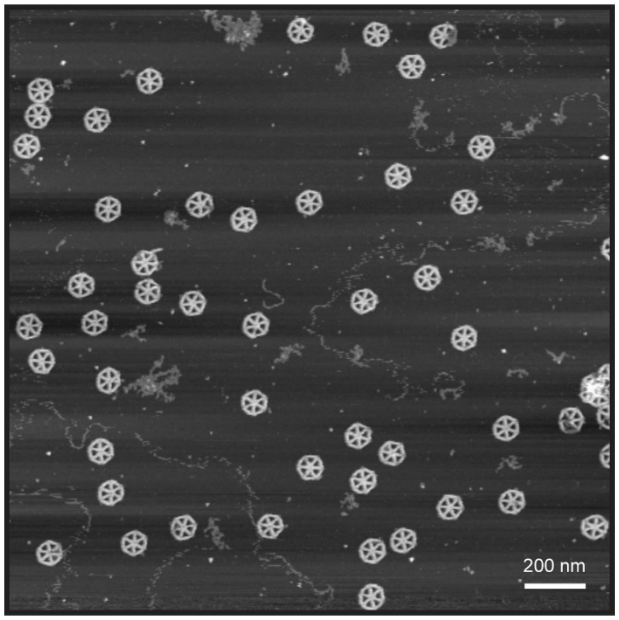

Supplementary Figure 8 AFM imaging of 6HB-based hexagonal DNA origami of 84-edge-length.

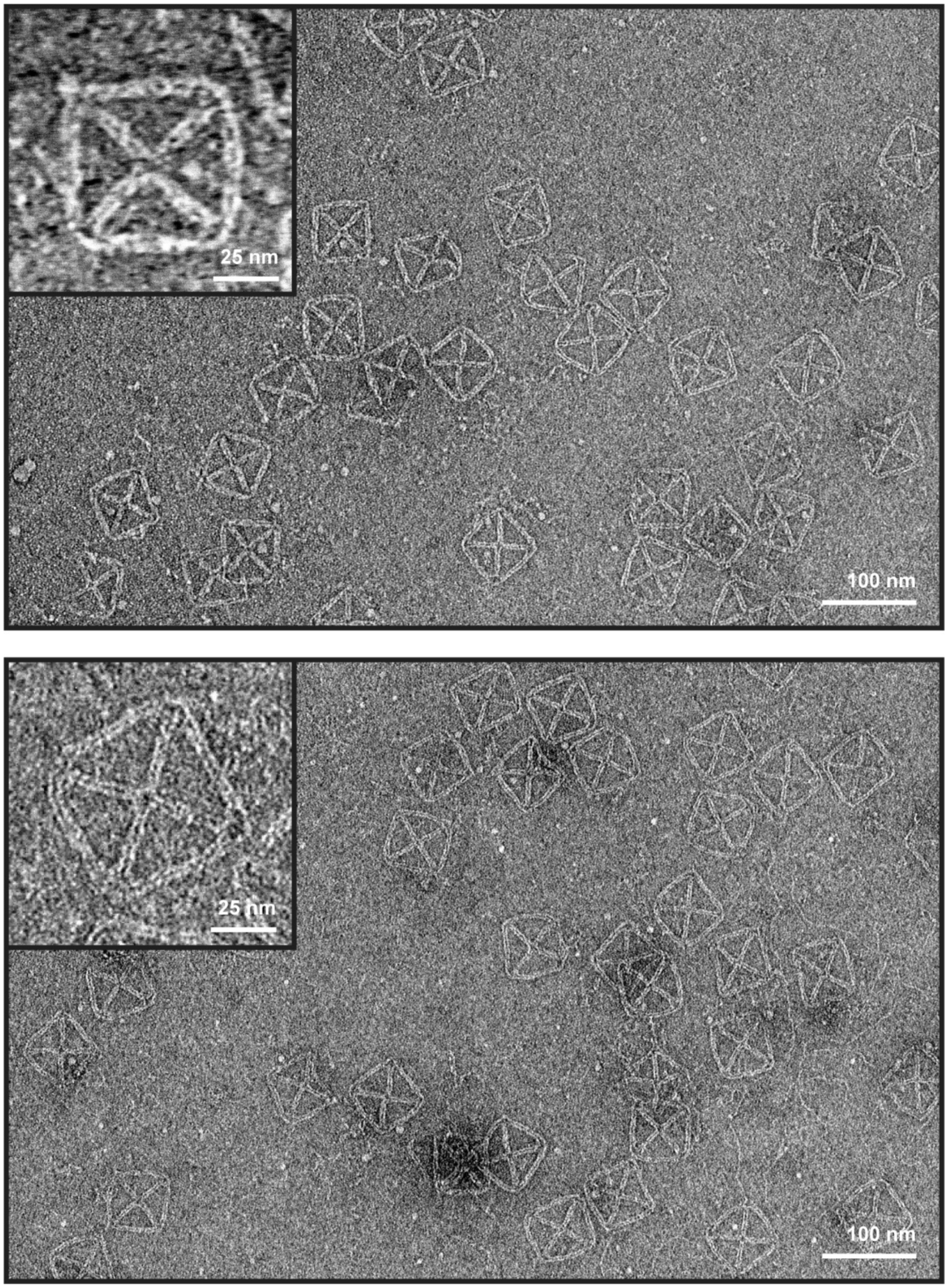

Supplementary Figure 9 TEM imaging of 6HB-based square DNA origami of 84-bp edge-length.

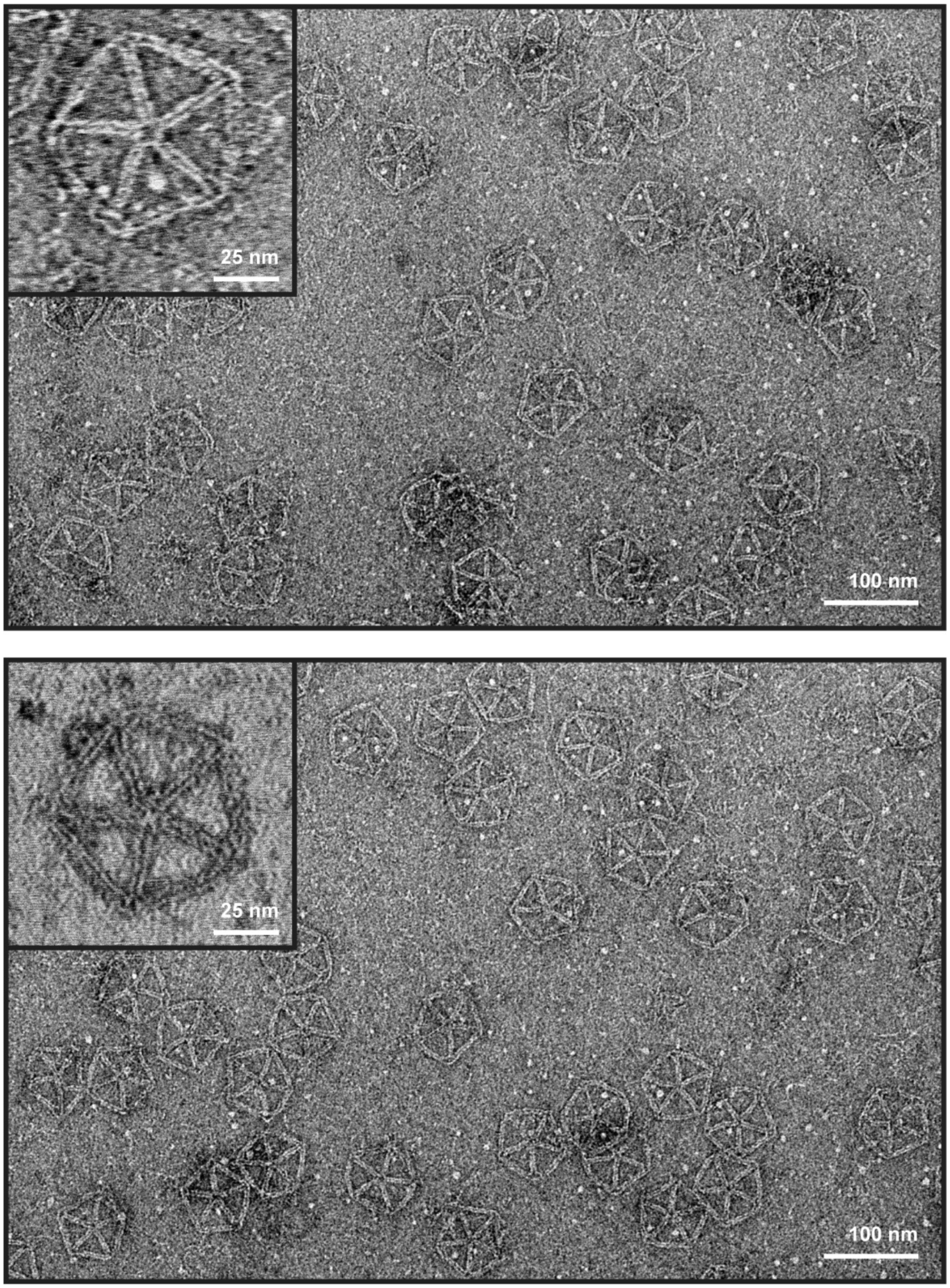

Supplementary Figure 10 TEM imaging of 6HB-based pentagonal DNA origami object of 84-bp edge-length.

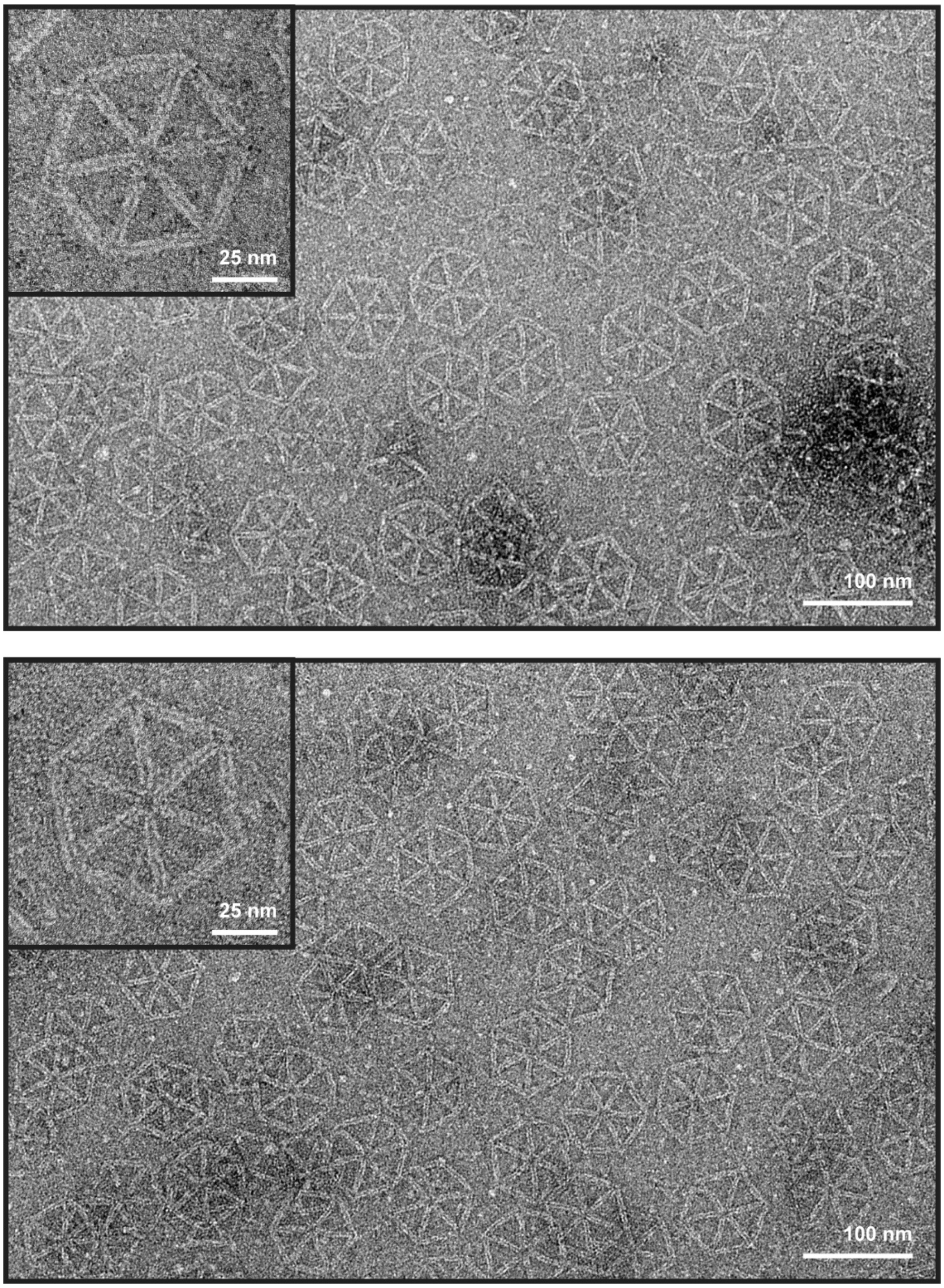

Supplementary Figure 11 TEM imaging of 6HB-based hexagonal DNA origami object of 84-bp edge-length.

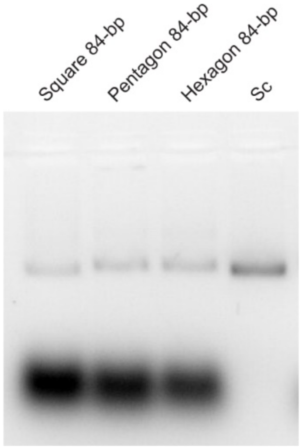

Supplementary Figure 12 Agarose gel electrophoresis for 6HB-based square, pentagonal, and hexagonal DNA origami objects.

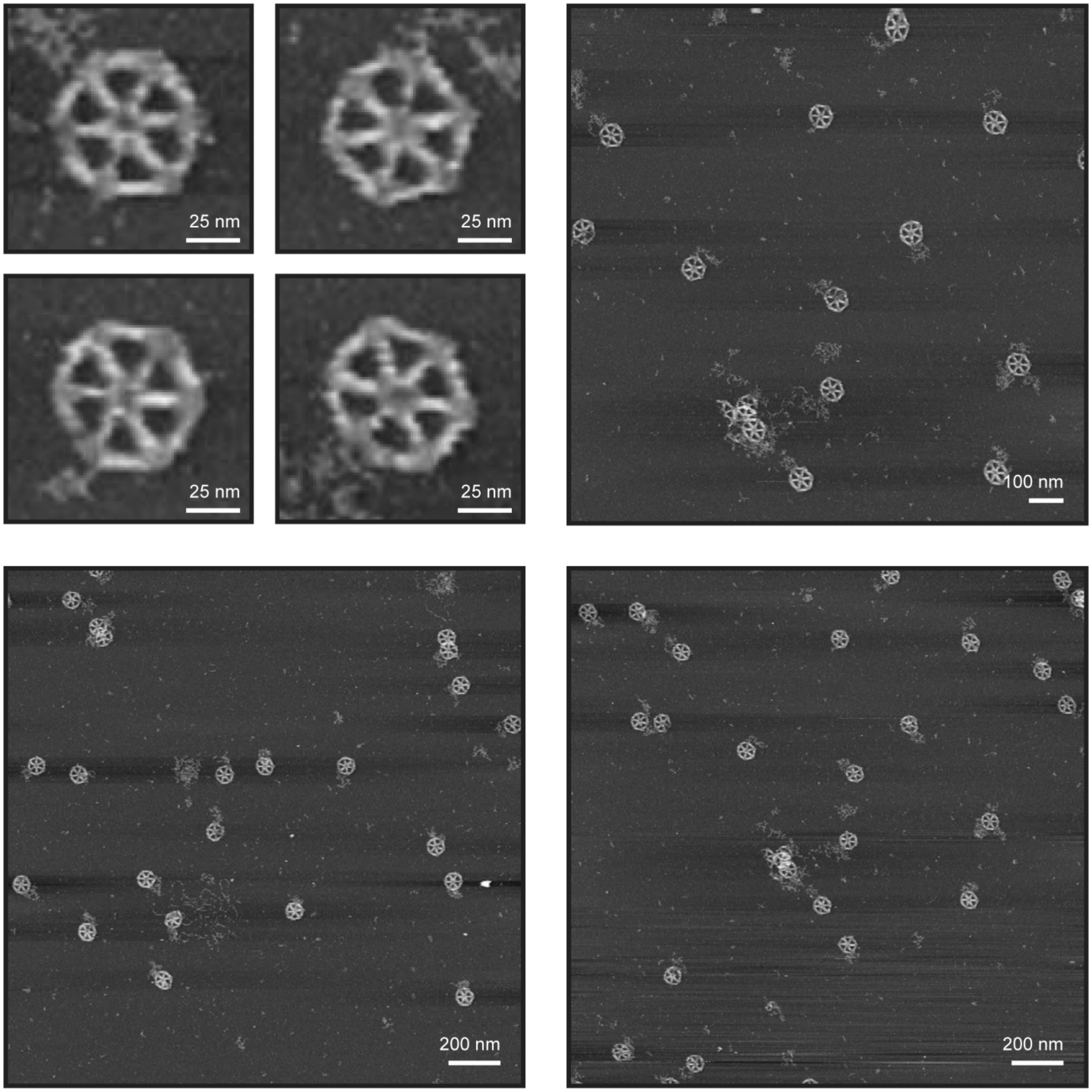

Supplementary Figure 13 AFM imaging of 6HB-based hexagonal DNA origami of 63-bp edge-length.

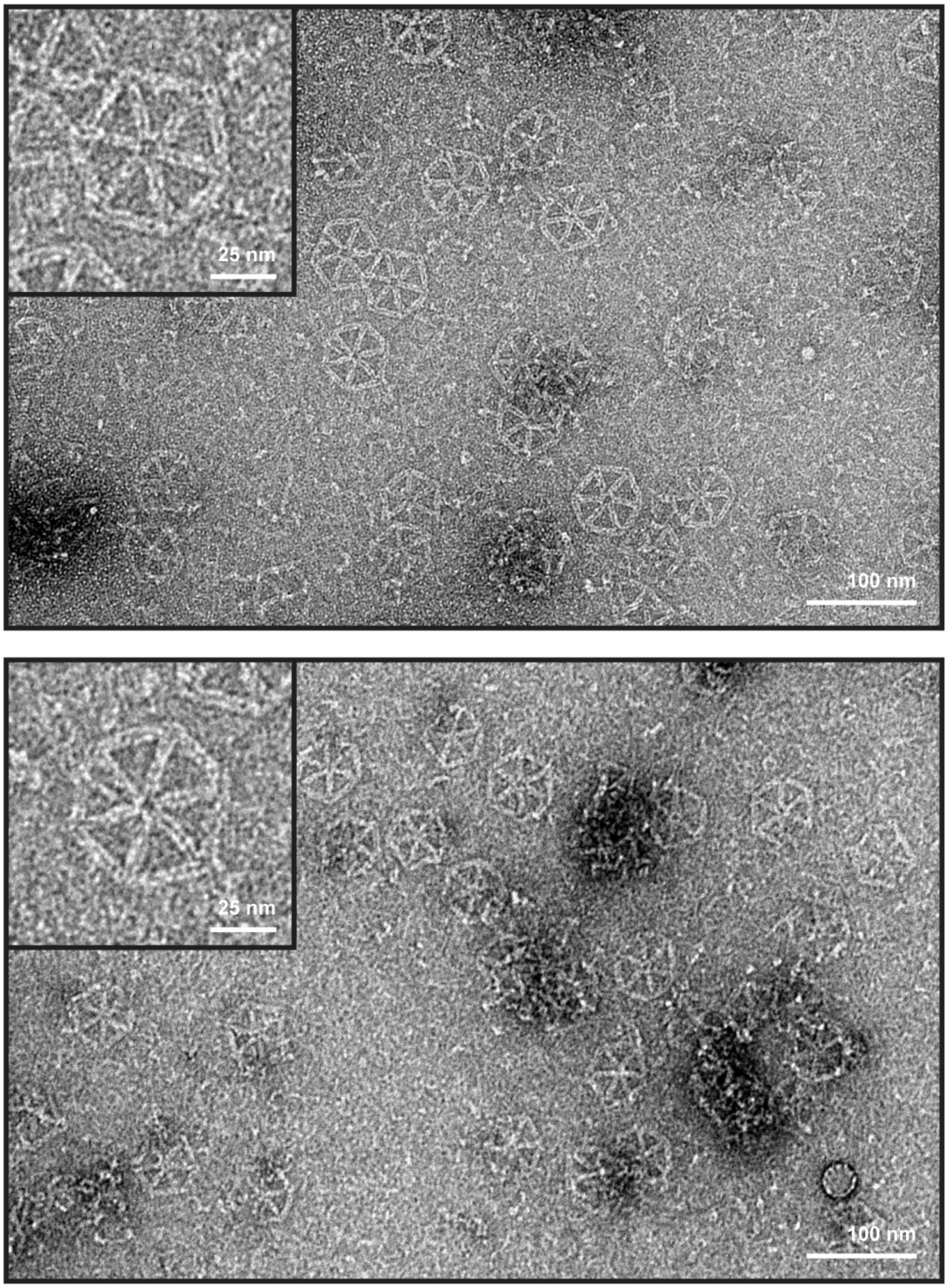

Supplementary Figure 14 TEM imaging the 6HB-based hexagonal DNA origami of 63-bp edge-length.

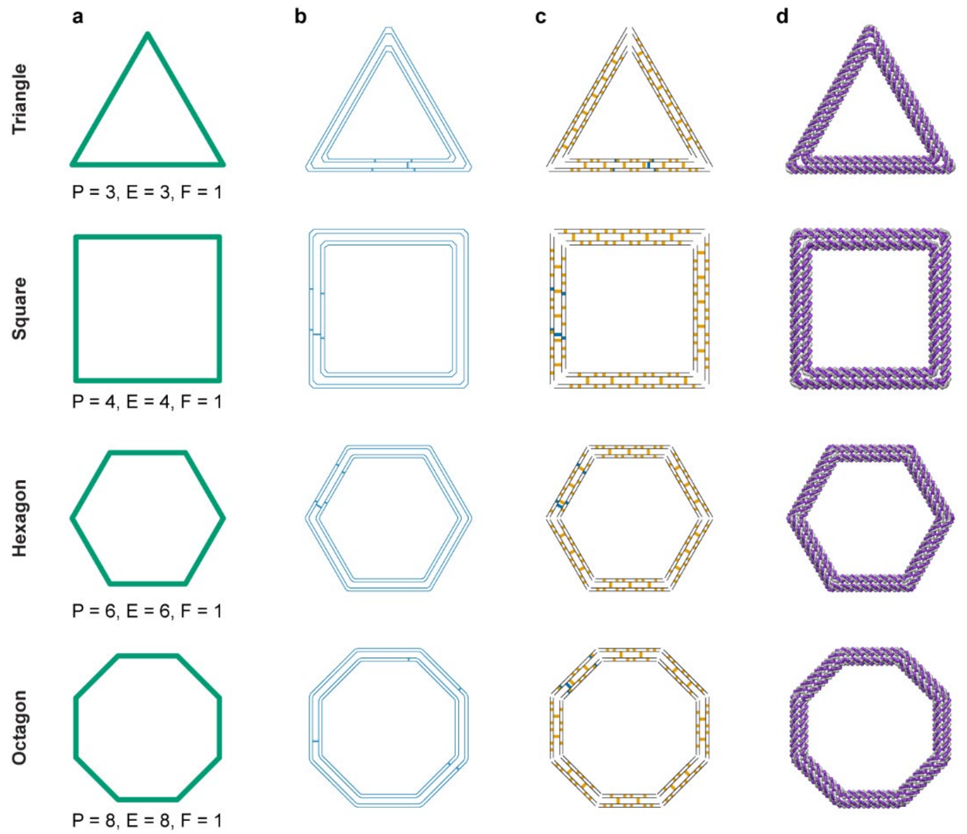

Supplementary Figure 15 Target geometries, scaffold routing paths, crossover models, and atomic models for the triangle, square, hexagon, and octagon without internal mesh. **a** The target geometries. The letters, P, E, and F denote the number of points, edges, and faces, respectively. **b** Scaffold routing path. The continuous blue loop is the single-stranded DNA scaffold that routes throughout the entire origami object of arbitrary shape. **c** Scaffold double crossovers (blue) determined by the spanning tree algorithm and staple double crossovers (orange). **d** Atomic model.

Supplementary Table 2 Design parameters for the triangle, square, hexagon, and octagon without internal mesh. # indicates the type of scaffolds in **Supplementary Table 4**.

Edge type	Target geometry	**Edge length**	**Scaffold**			**Staples**		
			Required length	# of double crossovers	# of unpaired nucleotides	# of staples	# of double crossovers	# of unpaired nucleotides
**DX**	Triangle	128-bp	855-nt (#2)	1	24	18	17	30
	Hexagon	74-bp	954-nt (#2)	1	24	18	17	42
**6HB**	Triangle	128-bp	2,772-nt (#2)	5	69	64	106	87
	Square	105-bp	2,908-nt (#1)	5	96	68	115	100
	Hexagon	74-bp	3,042-nt (#1)	5	120	69	115	66
	Octagon	57-bp	3,072-nt (#1)	5	136	69	109	120

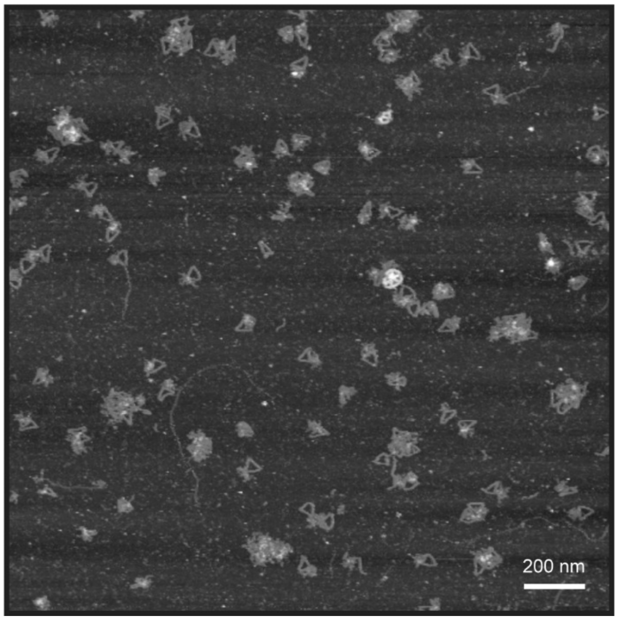

Supplementary Figure 16 AFM imaging of DX-based triangular DNA origami of 128-bp edge-length without internal mesh.

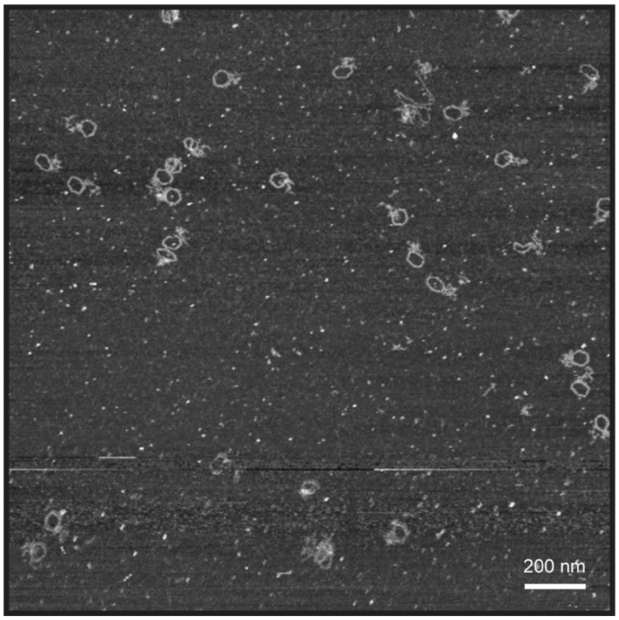

Supplementary Figure 17 AFM imaging of DX-based hexagonal DNA origami of 74-bp edge-length without internal mesh.

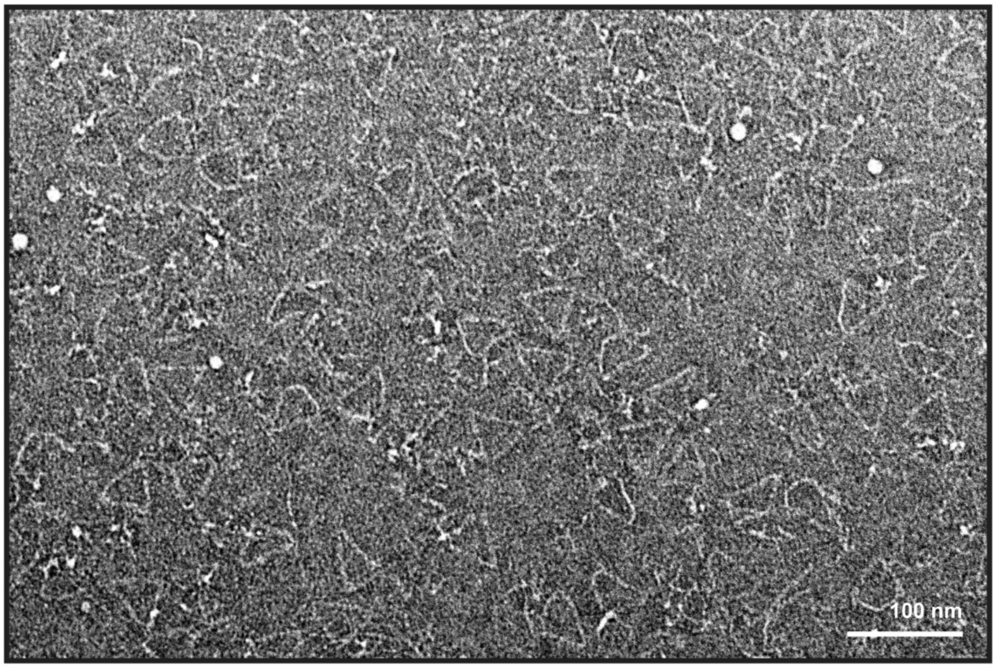

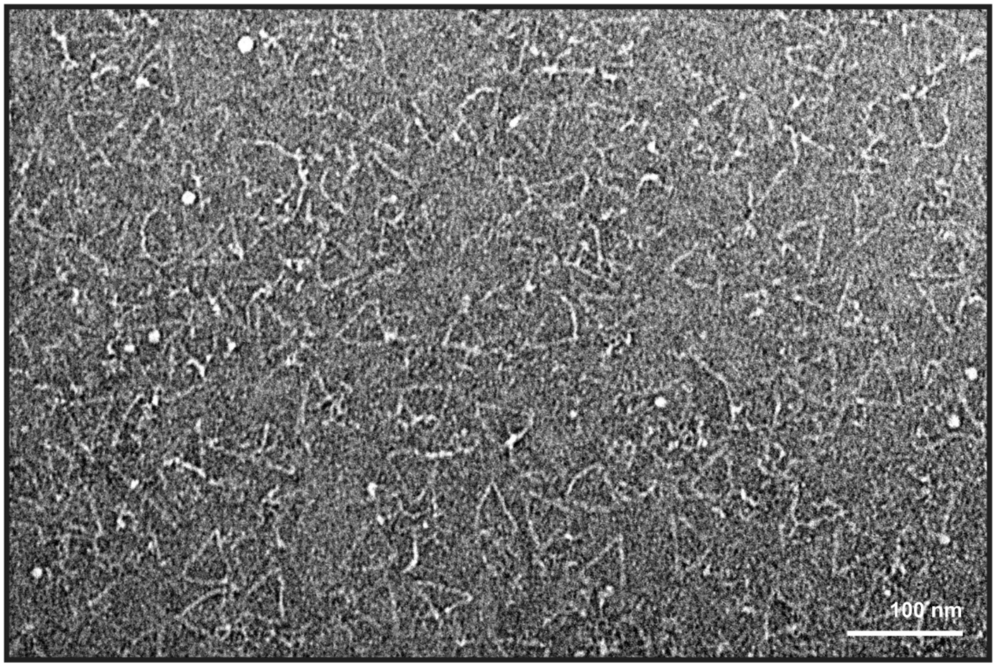

Supplementary Figure 18 TEM imaging of DX-based triangular DNA origami of 128-bp edge-length without internal mesh.

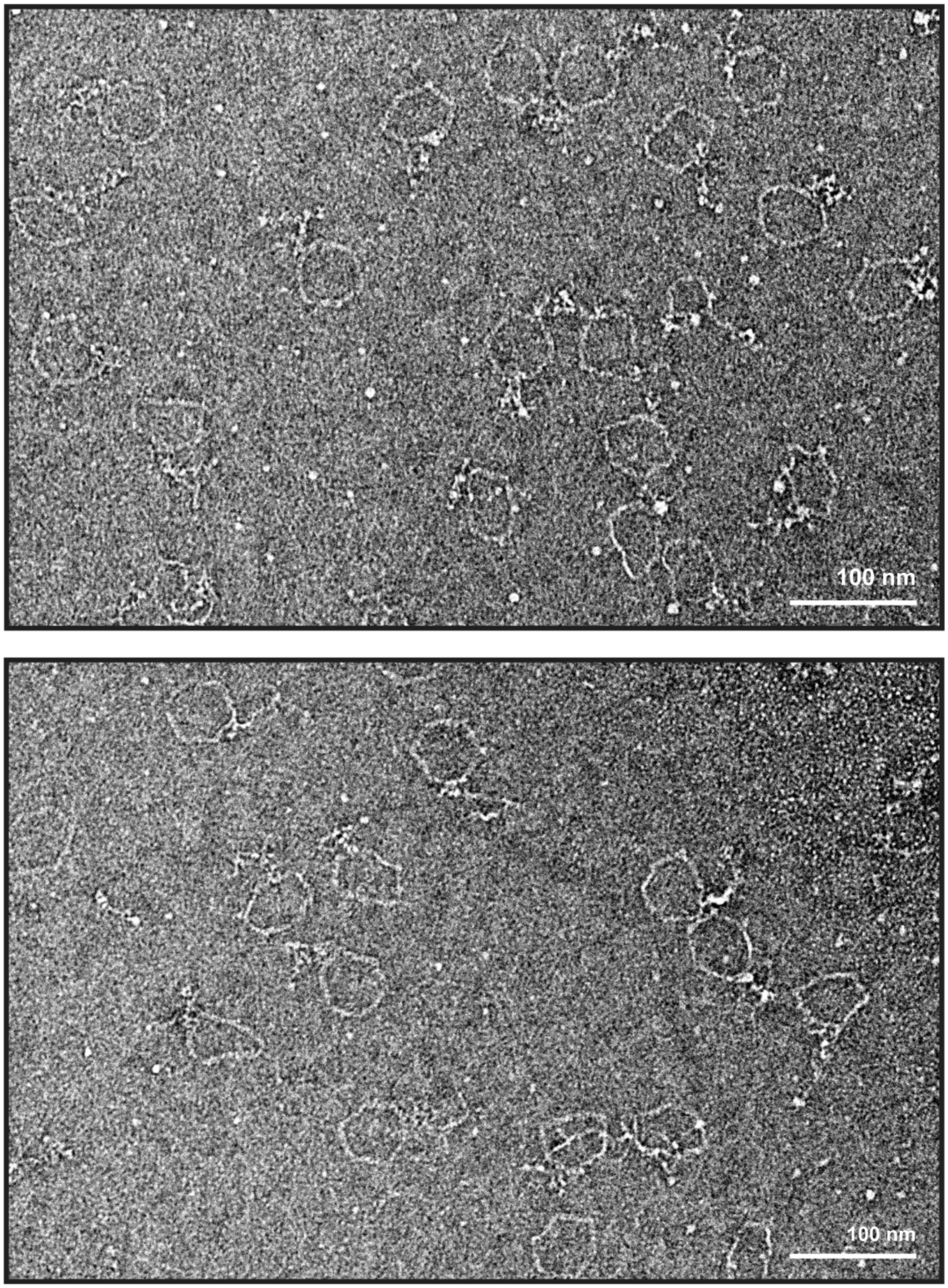

Supplementary Figure 19 TEM imaging of DX-based hexagonal DNA origami of 74-bp edge-length without internal mesh.

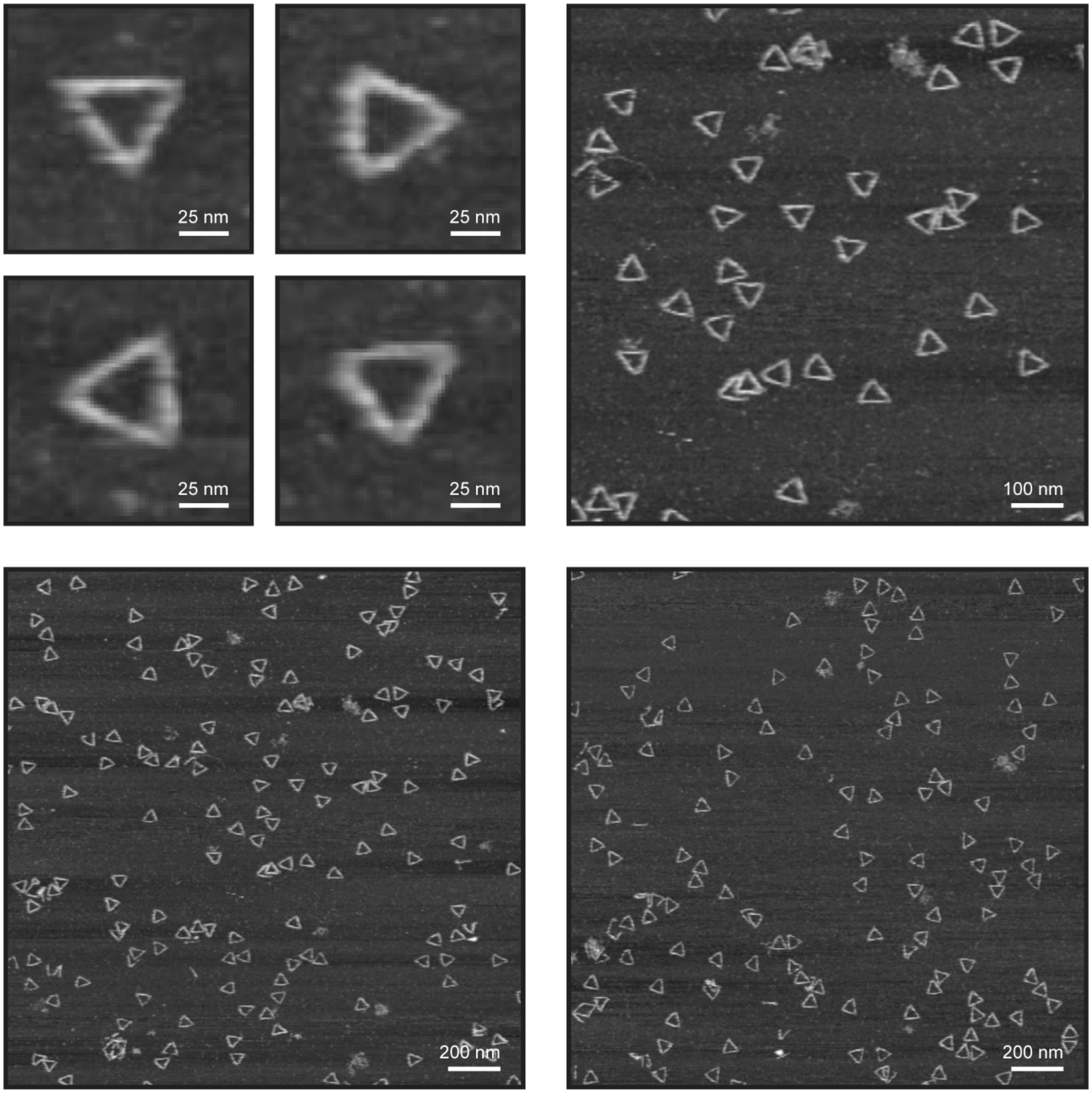

Supplementary Figure 20 AFM imaging of 6HB-based triangular DNA origami of 128-bp edge-length without internal mesh.

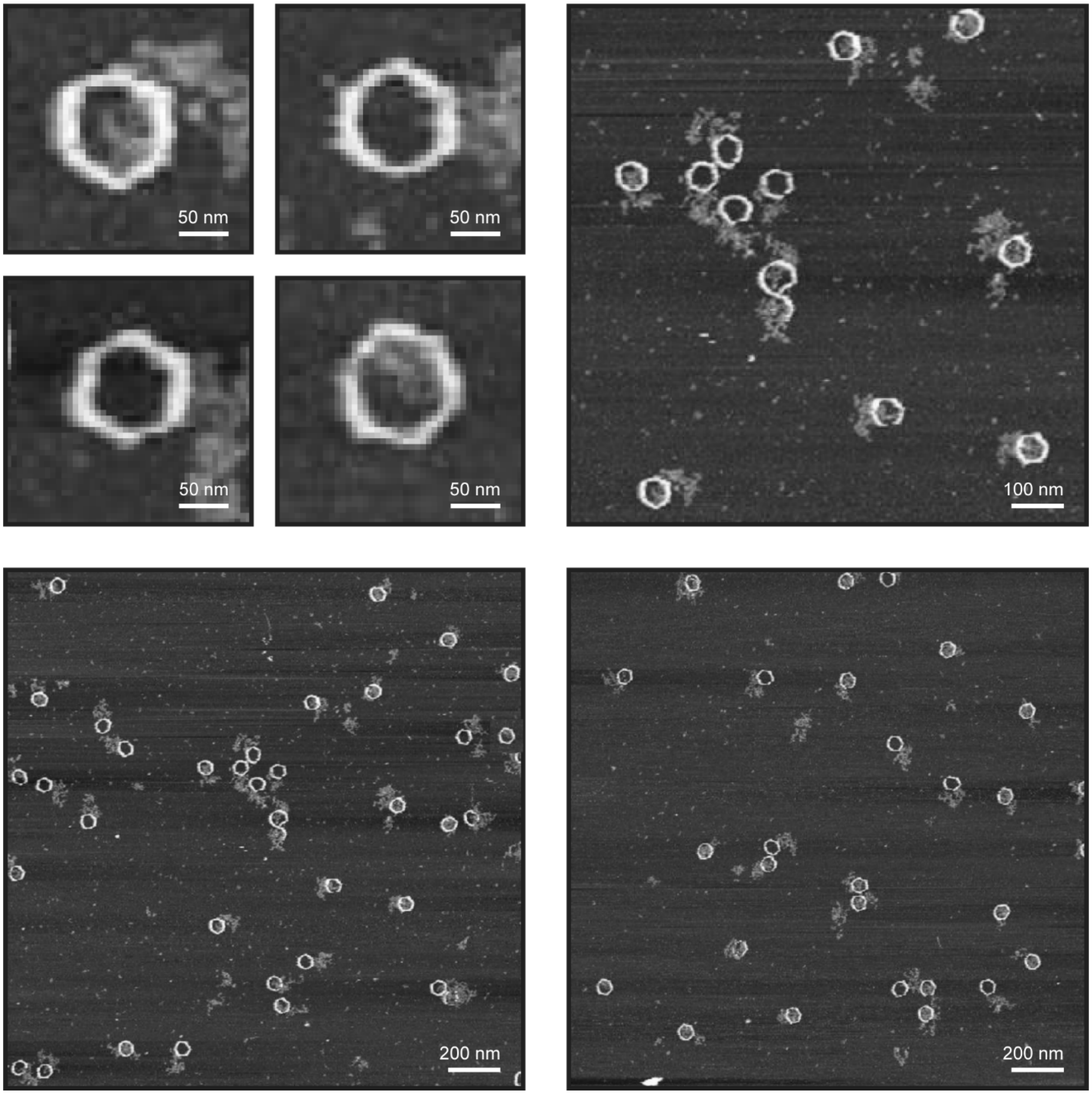

Supplementary Figure 21 AFM imaging of 6HB-based hexagonal DNA origami of 74-bp edge-length without internal mesh.

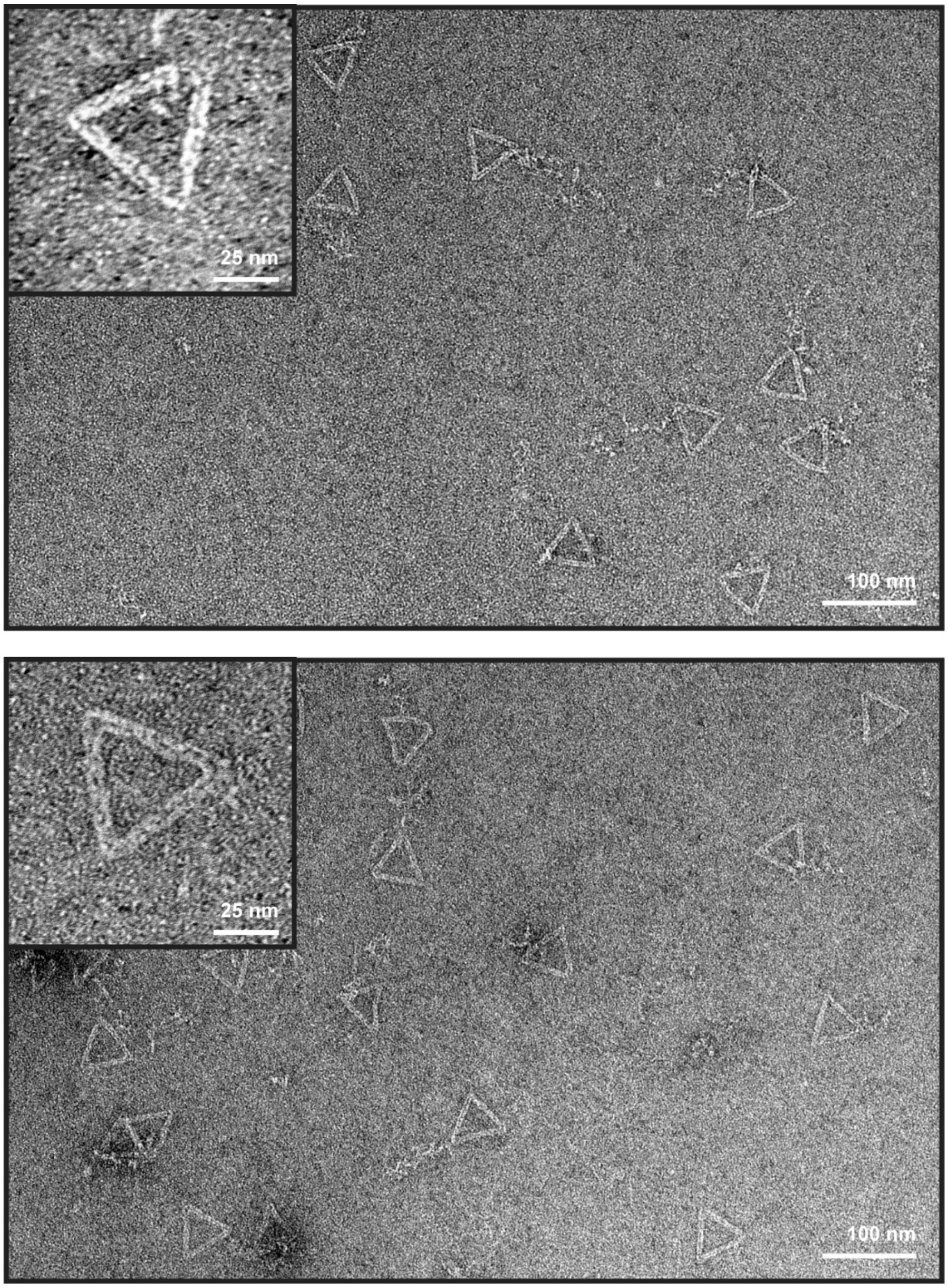

Supplementary Figure 22 TEM imaging of 6HB-based triangular DNA origami of 128-bp edge-length without internal mesh.

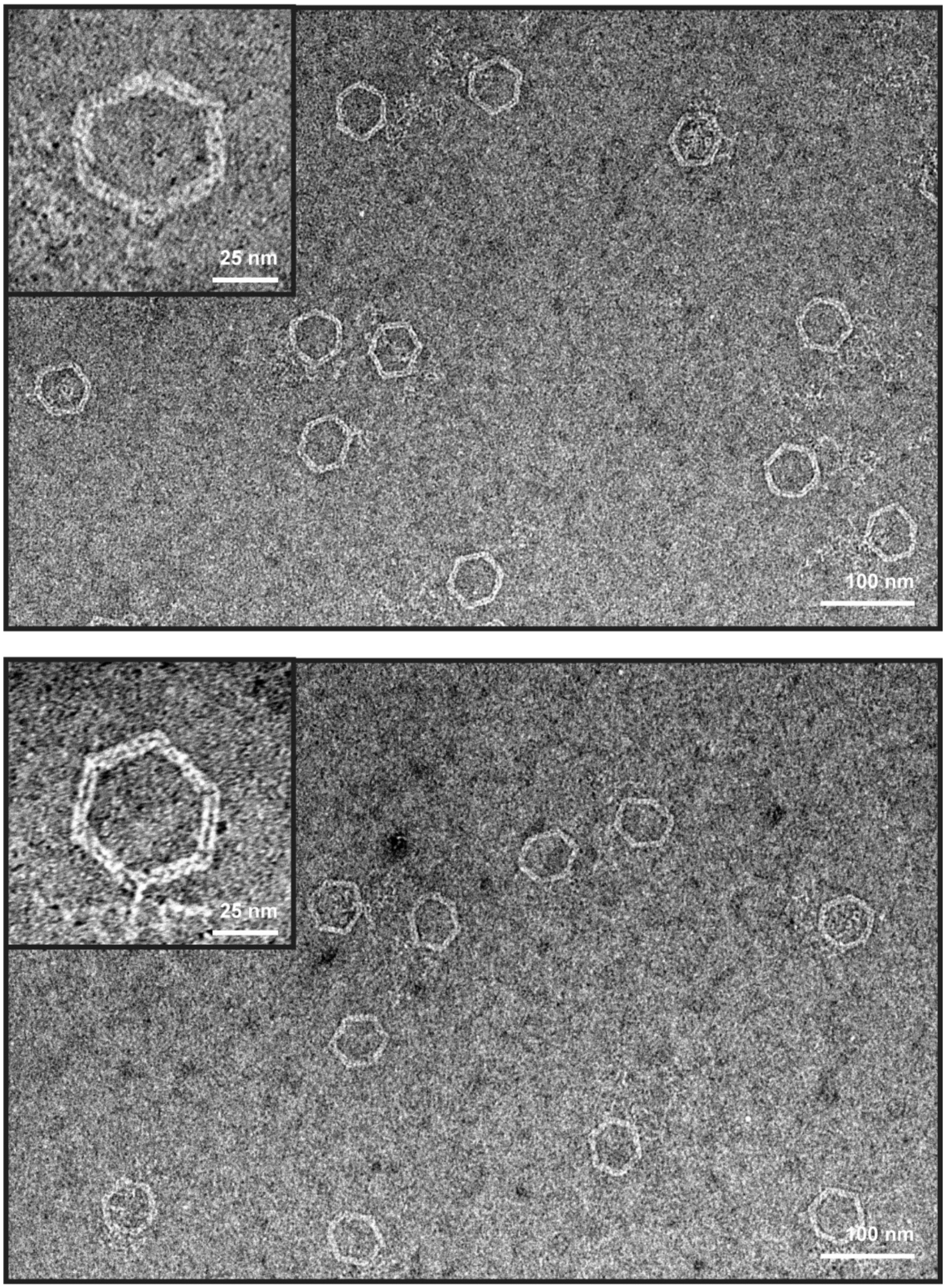

Supplementary Figure 23 TEM imaging of 6HB-based hexagonal DNA origami of 74-bp edge-length without internal mesh.

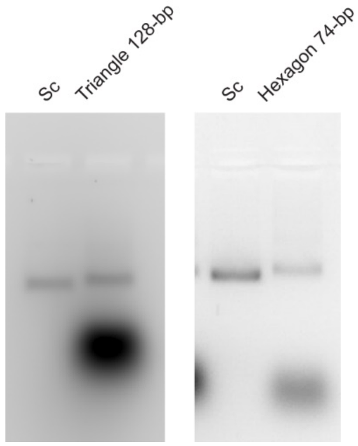

Supplementary Figure 24 Agarose gel electrophoresis for 6HB-based triangular and hexagonal DNA origami objects without internal mesh.

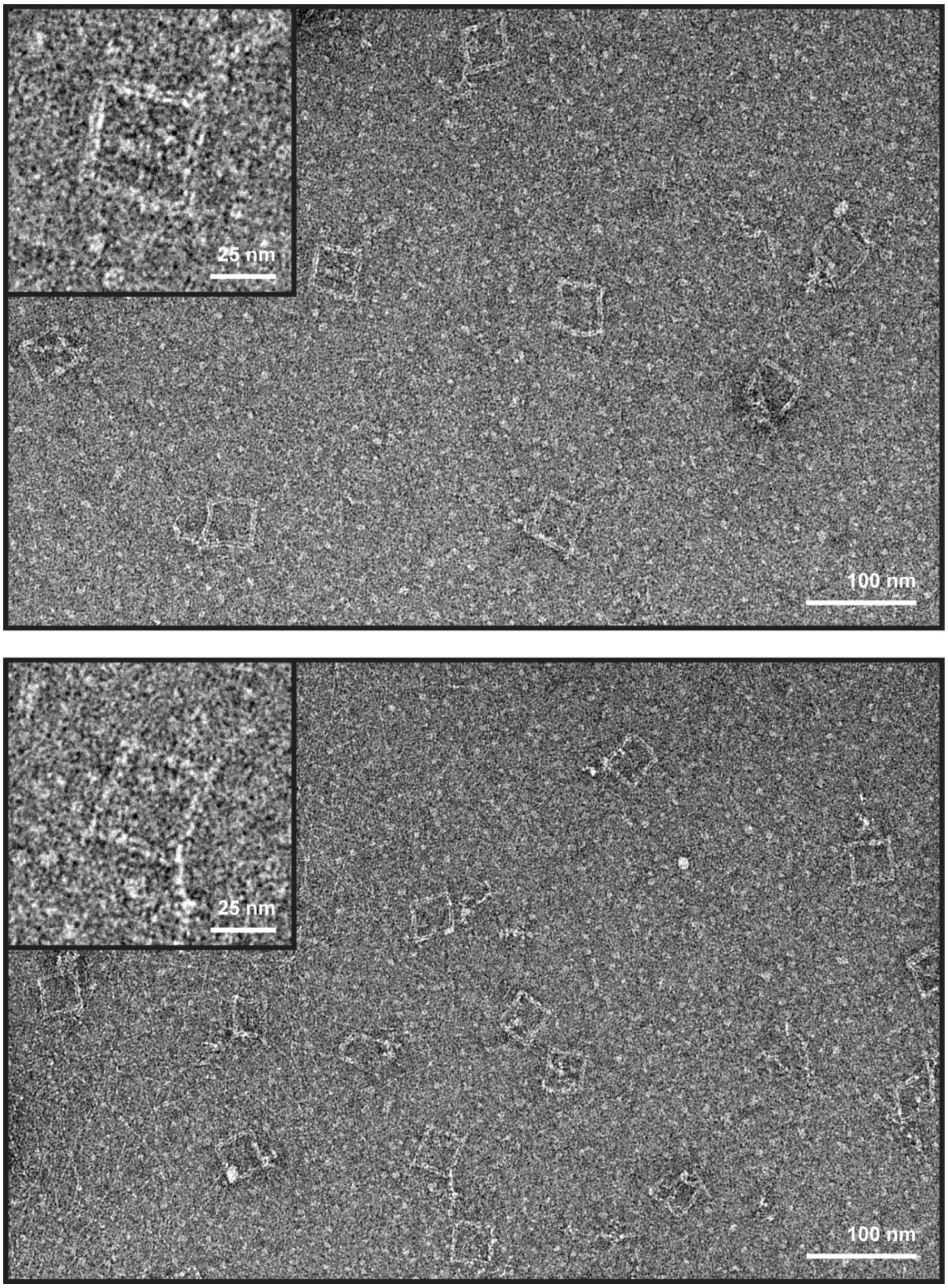

Supplementary Figure 25 TEM imaging of 6HB-based square DNA origami of 105-bp edge-length without internal mesh.

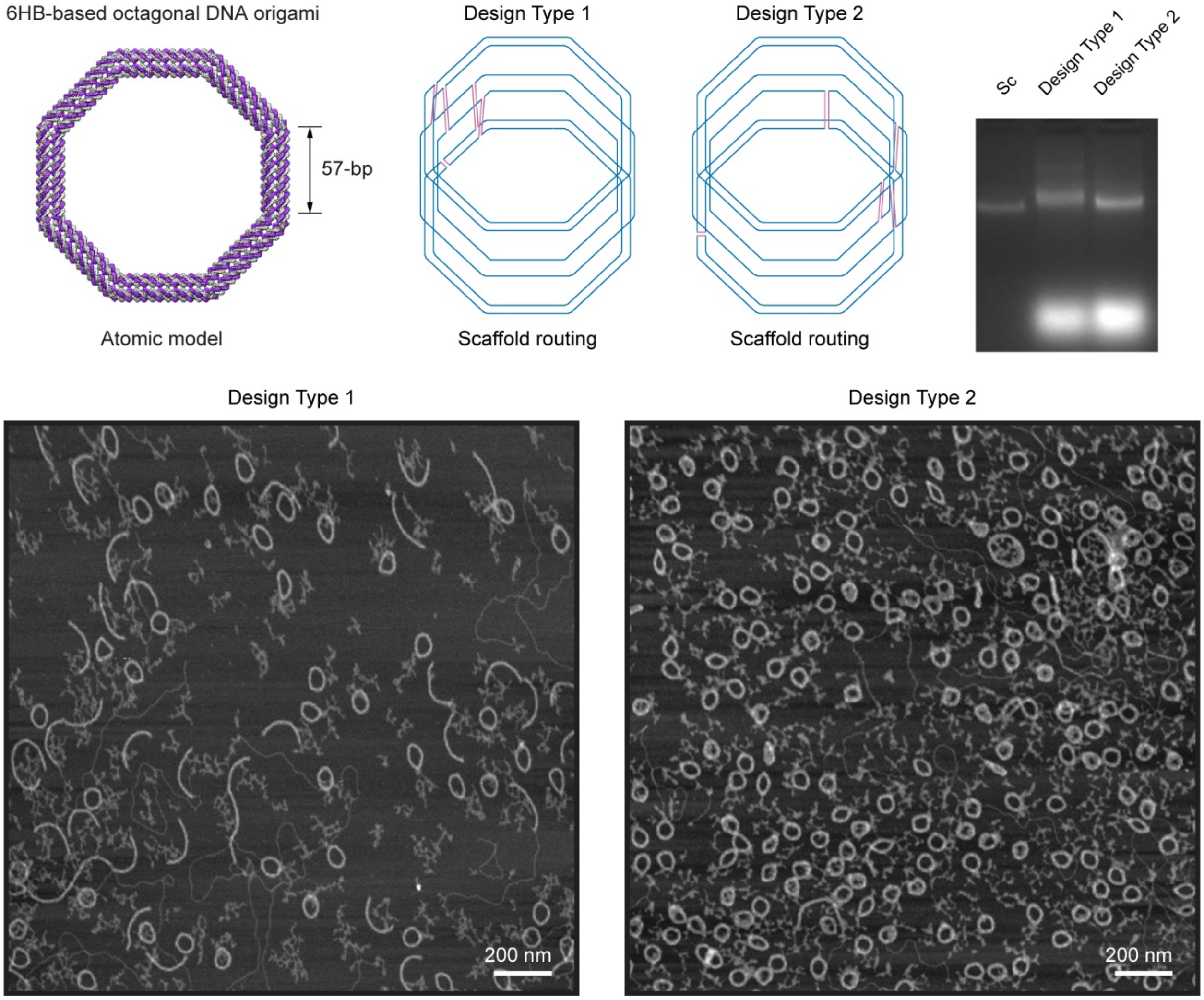

Supplementary Figure 26 Two designs, agarose gel electrophoresis, and AFM imaging of 6HB-based octagonal DNA origami of 57-bp edge-length without internal mesh. All scaffold double-crossovers are located on the same edge in Design Type 1 versus distributed to different edges in Design Type 2.

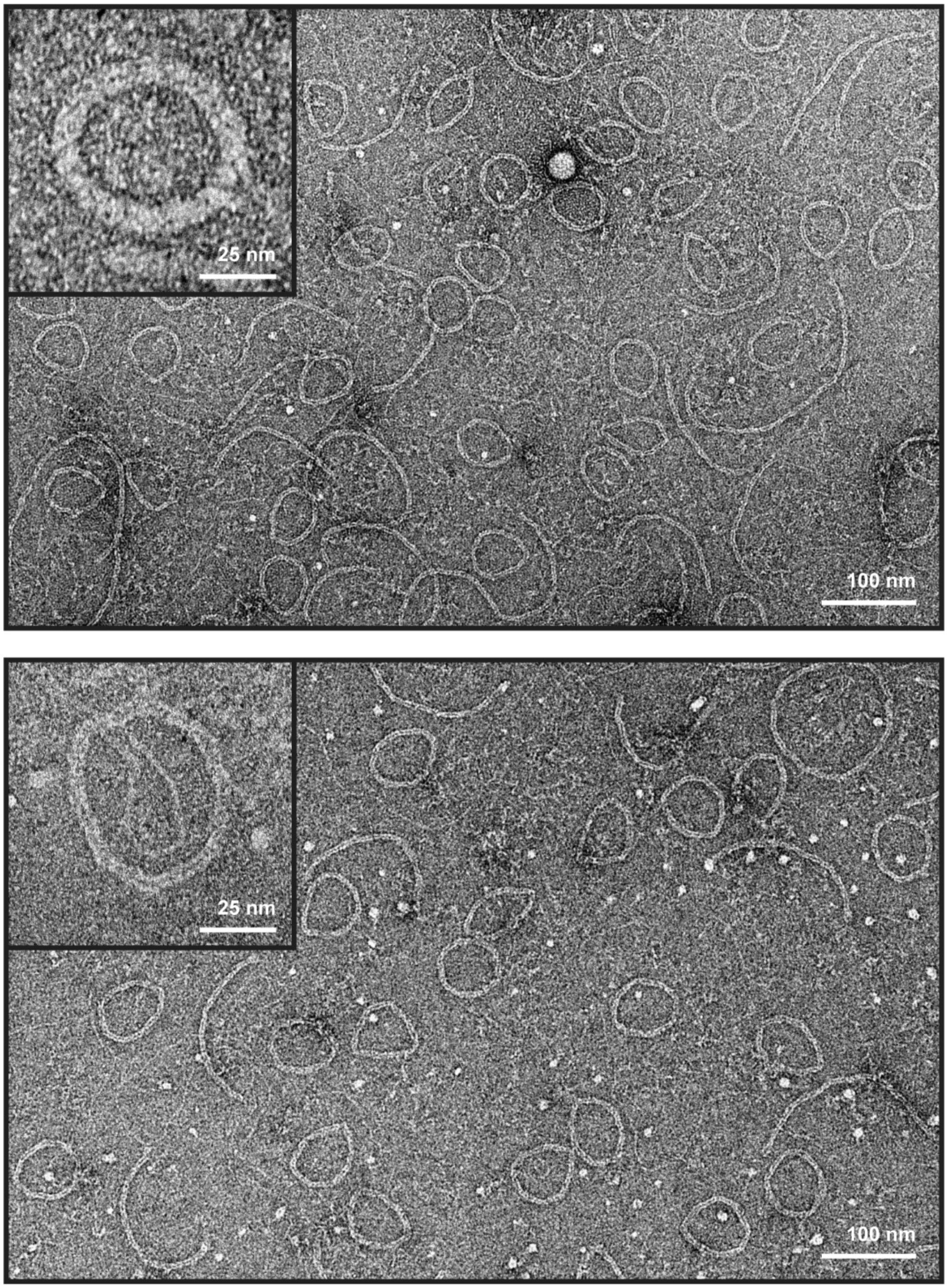

Supplementary Figure 27 TEM imaging of 6HB-based octagonal DNA origami of 57-bp edge-length without internal mesh when using the Design Type 1 shown in **Supplementary Fig. 26**.

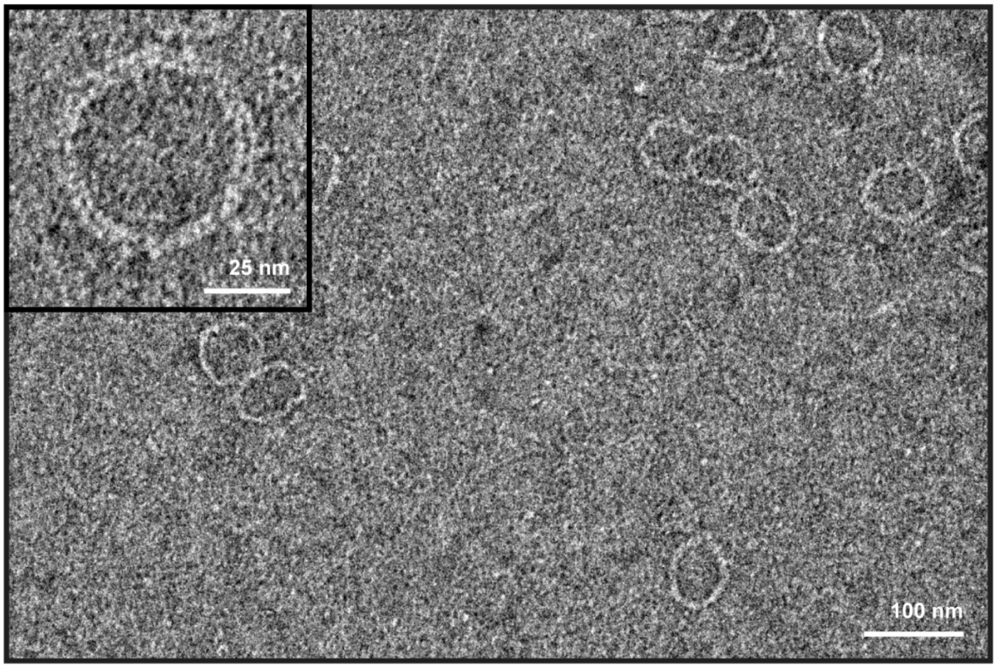

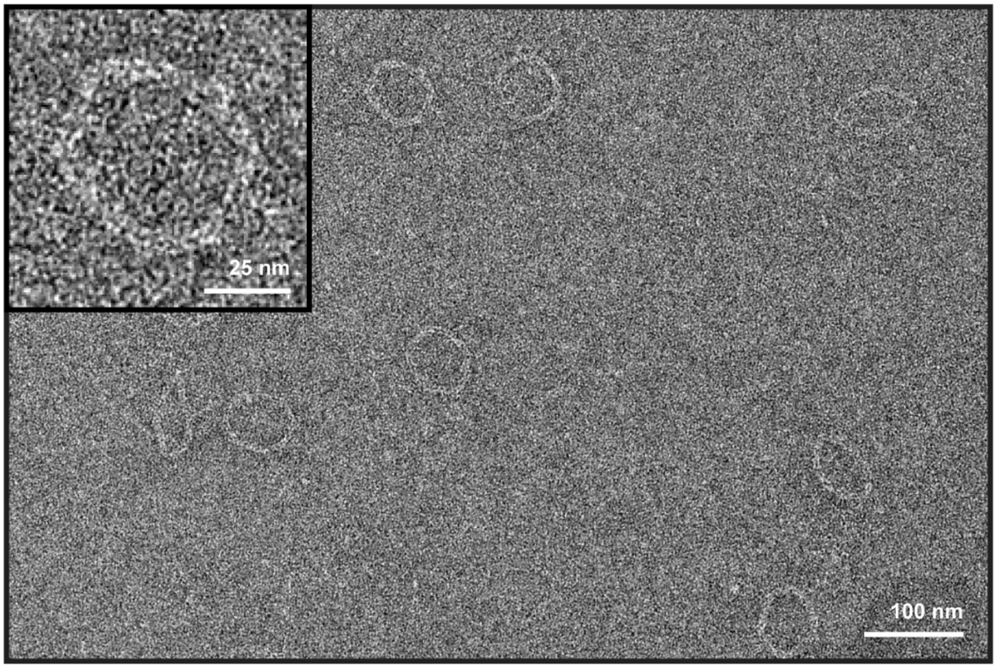

Supplementary Figure 28 TEM imaging of 6HB-based octagonal DNA origami of 57-bp edge-length without internal mesh when using Design Type 2 shown in **Supplementary Fig. 26**.

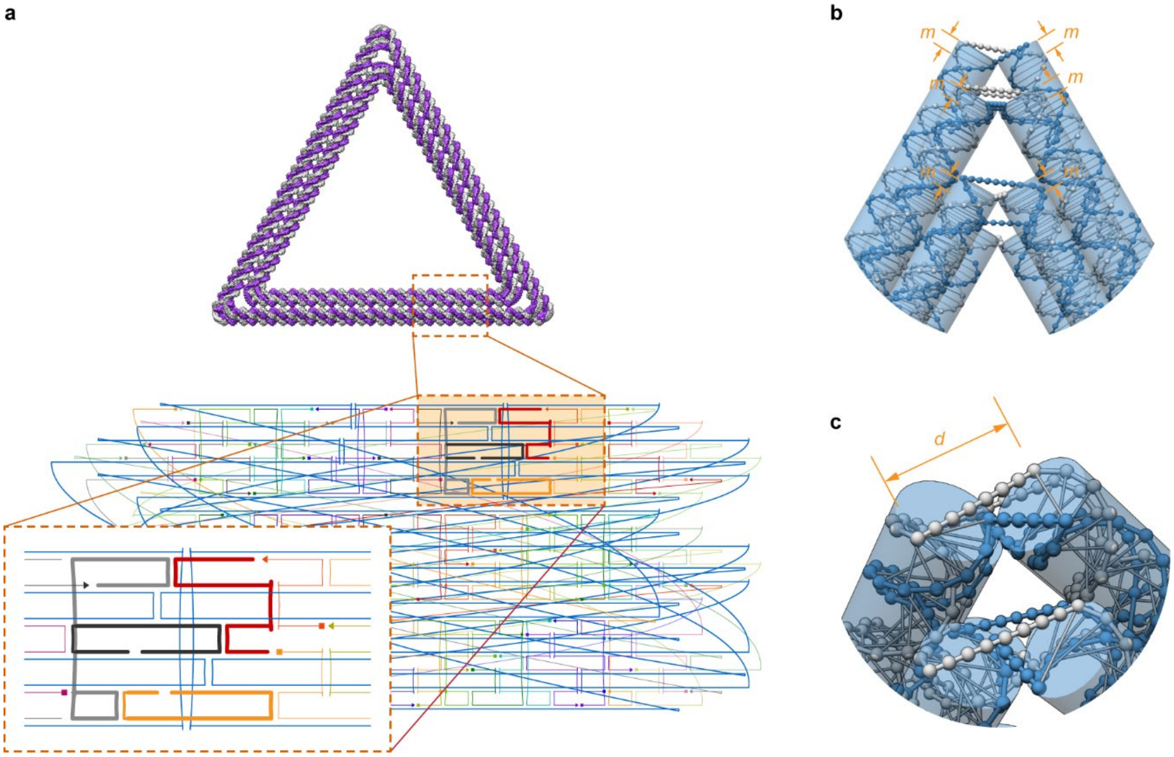

Supplementary Figure 29 6HB-based open triangular DNA origami. **a** Four staples (gray, red, black and orange thick lines in the caDNAno map) on one edge of the triangle where three scaffold double-crossovers exists were removed. The stability of programmed internal vertex angles was evaluated by **b** reducing the mitered edge length (*m*) by 2-bp and **c** changing the parameter to determine axial rise per nucleotide in both the scaffold and staple loop (the axial rise is calculated by dividing the distance (*d*) by the design parameter).

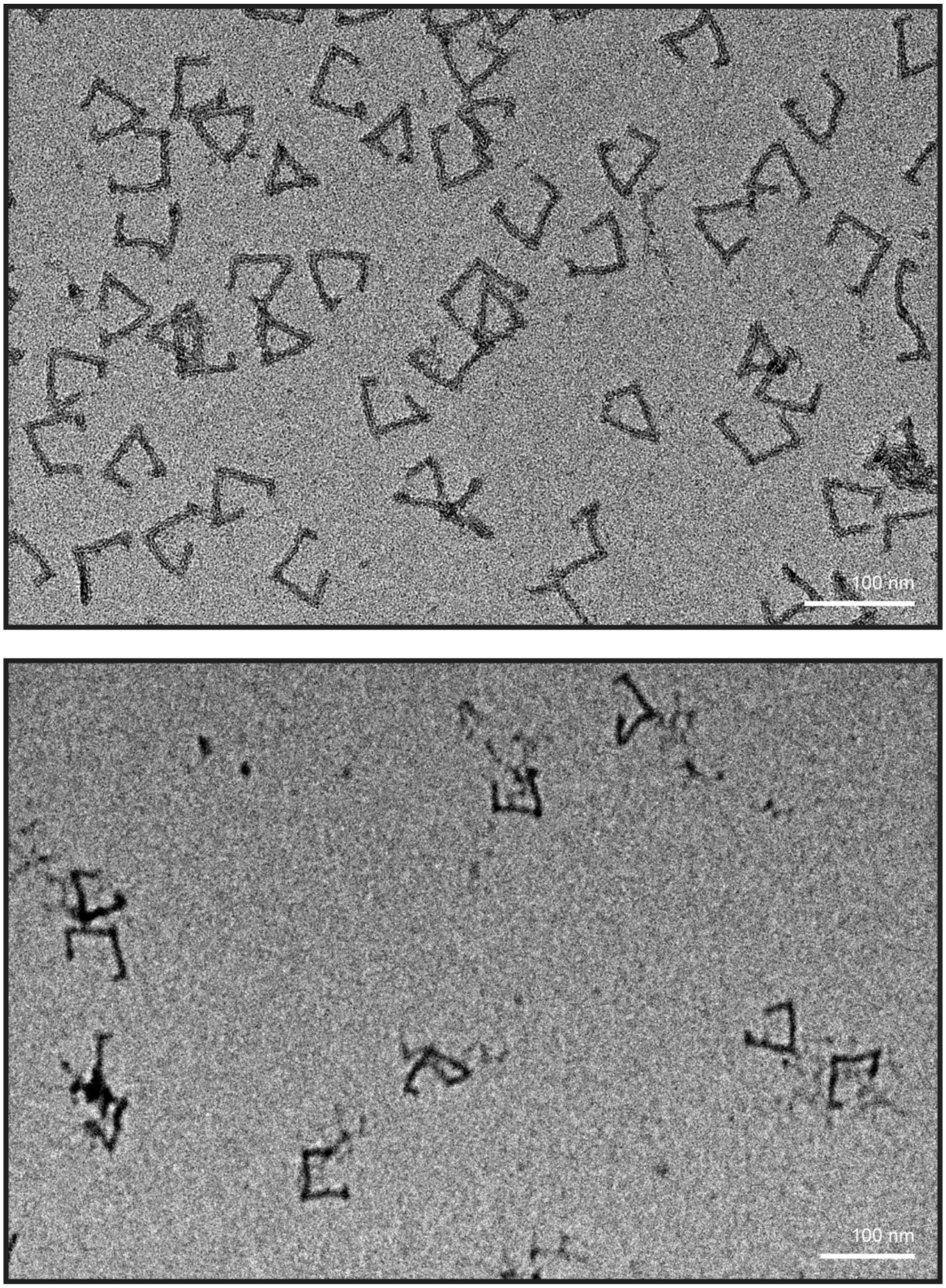

Supplementary Figure 30 TEM imaging of the DNA open triangular object (Top) and with reducing the mitered edge length *m* (**Supplementary Fig. 26b**) as 2-bp (Bottom).

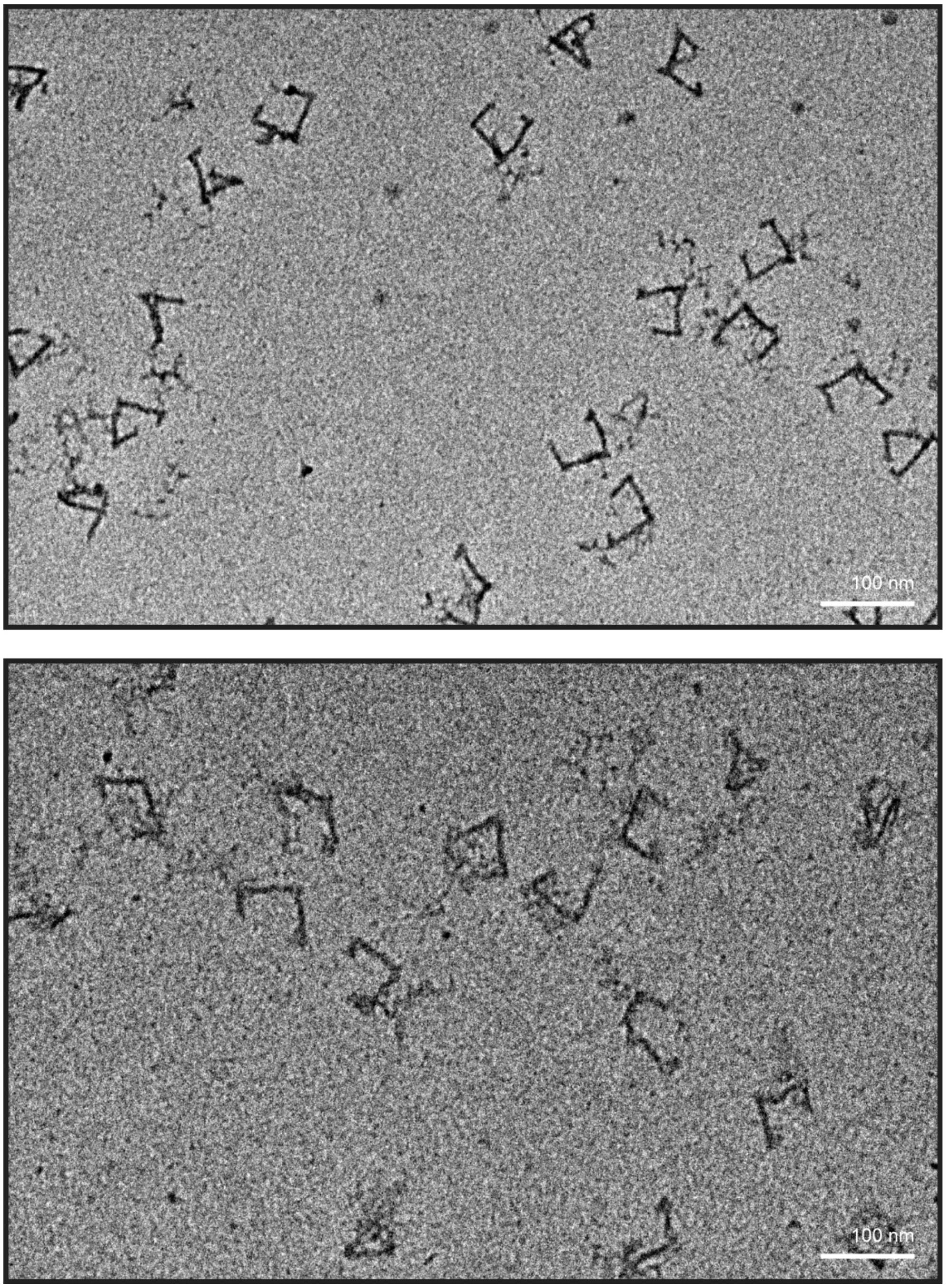

Supplementary Figure 31 TEM imaging of DNA open triangles with the different number of unpaired nucleoids crossing the vertex. The number of unpaired nucleotides is calculated by dividing the distance *d* (**Supplementary Fig. 26c**) with the parameter 0.34 (top) and 0.55 (bottom).

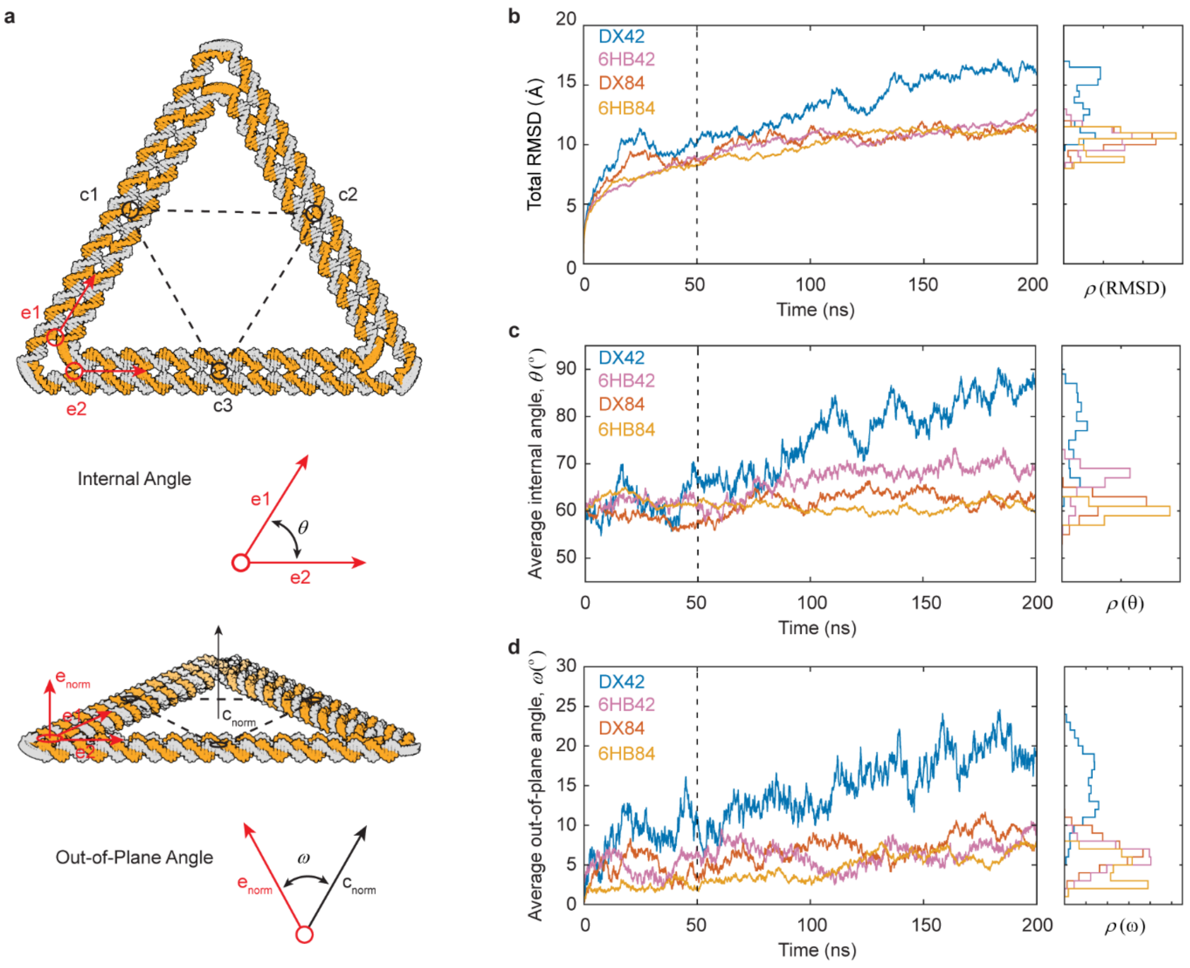

Supplementary Figure 32 Molecular dynamics trajectory data. **a** Schematic illustrating the calculation of the (top) internal angles, θ, and (bottom) out-of-plane angles, ω, of a wireframe DNA origami object. **b** The total RMSD of each triangular wireframe object over 200 ns MD simulation (left), and a histogram of the total RMSD values after t = 50 ns equilibration time. **c** The average internal angle, θ, of each triangular wireframe object over 200 ns MD simulation time (left), and a histogram of the internal angles after t = 50 ns equilibration time. **d** The average out-of-plane angle, ω, of each triangular wireframe object over 200 ns MD simulation time (left), and a histogram of the internal angles after t = 50 ns equilibration time.

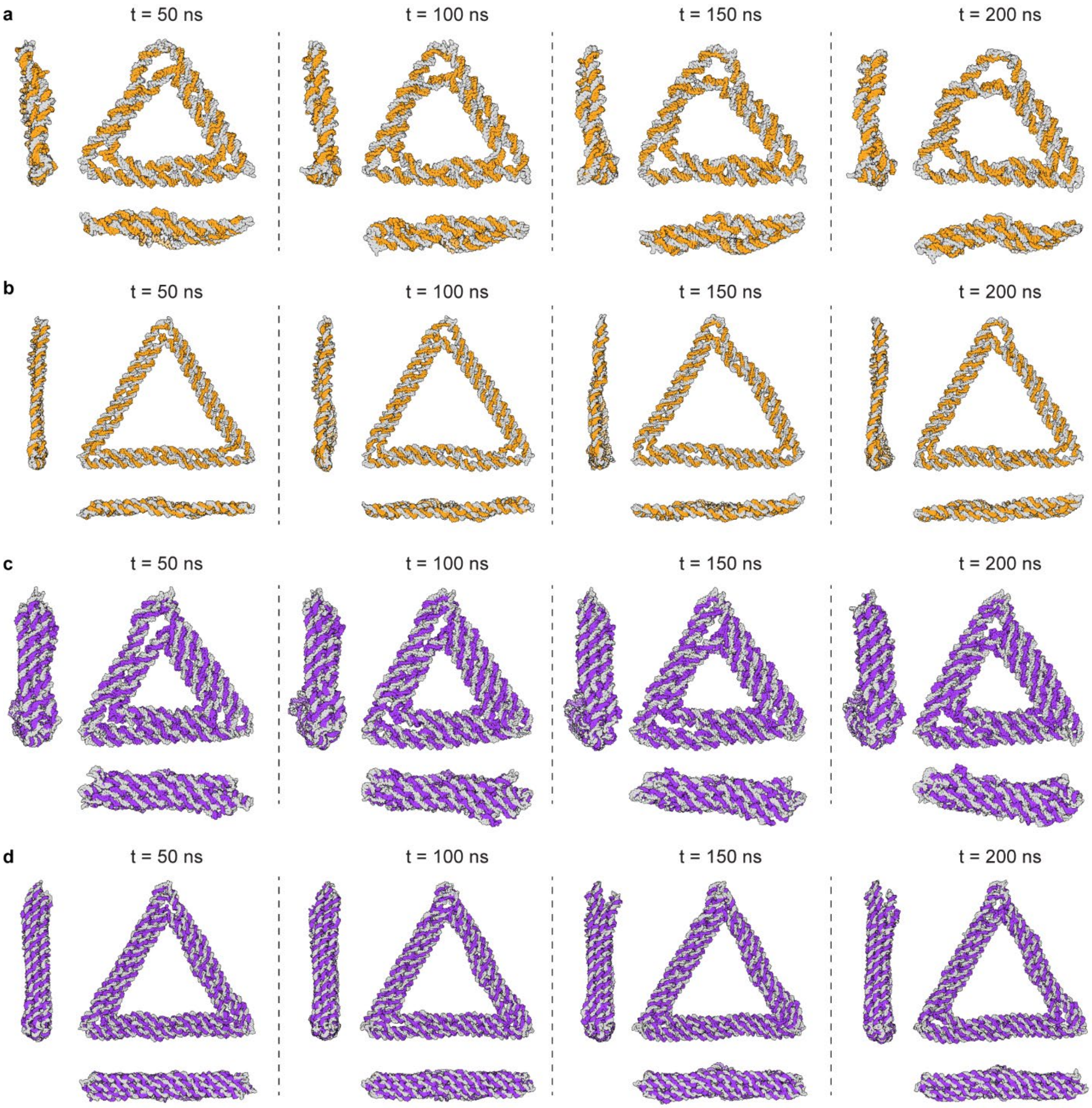

Supplementary Figure 33 Instantaneous configurations of the triangular origami objects from MD simulations. **a** DX-based DNA triangular origami with 42-bp edge-length **b** DX-based DNA triangular origami with 84-bp edge-length **c** 6HB-based DNA triangular origami with 42-bp edge-length **d** 6HB-based DNA triangular origami with 84-bp edge-length.

Supplementary Table 3 Design parameters for the 6HB-based star and curved beam DNA origami objects. # indicates the type of scaffolds in **Supplementary Table 4**.

Target geometry	Edge length	Scaffold			Staples		
		Required length	# of double crossovers	# of unpaired nucleotides	# of staples	# of double crossovers	# of unpaired nucleotides
**Star**	42-bp	5,869-nt (#1)	20	295	128	184	374
**Curved beam**	42-bp	6,869-nt (#1)	20	331	154	211	392
**Hexagon with a hole**	42-bp	12,096-nt	59	198	282	361	1,128
**Circle**	42-bp	16,029-nt	74	863	364	427	1,258
**Quarter circle**	42-bp	11,354-nt	26	393	264	402	496
**Annulus**	42-bp	12,013-nt	35	573	265	376	767
**A-shape**	42-bp	13,831-nt	35	551	311	471	605
**G-shape**	42-bp	16,662-nt	41	690	375	561	791

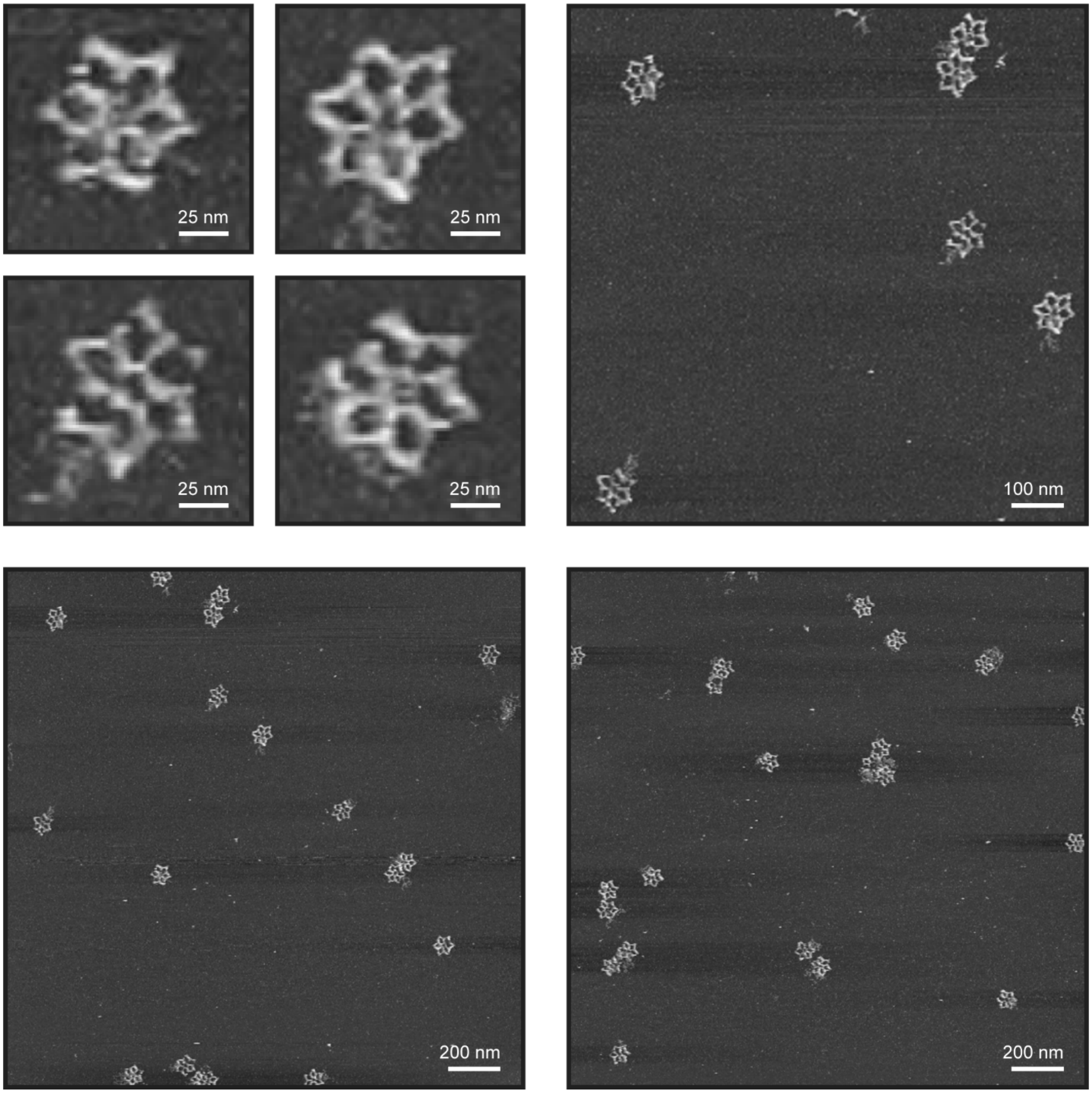

Supplementary Figure 34 AFM imaging of 6HB-based star DNA origami of 42-bp edge-length.

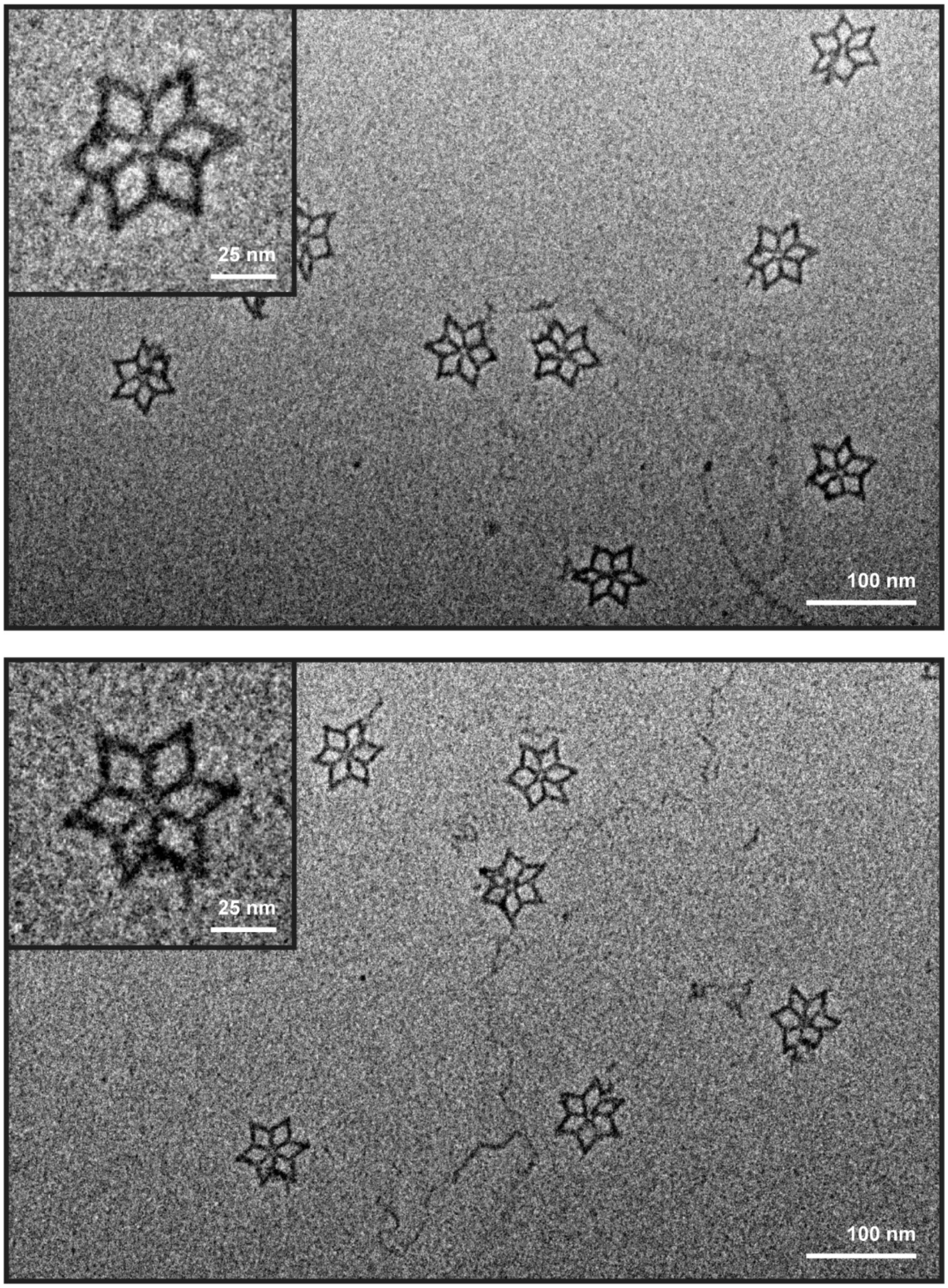

Supplementary Figure 35 TEM imaging of 6HB-based star DNA origami of 42-bp edge-length.

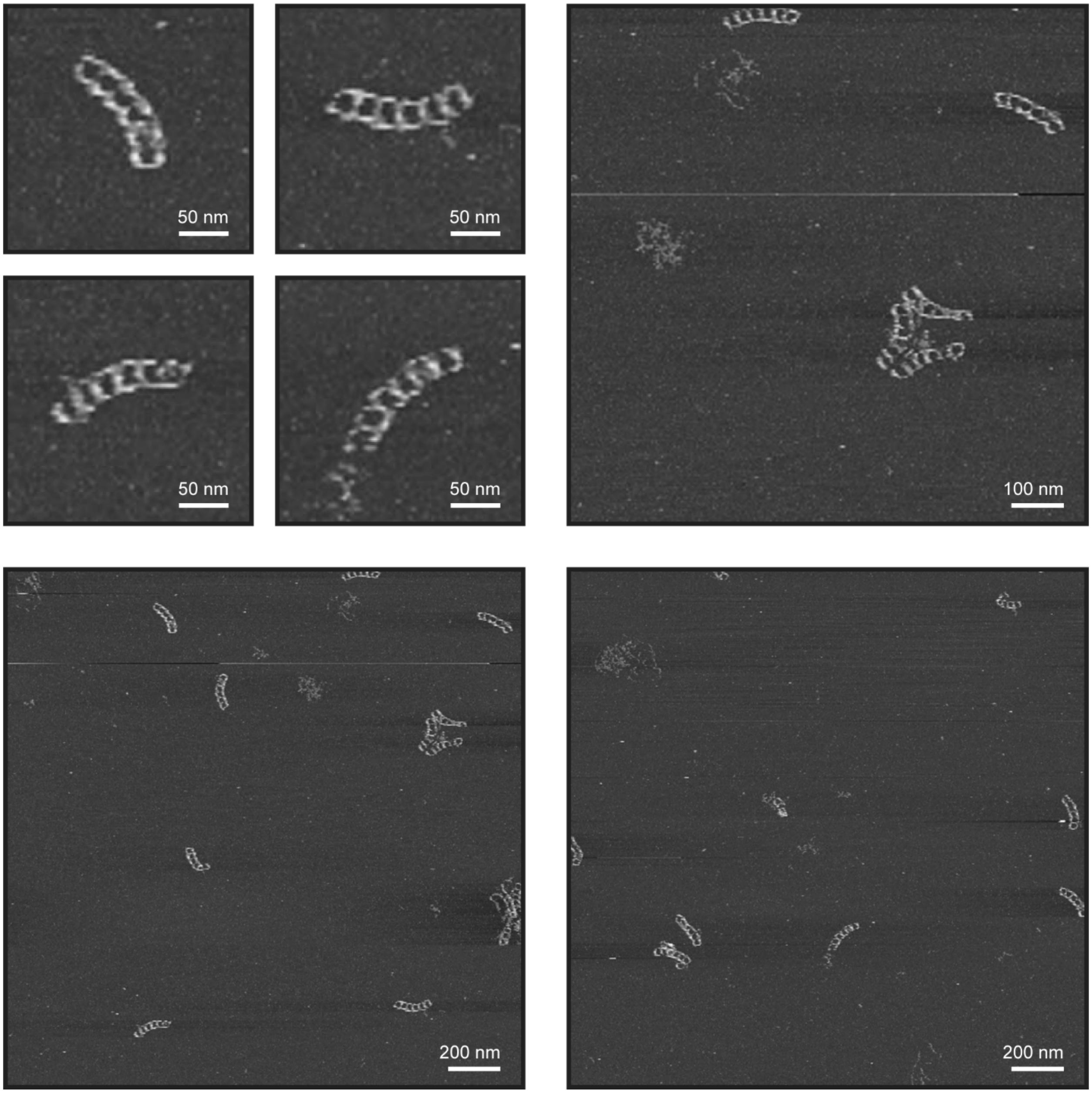

Supplementary Figure 36 AFM imaging of 6HB-based curved beam DNA origami of 42-bp edge-length.

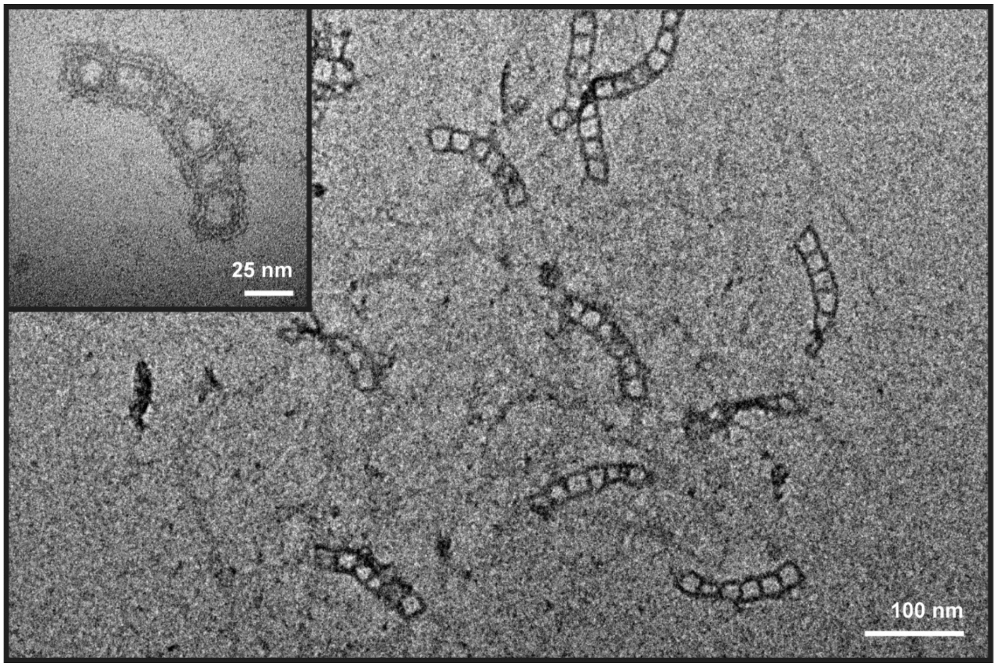

Supplementary Figure 37 TEM imaging of 6HB-based curved beam DNA origami of 42-bp edge-length.

Supplementary Table 4 Sequences for the 7,249-nt (#1) and 2,775-nt (#2) length scaffolds used.

No	Scaffold sequence
#1	AATGCTACTACTATTAGTAGAATTGATGCCACCTTTTCAGCTCGCGCCCCAAATGAAAATATAGCTAAACAGGTTATTGACC<br>ATTTGCGAAATGTATCTAATGGTCAAACTAAATCTACTCGTTCGCAGAATTGGGAATCAACTGTTATATGGAATGAAACTTC<br>CAGACACCGTACTTTAGTTGCATATTTAAAACATGTTGAGCTACAGCATTATATTCAGCAATTAAGCTCTAAGCCATCCGCA<br>AAAATGACCTCTTATCAAAAGGAGCAATTAAAGGTACTCTCTAATCCTGACCTGTTGGAGTTTGCTTCCGGTCTGGTTCGCT<br>TTGAAGCTCGAATTAAAACGCGATATTTGAAGTCTTTCGGGCTTCCTCTTAATCTTTTTGATGCAATCCGCTTTGCTTCTGA<br>CTATAATAGTCAGGGTAAAGACCTGATTTTTGATTTATGGTCATTCTCGTTTTCTGAACTGTTTAAAGCATTTGAGGGGGAT<br>TCAATGAATATTTATGACGATTCCGCAGTATTGGACGCTATCCAGTCTAAACATTTTACTATTACCCCCTCTGGCAAAACTT<br>CTTTTGCAAAAGCCTCTCGCTATTTTGGTTTTTATCGTCGTCTGGTAAACGAGGGTTATGATAGTGTTGCTCTTACTATGCC<br>TCGTAATTCCTTTTGGCGTTATGTATCTGCATTAGTTGAATGTGGTATTCCTAAATCTCAACTGATGAATCTTTCTACCTGT<br>AATAATGTTGTTCCGTTAGTTCGTTTTATTAACGTAGATTTTTCTTCCCAACGTCCTGACTGGTATAATGAGCCAGTTCTTA<br>AAATCGCATAAGGTAATTCACAATGATTAAAGTTGAAATTAAACCATCTCAAGCCCAATTTACTACTCGTTCTGGTGTTTCT<br>CGTCAGGGCAAGCCTTATTCACTGAATGAGCAGCTTTGTTACGTTGATTTGGGTAATGAATATCCGGTTCTTGTCAAGATTA<br>CTCTTGATGAAGGTCAGCCAGCCTATGCGCCTGGTCTGTACACCGTTCATCTGTCCTCTTTCAAAGTTGGTCAGTTCGGTTC<br>CCTTATGATTGACCGTCTGCGCCTCGTTCCGGCTAAGTAACATGGAGCAGGTCGCGGATTTCGACACAATTTATCAGGCGAT<br>GATACAAATCTCCGTTGTACTTTGTTTCGCGCTTGGTATAATCGCTGGGGGTCAAAGATGAGTGTTTTAGTGTATTCTTTTG<br>CCTCTTTCGTTTTAGGTTGGTGCCTTCGTAGTGGCATTACGTATTTTACCCGTTTAATGGAAACTTCCTCATGAAAAAGTCT<br>TTAGTCCTCAAAGCCTCTGTAGCCGTTGCTACCCTCGTTCCGATGCTGTCTTTCGCTGCTGAGGGTGACGATCCCGCAAAAG<br>CGGCCTTTAACTCCCTGCAAGCCTCAGCGACCGAATATATCGGTTATGCGTGGGCGATGGTTGTTGTCATTGTCGGCGCAAC<br>TATCGGTATCAAGCTGTTTAAGAAATTCACCTCGAAAGCAAGCTGATAAACCGATACAATTAAAGGCTCCTTTTGGAGCCTT<br>TTTTTTGGAGATTTTCAACGTGAAAAAATTATTATTCGCAATTCCTTTAGTTGTTCCTTTCTATTCTCACTCCGCTGAAACT<br>GTTGAAAGTTGTTTAGCAAAATCCCATACAGAAAATTCATTTACTAACGTCTGGAAAGACGACAAAACTTTAGATCGTTACG<br>CTAACTATGAGGGCTGTCTGTGGAATGCTACAGGCGTTGTAGTTTGTACTGGTGACGAAACTCAGTGTTACGGTACATGGGT<br>TCCTATTGGGCTTGCTATCCCTGAAAATGAGGGTGGTGGCTCTGAGGGTGGCGGTTCTGAGGGTGGCGGTTCTGAGGGTGGC<br>GGTACTAAACCTCCTGAGTACGGTGATACACCTATTCCGGGCTATACTTATATCAACCCTCTCGACGGCACTTATCCGCCTG<br>GTACTGAGCAAAACCCCGCTAATCCTAATCCTTCTCTTGAGGAGTCTCAGCCTCTTAATACTTTCATGTTTCAGAATAATAG<br>GTTCCGAAATAGGCAGGGGGCATTAACTGTTTATACGGGCACTGTTACTCAAGGCACTGACCCCGTTAAAACTTATTACCAG<br>TACACTCCTGTATCATCAAAAGCCATGTATGACGCTTACTGGAACGGTAAATTCAGAGACTGCGCTTTCCATTCTGGCTTTA<br>ATGAGGATTTATTTGTTTGTGAATATCAAGGCCAATCGTCTGACCTGCCTCAACCTCCTGTCAATGCTGGCGGCGGCTCTGG<br>TGGTGGTTCTGGTGGCGGCTCTGAGGGTGGTGGCTCTGAGGGTGGCGGTTCTGAGGGTGGCGGCTCTGAGGGAGGCGGTTCC<br>GGTGGTGGCTCTGGTTCCGGTGATTTTGATTATGAAAAGATGGCAAACGCTAATAAGGGGGCTATGACCGAAAATGCCGATG<br>AAAACGCGCTACAGTCTGACGCTAAAGGCAAACTTGATTCTGTCGCTACTGATTACGGTGCTGCTATCGATGGTTTCATTGG<br>TGACGTTTCCGGCCTTGCTAATGGTAATGGTGCTACTGGTGATTTTGCTGGCTCTAATTCCCAAATGGCTCAAGTCGGTGAC<br>GGTGATAATTCACCTTTAATGAATAATTTCCGTCAATATTTACCTTCCCTCCCTCAATCGGTTGAATGTCGCCCTTTTGTCT<br>TTGGCGCTGGTAAACCATATGAATTTTCTATTGATTGTGACAAAATAAACTTATTCCGTGGTGTCTTTGCGTTTCTTTTATA<br>TGTTGCCACCTTTATGTATGTATTTTCTACGTTTGCTAACATACTGCGTAATAAGGAGTCTTAATCATGCCAGTTCTTTTGG<br>GTATTCCGTTATTATTGCGTTTCCTCGGTTTCCTTCTGGTAACTTTGTTCGGCTATCTGCTTACTTTTCTTAAAAAGGGCTT<br>CGGTAAGATAGCTATTGCTATTTCATTGTTTCTTGCTCTTATTATTGGGCTTAACTCAATTCTTGTGGGTTATCTCTCTGAT<br>ATTAGCGCTCAATTACCCTCTGACTTTGTTCAGGGTGTTCAGTTAATTCTCCCGTCTAATGCGCTTCCCTGTTTTTATGTTA<br>TTCTCTCTGTAAAGGCTGCTATTTTCATTTTTGACGTTAAACAAAAAATCGTTTCTTATTTGGATTGGGATAAATAATATGG<br>CTGTTTATTTTGTAACTGGCAAATTAGGCTCTGGAAAGACGCTCGTTAGCGTTGGTAAGATTCAGGATAAAATTGTAGCTGG<br>GTGCAAAATAGCAACTAATCTTGATTTAAGGCTTCAAAACCTCCCGCAAGTCGGGAGGTTCGCTAAAACGCCTCGCGTTCTT<br>AGAATACCGGATAAGCCTTCTATATCTGATTTGCTTGCTATTGGGCGCGGTAATGATTCCTACGATGAAAATAAAAACGGCT<br>TGCTTGTTCTCGATGAGTGCGGTACTTGGTTTAATACCCGTTCTTGGAATGATAAGGAAAGACAGCCGATTATTGATTGGTT<br>TCTACATGCTCGTAAATTAGGATGGGATATTATTTTTCTTGTTCAGGACTTATCTATTGTTGATAAACAGGCGCGTTCTGCA<br>TTAGCTGAACATGTTGTTTATTGTCGTCGTCTGGACAGAATTACTTTACCTTTTGTCGGTACTTTATATTCTCTTATTACTG<br>GCTCGAAAATGCCTCTGCCTAAATTACATGTTGGCGTTGTTAAATATGGCGATTCTCAATTAAGCCCTACTGTTGAGCGTTG<br>GCTTTATACTGGTAAGAATTTGTATAACGCATATGATACTAAACAGGCTTTTTCTAGTAATTATGATTCCGGTGTTTATTCT<br>TATTTAACGCCTTATTTATCACACGGTCGGTATTTCAAACCATTAAATTTAGGTCAGAAGATGAAATTAACTAAAATATATT<br>TGAAAAAGTTTTCTCGCGTTCTTTGTCTTGCGATTGGATTTGCATCAGCATTTACATATAGTTATATAACCCAACCTAAGCC<br>GGAGGTTAAAAAGGTAGTCTCTCAGACCTATGATTTTGATAAATTCACTATTGACTCTTCTCAGCGTCTTAATCTAAGCTAT<br>CGCTATGTTTTCAAGGATTCTAAGGGAAAATTAATTAATAGCGACGATTTACAGAAGCAAGGTTATTCACTCACATATATTG

	ATTTATGTACTGTTTCCATTAAAAAAGGTAATTCAAATGAAATTGTTAAATGTAATTAATTTTGTTTTCTTGATGTTTGTTT<br>CATCATCTTCTTTTGCTCAGGTAATTGAAATGAATAATTCGCCTCTGCGCGATTTTGTAACTTGGTATTCAAAGCAATCAGG<br>CGAATCCGTTATTGTTTCTCCCGATGTAAAAGGTACTGTTACTGTATATTCATCTGACGTTAAACCTGAAAATCTACGCAAT<br>TTCTTTATTTCTGTTTTACGTGCAAATAATTTTGATATGGTAGGTTCTAACCCTTCCATTATTCAGAAGTATAATCCAAACA<br>ATCAGGATTATATTGATGAATTGCCATCATCTGATAATCAGGAATATGATGATAATTCCGCTCCTTCTGGTGGTTTCTTTGT<br>TCCGCAAAATGATAATGTTACTCAAACTTTTAAAATTAATAACGTTCGGGCAAAGGATTTAATACGAGTTGTCGAATTGTTT<br>GTAAAGTCTAATACTTCTAAATCCTCAAATGTATTATCTATTGACGGCTCTAATCTATTAGTTGTTAGTGCTCCTAAAGATA<br>TTTTAGATAACCTTCCTCAATTCCTTTCAACTGTTGATTTGCCAACTGACCAGATATTGATTGAGGGTTTGATATTTGAGGT<br>TCAGCAAGGTGATGCTTTAGATTTTTCATTTGCTGCTGGCTCTCAGCGTGGCACTGTTGCAGGCGGTGTTAATACTGACCGC<br>CTCACCTCTGTTTTATCTTCTGCTGGTGGTTCGTTCGGTATTTTTAATGGCGATGTTTTAGGGCTATCAGTTCGCGCATTAA<br>AGACTAATAGCCATTCAAAAATATTGTCTGTGCCACGTATTCTTACGCTTTCAGGTCAGAAGGGTTCTATCTCTGTTGGCCA<br>GAATGTCCCTTTTATTACTGGTCGTGTGACTGGTGAATCTGCCAATGTAAATAATCCATTTCAGACGATTGAGCGTCAAAAT<br>GTAGGTATTTCCATGAGCGTTTTTCCTGTTGCAATGGCTGGCGGTAATATTGTTCTGGATATTACCAGCAAGGCCGATAGTT<br>TGAGTTCTTCTACTCAGGCAAGTGATGTTATTACTAATCAAAGAAGTATTGCTACAACGGTTAATTTGCGTGATGGACAGAC<br>TCTTTTACTCGGTGGCCTCACTGATTATAAAAACACTTCTCAGGATTCTGGCGTACCGTTCCTGTCTAAAATCCCTTTAATC<br>GGCCTCCTGTTTAGCTCCCGCTCTGATTCTAACGAGGAAAGCACGTTATACGTGCTCGTCAAAGCAACCATAGTACGCGCCC<br>TGTAGCGGCGCATTAAGCGCGGCGGGTGTGGTGGTTACGCGCAGCGTGACCGCTACACTTGCCAGCGCCCTAGCGCCCGCTC<br>CTTTCGCTTTCTTCCCTTCCTTTCTCGCCACGTTCGCCGGCTTTCCCCGTCAAGCTCTAAATCGGGGGCTCCCTTTAGGGTT<br>CCGATTTAGTGCTTTACGGCACCTCGACCCCAAAAAACTTGATTTGGGTGATGGTTCACGTAGTGGGCCATCGCCCTGATAG<br>ACGGTTTTTCGCCCTTTGACGTTGGAGTCCACGTTCTTTAATAGTGGACTCTTGTTCCAAACTGGAACAACACTCAACCCTA<br>TCTCGGGCTATTCTTTTGATTTATAAGGGATTTTGCCGATTTCGGAACCACCATCAAACAGGATTTTCGCCTGCTGGGGCAA<br>ACCAGCGTGGACCGCTTGCTGCAACTCTCTCAGGGCCAGGCGGTGAAGGGCAATCAGCTGTTGCCCGTCTCACTGGTGAAAA<br>GAAAAACCACCCTGGCGCCCAATACGCAAACCGCCTCTCCCCGCGCGTTGGCCGATTCATTAATGCAGCTGGCACGACAGGT<br>TTCCCGACTGGAAAGCGGGCAGTGAGCGCAACGCAATTAATGTGAGTTAGCTCACTCATTAGGCACCCCAGGCTTTACACTT<br>TATGCTTCCGGCTCGTATGTTGTGTGGAATTGTGAGCGGATAACAATTTCACACAGGAAACAGCTATGACCATGATTACGAA<br>TTCGAGCTCGGTACCCGGGGATCCTCTAGAGTCGACCTGCAGGCATGCAAGCTTGGCACTGGCCGTCGTTTTACAACGTCGT<br>GACTGGGAAAACCCTGGCGTTACCCAACTTAATCGCCTTGCAGCACATCCCCCTTTCGCCAGCTGGCGTAATAGCGAAGAGG<br>CCCGCACCGATCGCCCTTCCCAACAGTTGCGCAGCCTGAATGGCGAATGGCGCTTTGCCTGGTTTCCGGCACCAGAAGCGGT<br>GCCGGAAAGCTGGCTGGAGTGCGATCTTCCTGAGGCCGATACTGTCGTCGTCCCCTCAAACTGGCAGATGCACGGTTACGAT<br>GCGCCCATCTACACCAACGTGACCTATCCCATTACGGTCAATCCGCCGTTTGTTCCCACGGAGAATCCGACGGGTTGTTACT<br>CGCTCACATTTAATGTTGATGAAAGCTGGCTACAGGAAGGCCAGACGCGAATTATTTTTGATGGCGTTCCTATTGGTTAAAA<br>AATGAGCTGATTTAACAAAAATTTAATGCGAATTTTAACAAAATATTAACGTTTACAATTTAAATATTTGCTTATACAATCT<br>TCCTGTTTTTGGGGCTTTTCTGATTATCAACCGGGGTACATATGATTGACATGCTAGTTTTACGATTACCGTTCATCGATTC<br>TCTTGTTTGCTCCAGACTCTCAGGCAATGACCTGATAGCCTTTGTAGATCTCTCAAAAATAGCTACCCTCTCCGGCATTAAT<br>TTATCAGCTAGAACGGTTGAATATCATATTGATGGTGATTTGACTGTCTCCGGCCTTTCTCACCCTTTTGAATCTTTACCTA<br>CACATTACTCAGGCATTGCATTTAAAATATATGAGGGTTCTAAAAATTTTTATCCTTGCGTTGAAATAAAGGCTTCTCCCGC<br>AAAAGTATTACAGGGTCATAATGTTTTTGGTACAACCGATTTAGCTTTATGCTCTGAGGCTTTATTGCTTAATTTTGCTAAT<br>TCTTTGCCTTGCCTGTATGATTTATTGGATGTT
**#2**	GAGCGCAACGCAATTAATGTGCGCCCTGTAGCGGCGCATTAAGCGCGGCGGGTGTGGTGGTTACGCGCAGCGTGACCGCTAC<br>ACTTGCCAGCGCCCTAGCGCCCGCTCCTTTCGCTTTCTTCCCTTCCTTTCTCGCCACGTTCGCCGGCTTTCCCCGTCAAGCT<br>CTAAATCGGGGGCTCCCTTTAGGGTTCCGATTTAGTGCTTTACGGCACCTCGACCCCAAAAAACTTGATTAGGGTGATGGTT<br>CACGTAGTGGGCCATCGCCCTGATAGACGGTTTTTCGCCCTTTGACGTTGGAGTCCACGTTCTTTAATAGTGGACTCTTGTT<br>CCAAACTGGAACAACACTCAACCCTATCTCGGTCTATTCTTTTGATTTATAAGGGATTTTGCCGATTTCGGCCTATTGGTTA<br>AAAAATGAGCTGATTTAACAAAAATTTAACGCGAATTACAACCGGGGTACATATGATTGGGGTCTGACGCTCAGTGGAACGA<br>AAACTCACGTTAAGGGATTTTGGTCATGAGATTATCAAAAAGGATCTTCACCTAGATCCTTTTAAATTAAAAATGAAGTTTT<br>AAATCAATCTAAAGTATATATGAGTAAACTTGGTCTGACAGTTACCAATGCTTAATCAGTGAGGCACCTATCTCAGCGATCT<br>GTCTATTTCGTTCATCCATAGTTGCCTGACTCCCCGTCGTGTAGATAACTACGATACGGGAGGGCTTACCATCTGGCCCCAG<br>TGCTGCAATGATACCGCGAGACCCACGCTCACCGGCTCCAGATTTATCAGCAATAAACCAGCCAGCCGGAAGGGCCGAGCGC<br>AGAAGTGGTCCTGCAACTTTATCCGCCTCCATCCAGTCTATTAATTGTTGCCGGGAAGCTAGAGTAAGTAGTTCGCCAGTTA<br>ATAGTTTGCGCAACGTTGTTGCCATTGCTACAGGCATCGTGGTGTCACGCTCGTCGTTTGGTATGGCTTCATTCAGCTCCGG<br>TTCCCAACGATCAAGGCGAGTTACATGATCCCCCATGTTGTGCAAAAAAGCGGTTAGCTCCTTCGGTCCTCCGATCGTTGTC<br>AGAAGTAAGTTGGCCGCAGTGTTATCACTCATGGTTATGGCAGCACTGCATAATTCTCTTACTGTCATGCCATCCGTAAGAT<br>GCTTTTCTGTGACTGGTGAGTACTCAACCAAGTCATTCTGAGAATAGTGTATGCGGCGACCGAGTTGCTCTTGCCCGGCGTC<br>AATACGGGATAATACCGCGCCACATAGCAGAACTTTAAAAGTGCTCATCATTGGAAAACGTTCTTCGGGGCGAAAACTCTCA<br>AGGATCTTACCGCTGTTGAGATCCAGTTCGATGTAACCCACTCGTGCACCCAACTGATCTTCAGCATCTTTTACTTTCACCA<br>GCGTTTCTGGGTGAGCAAAAACAGGAAGGCAAAATGCCGCAAAAAAGGGAATAAGGGCGACACGGAAATGTTGAATACTCAT<br>ACTCTTCCTTTTTCAATATTATTGAAGCATTTATCAGGGTTATTGTCTCATGAGCGGATACATATTTGAATGTATTTAGAAA<br>AATAAACAAATAGGGGTTCCGCGCACATTTCCCCGAAAAGTGCCACCTGACGTCTAAGAAACCATTATTATCATGACATTAA<br>CCTATAAAAATAGGCGTATCACGAGGCCCTTTCGTCGAATTCGTCGTCGTCCCCTCAAACTCTTGGGTGGAGAGGCTATTCG

	TTTAAGGTCACATCGCATGTAATTTACTTATTCTCTGTTGTTGAGCCACCCGGGCGCCAGATTTTGTTTAAAGCTTTGTCTC<br>TTAGTTTGTATAGACAGATTCAGAGTGCAAGGTTTCGTTCGCTCGTACCTGGTTTTCCCTGGTTCTTCACAGATAGGATTTG<br>ACTTTCTACAACACTTATGCGGCTTCCTACCCGTTTGAAGGCCGATACAGGTGCTGCGCAAAATGCGGGCGAACATAGAGTA<br>TCAAAACAACGCCTTCTAATCTAGGAATATAGGGAAGATACGTATTTGCTACCATGCTTTCTGGGGTCATTAACGACCAACC<br>TCTTTTCTTTTAAAGTAGGATTGCACAATGAATGAATACACGTGGTCCGATAACTGACCAAGTAACATGGTTATCACTCGAT<br>GTCCGCCAGACGTGTGCAAACCAACCCGGGAGTTACGTCACTAATCCTTCGCTACGTCGTGAAGATATTTACTTGTGAATAT<br>CGAGGGTAATAAGATAATAGACTGTGACTAGTATTGCCAGACTGTCGCTACCTGCAACACATAACTATCCTGAGGTTACTGC<br>ATAGTACTGATTACACCCGAGTCAAAATTTCTAACTTCTAACATGTACCTAGTAACCAGCTCAATAATTATGTCAGAATATA<br>GCTCTGGGAACCCTCGGACAATTATGATACACGGTATTAATATCTTGCTTGCGTTAGCCACTTCTCATCTTTGGATACCGAT<br>TCTATTTTGCATAGCAGTTCCTTTTACACATATAAGAATTTCGCCATAGGTATGACCTACCCCAGATCGTCGATTATCTGCT<br>GGAAAATTTATTTAACACTATGTTTCTCTCCAGATGTGAGTATACACGATAAATAATACCTGGGTACCGGTTGGTGTTATTA<br>CCTTGTTTCTAAGTGCTTAATCGGCGCTTAGTGATAAGGTTGTACTAGTCGACGCGTGGCCGCCAATTATTCTTGTCATAAT<br>TTGACTTTGTTCTATATGACTATGATCTCCTGTCATCTCACCTATTGATGCCACCTTTTCAGCCTGCAG

Supplementary Table 5 Staple sequence for the DX-based square DNA origami of 84-bp edge-length. The sequence is represented by colors; unpaired nucleotides with blue, crossovers with orange, and the 14-nt seed dsDNA domain with green.

Staple ID	Length (bp)	Staple sequences
1	58	ATAACCATGAGTGTGGCTGGTTTATTGCTGCTCGGCCCTTCCGGCATAACACTGCGGC
2	58	ATACATTCAAATAGCGGTATTATCCCGTAAGTTCTGCTATGTGGCTGTATCCGCTCAT
3	58	GGTTGAGTACTCATTAGATTGATTTAAAATTTACTCATATATACTCCAGTCACAGAAA
4	54	CGCGGTTTTTTTTCCCCTATTTGTGATAAATGCTTTTTTTTTAATAATATTGAA
5	53	TAAACGAATAGCCTCTCCATTTCCAAGAGTTTGAGGGGACGACGACGAATGTG
6	53	ATCAGGGCGATGGCCCACTTTTCGTGAACCATCACCCTAATCAAGTTTAATAG
7	53	GCTCATTTTTTAACCAATATTTGCCGAAATCGGCAAAATCCCTTATAAGATAG
8	53	AGAAAGCGAAAGGAGCGGGTTTGCTAGGGCGCTGGCAAGTGTAGCGGTGTGAA
9	53	ACTGGTTTTTTTGAGGCGGATACGTGGGTCTCGTTTTTTTTGGTATCATTGCA
10	53	GTGCCTTTTTTTCTGATTAAGCATCTAGGTGAATTTTTTTTATCCTTTTTGAT
11	53	AGTAATTTTTTTATGCTGAAGAGAGCTGAATGATTTTTTTTGCCATACCAAAC
12	52	GATCGGTTTTTAGGACCGAAGTTTTCGCCCCGTTTTAAGAACGTTTTCGGTC
13	51	GCCGCTTTTTATACACTATTTGGCATGACAGTTTTTTAAGAGAATTACAAC
14	47	GTTTCTTAGACGTATACGCCTATTTTTATAGGTTAATGTTCAGACCC
15	42	AACATTTCCGTGCGTTGCGCTCAGAATAAGTAATGAGTATTC
16	42	TTCCCTTTTTTGCTACAGGGCGCACATTAATTGTCGCCCTTA
17	42	GCCTTCCTGTTACCCGCCGCGCTTAATGCGCCGCGGCATTTT
18	42	CAGAAACGCTGCACGCTGCGCGTAACCACCACTTTGCTCACC
19	42	CTTTTTTGCACCAGCGGTAAGATCCTTGAGAGGAGCTAACCG
20	42	ATCATGTAACTTTACATCGAACTGGATCTCAAAACATGGGGG
21	42	GTTGGGAACCGTCAGTTGGGTGCACGAGTGGGCGCCTTGATC
22	42	CCTGTAGCAATTTGACGGGGAAAGCCGGCGAACACCACGATG
23	42	TTGCGCAAACTAGGGAGCCCCCGATTTAGAGCGGCAACAACG
24	42	GAACTACTTACAGCACTAAATCGGAACCCTAAATTAACTGGC
25	42	CGGCAACAATTTTTGGGGTCGAGGTGCCGTAATCTAGCTTCC
26	42	GCAAGAGCAACTCCAATGATGAGCACTTTTAATTGACGCCGG
27	42	GAGCCGGTGAGAAGTTGCAGGACCACTTCTGCGATAAATCTG
28	42	CCCTCCCGTATAAAGAACGTGGACTCCAACGTAGATGGTAAG
29	42	TACACGACGGGTTGGAACAAGAGTCCACTATTCGTAGTTATC
30	42	ACTATGGATGAAGGGTTGAGTGTTGTTCCAGTGAGTCAGGCA

31	42	CAGATCGCTGAATCAAAAGAATAGACCGAGATACGAAATAGA
32	42	AATTTAAAAGGATTGGTAACTGTCAGACCAAGCTTCATTTTT
33	42	TAACGTGAGTTTGTACCCCGGTTGTAATTCGCCAAAATCCCT
34	42	TTTTCGGGGAAATTCGACGAAAGGGCCTCGTGCAGGTGGCAC
35	37	CAATCATATTCGTTCCACTGAGCGCATGATAATAATG
36	31	AAAGGAAGAGTAATTACATGCGATGTGACCT
37	31	GACGAGCGTGACGTGGCGAGAAAGGAAGGGA
38	31	GCACTGGGGCCCAAAGGGCGAAAAACCGTCT
39	31	AATCTCATGACGTTAAATTTTTGTTAAATCA
40	26	CAACTTACTTCTGATGCAGTGCTGCC
41	26	AGCATCTTACGGACTCAGAATGACTT
42	26	GAGACAATAACCCTTTATTTTTCTAA

Supplementary Table 6 Staple sequence for the 6HB-based square DNA origami of 84-bp edge-length. The sequence is represented by colors; unpaired nucleotides with blue, crossovers with orange, the 14-nt seed dsDNA domain with green, and the 4-nt dsDNA domain with red.

Staple ID	Length (bp)	Staple sequences
1	59	TGTTGTTCCAAGTTAATGCCCCCTATGGCTTTTGATGATACAGGATATAAACGTTTGGA
2	59	ATCGTCACCCAAAGGCTCCAAAAGGGTAAATATTGACGGAAATTACCAAAAATCAGCAG
3	59	TCAATAACCTACTCAGGAGGTTTACACCACCCTCAGAGCCGCCACTCACCGTGTTTAGC
4	59	CTTATTTTTTGAAAACATAGAACATTATCATTTTTTTTGCGGAACTTAATTTTCACCAG
5	59	GACAGAACGATCTGAATACACTAAAACACTCATCTCATAGTTAGCGTAATCAAGTTTGC
6	58	TAGCGAGAGGCTTTTTTAAGAAGTTTTGCCAGAGATACATAACGTTTTTTTTCAAAAG
7	58	ATTCTAAGAATTTTGCGTTTTCCCCCTCTTTTTTAAACGATTATTTTTGGCAGATTCC
8	58	ATTTAACAATTTCTTTTATTACCTTTTTTAATGGATCAATATAATTTTTTTTCCTGAT
9	57	GGGTAATTGTTTTTAATATCAGAGAATAAGAGCAAGATTTTTTTATGAAATAGCAAT
10	56	ACTTTACACATTTGGGAGGCCGATTATTTTTAGGGATTTTAGTGAAATGAGTTCAG
11	56	AATAGATGACCAGGTAATAAAACGAATTTTTTAACGGAACAAATTGAATAGCGAAC
12	55	CTCAACCCAGTACAAACTACGGCATTTCCCTCGTTTACCAGAGAGCAACAAAGTA
13	55	GATAAAAGCCAGCAGCAAATAAGGTGGGAGGGTTGATATAACGCCAGCATAACAT
14	55	CGCTGCGCAGGCGATAGCGGGGTTTTGCCTTGATACCCTAAAACATCGCGTCAGT
15	55	TTTGTTGGGAAGCGCATTAGAACGGCTAATTGCGAATAATACCGTCACTCCTTAT
16	55	AGTAGGGGAAGTTTAACAGTTTCAGCGGTCACCAGAATAAACAGCCATAAAAATA
17	55	ACGAGTAAAGTTTCAGCCCAATAGGAACTTCATAATTCAAAGCGAACCACTTTTG
18	55	TAGGTTGTGAGAAGAGCAAATCAGATATAGAACGCAAATATATTTTAGTCCCGAA
19	55	CTCCTTATAAGCAGAACAGAAATAAAGACGCGCAGGTCACAATCAATAGAGTTGC
20	54	TGACCTTTTTAATGGTTGGACAGATTTGTGTACATAGAGCCTTTAGATAATAAA
21	53	GGGAAGAAATTTTTTAGGAGCGGGCGCTAGGGCGCTGGCGGTGGTTCCTCAAG
22	53	AATGCAGGGGGTAACCCAGCTACAATTTTATCCTGAATTTTTTTCAACGCTAA
23	53	AGTAAAACCGCCAGGAAGCAAAGCGGATTGCATCAAAATTTTTTAAGAGGAAG
24	53	TAGTATCATTTTTTTTTATACAAATTCTTACCAGTATAAAAATACGGGATTTT
25	53	AACAAAGCTTTTTTTTTCAGTGAATAAGGCTTGCCCTGATTGATTCCCCTCAT
26	53	CCCAATAGATAACCATATCCCATCCTTTTTTTTTAATTTACGAGAAAGTCAGA
27	52	AGGTGTACAGAACCGAAGAACTCAAACTATCGGCCTTGCTTTTTATCCAGAA
28	51	CAGACGTTAACCTAAAACGATTTTGCAAAAAAAGTTTTGTTTTTGTCTTTC
29	51	CCTCAGAATTTAGTTTGACCTTTTATACATCACCCTCAGTTTTTCCGCCAC
30	51	TCAAACCCTTTTTATATCTGGTCAGTTGGCAAATCAATACCCAACTTATGC
31	51	AAACATGAATAAATCAAAAGTTTTCCCGAGTCGGAACCTTTTTTTATTCTG

32	51	AGCTTGCTAGGCTTGCAGGGTTTTAAGGCCTAATTGTATTTTTTGTTTATC
33	50	CGTTATTTATATAACCTTTTTCGCCTGTTTATCAACAATAGCAAGTCAAT
34	50	AAAATCTTTCATTAGTATTAAACCAAGTACCGCACTCATTTTTACAAGCA
35	50	CATAGTAACGACGAAGCGCGTTTTCATCAACGCCTCAAGCGCGACTGTAG
36	50	GCAGCCTAATTGAGAGTTACAATAGCACCATTACCAAACTTTCCCATTAA
37	50	ATTAACTAACCACCACTGATAGTTCACAAACAAATATTAGGATAAATCCT
38	50	ATAAGAAAATTGGGCTTCGAGCTCAAAATCACCGGAGATAGCAATTCCAT
39	50	GAGCCGCGTATAGCGGGCGCGAGCTGAAGAAAAATTTTGATTAGGCAGAA
40	50	ATTATCAATTTTTTGGAACGAGGGTAGCACGGGAGTCGGCTGTCCGATTT
41	50	GCCGACACGCAGTATTTATTTTAGGCGAATTATTCACACGTAAATAGCCG
42	49	TGATTGCTTAACGTCAGTTTTTTTGAATATACAGTAACAGTCAGTTAAG
43	48	CCGCCACCCTCAGTTTTCCACCCTCAGAGCGTACCGCTTCGCAAATGG
44	48	TCTGAATTTACCGTTTTGTAAGCGTCATACGCCTATTATAGGGTTGAG
45	48	AGGGCGACATTCATTTTTTGAGGGAGGGAAGAGCCTTGCTTTTGCGGG
46	48	AGAATAACATGACTAAAATAGAAAGGAACAATGGGAATTCCCAATCCA
47	48	TGCGGATGGCCAACTAACCGTAACACTGAGTCTTATTAAGCAAACTCC
48	48	AACAGTGCCAATAGTAGCAGGCGGATAAGTGGAGGCAGAATACCGAAC
49	47	GGCGAACATCAAGTGATGATGGCAATTCAAACAGTCCTGAAAGCGTA
50	47	CAACGGAATTATACGTAGCATTCCACAGAGACTGTTAAAAACCAAAA
51	47	AACGTCATCCACTAAGTAACAGTGCCCGGTGTACTAAAATTAATTAC
52	46	GCTCCATGTTATAGAAAAGGCATTTTCGAGCCTTTTGCATAGTAAA
53	46	CCATCACCCAAGTGGCGATGGTTGCTTTGACGCTTCTGAACATAAA
54	46	AGCTATCTTACCACGCATAGCTTGATACCGATAAAATTCCCAAGTT
55	45	ACAAGAGAAGGGCGTGGATTATACTTCTAGAAAACGGTAATAAGT
56	45	GCTTTCCCTGGCCATGAATAACCTTGCTGGAATTAAAAGCACTAA
57	45	CAATATTAGAGTCTGTCCTTTTTTTTTCACGCAAATCCTCAAATA
58	45	CTTTAGCGTCACAGCCCTTTGACCCCCAGCGGATTTGTTACGAGG
59	44	ACCATCGCCCGAAGCCTAGATTTTCAGGTTTGAATAATATGGT
60	44	GAATTTTCCAATGAAACCATCGATAGCAGCACCTTTTCAGTAGC
61	42	ATTCCTGATGTGAGACAGAGATAGAACCAGCACGTGAAAGCC
62	42	GAACAAGGTAGGAAAATCATAGGTACCTTTTACATTTTTTAT
63	42	AACGTCACTGTATGTAATGCCACTACGAAGGCACCAGTAAAT
64	42	CGGAACCGCCACCACCAATTCTGCGAACGAGTAGACCGCCAC
65	42	TCAATATATTATCATTTTTGGGGTCGAGTGACGGGATAACGT
66	42	CCAGAATTGAGACTCCGAAATCGGCAAAATCCCTTAAGTATT
67	42	TCACACGCTAAACAAGGATTTAGCTAAAGGGAGCCAATCAGA
68	42	AACGGAAAGGTGGCAGTCAGTGCCTTGTTAAAGAACAATAAT
69	42	TTACCAGCTTAAACAACCGATATATTCGGTCGCTGTTCGAGG
70	42	TTTCATCAAAAATACACAAGAATATCGCAAGACAAAAGTCCT
71	42	GGGAACCAATAAGGAAAGGTAAAGTAATCTTGCGGCGTCATA
72	42	CGCGCCCAATAGATAGAACGCGAGAAAATATATGTTCCGGCT
73	42	TGAGGAATGACAAGGCTCATTATACCAGCAGGTCTTCATGGA
74	42	AGAAACGATACATAATACCCAAAAGAACTTACCAGATCAAAA
75	42	AACAAAGTGGCATGAACGTAGAAAATACCAAAGACTACCTGA
76	42	CGAAAGACCTGAACCATGTAGAAACCAAAGAACGGAAGGTGA
77	42	CATTCCATCAATAAAATTAACTGAACACCAGCATCCACGTTG
78	42	CTTTGAGAAAAACATAACGTCAAAAATGTTATTTATAGAGCC
79	42	GCTAAACTTAGCAAAGCCTAATTTGCCAATCGCCAGCTCAAC
80	42	ACGGGTAAGCCAACTATTTAACAACGCCTTTCCAGGGCCGGA
81	42	CGAGCGTCAACATGTAATTTTTTTTTTAGGCAGAAGCCTGTT
82	42	GGACATTTCGTTAGCCCGATTTAGAGCTGTGCCGTTCATCAT
83	42	CTCCCGATCTGTCCCCGACCGTGTGATAGAACTGAACAGGTA
84	42	GAAAGATGCGGAATGAGGTTTTGAAGCCACCGACACGTTAAA
85	42	ATAAAGTTTAAATCTAGCGTCCAATACTTCATCAGATCATAA

86	42	TAAGAATACGGTCATTGAGATTTAGGAAGACTGGAAAGATTA
87	42	ATGTTTATACCACAGGAACGAGGCGCAGAAACACCAGAGAAT
88	42	GTTGCTAAGTAATAGGAATCATAATTACCTTAGCCTTCAACT
89	42	TTATTTGTTTCAATACCACGGAATAAGTGTTAGCAATTAAGA
90	42	GAATATAATGAACACTATCATGGATTAGAGAGTTTAATTGCT
91	42	AAATATGTTAGAGCACCTTTAATTGCTCGACCGGAGCGTTTG
92	42	TTTCAGGACCAGAGATATCGCGTTTTAATTGAGATCACCAGA
93	42	ATAACAGCGAGAAAGGTTTAATTTCAACACTTCAACCACCAC
94	42	CCCGAAAGTTTAATCATTTTTTTTGTGAATTACATCAACGT
95	42	AGAAAAAGAATGACGACGCTCAATCGTCACAGGAAAACAACT
96	42	AAAACGATCTACGTCGCATAGGCTGGCTGAGCACTCGGTACG
97	42	CCAGAATACATTTTCATAAATCAAAAATTCAGGACATCAAGA
98	42	AATACCTCCTGAGATAAAATATCTTTAGGACCTTCGTTGGGA
99	42	GTAATCTGGTTATCAGTGTTTTTATAATAAAACGCTTACCCT
100	42	GATTTTAATAGTCACCATTGCAACAGGACAGTGAGAAGGAAT
101	42	GACTATTAGAACTGAACCGGATATTCATCAGTTGAGCCACCG
102	42	TATATTTTGCTGAATAACCGTTGTAGCACTGAGTAACCACCA
103	42	CACTTGCATACTTCCTAAAGCATCACCTTCATTTGCCGGAAT
104	42	TCTACTACGCTGAGACAGAGGTGAGGCGCATTAAAGTCAGAC
105	42	AGAAGGAAATCCTCCTTTAATGCGCGAACACCCGCGCGGTCA
106	42	GTTTGATAAGTGTACGCGCTTAATGCGCTATTAGTATTAAAG
107	42	TGAATGGCCGCTACAGGTTTTTTTTCGCGTACTAGAAAGGAA
108	42	GCGGGAGACCAGTAATCGTCGCTATTAAAAAGAAAGTATTAG
109	41	CAATTTTTACTCGTATTAAATCCTTTGTAATTTCTGTTCAG
110	41	AATATTCCATTATTCCAACTTTGAAAGATGAAATAAGACGA
111	40	CTAATGCAGAAGGCTTAGATTAAGACGCGGTAATTTTAAA
112	40	ACAAAATAATTGCGCTTTTTAAGAAAAGTTAATGACAACA
113	40	TGCAAATCCATGTGAGAGACTACCTTTTATCAATCATTAC
114	40	GAGGAAACGCGTGGAAGGGTTAGAACAAGATGAATATAAA
115	39	AATAAGAAATTCGACTTGAGCCATTCTAAAGGACAGAGG
116	39	AACAGGTCAAATCGGTCATAGCCCCTTCGTCAATGTTTT
117	39	GAACCACCATAATTGACAGGAGGTTCCGTCGACATCAAT
118	39	AGCAAAAAGTGAGAGACTTTTTCATGAGCTTTTACAGAG
119	39	CCATCTTCCATGTAAGTACGGTGTCTGGGTGGTCATTTT
120	39	GATTGGCTCAGTACTAGCATTGCCCCAGCGACCGCCTGC
121	39	GAATATCATCGCCTGATAAATTTTTTAAATCCGCGACCT
122	39	ATCGGAACCAACCACCAGAAGGAGCTCTGTAAATAAAAG
123	39	TGTTAAAAACCGTCTATCAGGGTTTTCCCACTACGTGAA
124	39	TTTAACGGGACTGAAACAAACATCAGAATAATGGACTCC
125	33	AGCCGCGGGAGAAACAATTTTTTTGGATTCGCC
126	27	AGAATACGTGGCTTTTTTCAATATTTT
127	26	CGACAATTTTTTAAACAACAATCTTC
128	25	AGTTTGAGTCGATAGCTTATCCGGT
129	23	GCAAAAGCTACCATAAGGAAACC
130	23	AGTGAATTTTAACCAAATGCTGA
131	20	CCTCAGAGCCTCCCTCAGAG
132	20	AAGAGGCGGAAAGCGCAGTC
133	20	TGAATTTCGCCAAAGACAAA

Supplementary Table 7 Staple sequence for the DX-based pentagonal DNA origami of 84-bp edge-length. The sequence is represented by colors; unpaired nucleotides with blue, crossovers with orange, and the 14-nt seed dsDNA domain with green.

Staple ID	Length (bp)	Staple sequences
1	58	GAAGATCAGTTGGTGCGCTCGGCCCTTCCGTTGCAGGACCACTTCGTGCACGAGTGGG
2	58	TATTCCCTTTTTTCATTGGTAACTGTCAGTGCCTCACTGATTAAGGCGGCATTTTGCC
3	58	AGACAAAGCTTTAATAACCCTGATAAATGATCCGCTCATGAGACAAACAAAATCTGGC
4	58	ATCCTTTTTGATATGTGAAGAACCAGGGAAAAGTCAAATCCTATCATCTCATGACCAA
5	58	GCCCCGAAGAACGGGGGGATCATGTAACTCTTTTTTGCACAACATTTTTCCAATGATG
6	54	CAACAGTTTTTTCGGTAAGATCTTCTGCTATGTTTTTTTGGCGCGGTATGAACC
7	54	TGCACTTTTTTTTTGAATCTGTCAACAACAGAGATTTTTTTAGTAAATTACATG
8	53	AACTATTTTTTTATGAACGAAATTTAGATTGATTTTTTTTAAAACTTCATTTT
9	53	TACTTTTTTTTTACAACGATCGGAGCTGAATGATTTTTTTCCATACCAAACGA
10	53	TCCCGTTTTTTTACAATTAATATAAATCTGGAGTTTTTTTGGTGAGCGTGGGT
11	53	TACGCTTTTTTTTTTTTATAGGTGGTTGAGTACTTTTTTTACCAGTCACAGAA
12	53	ATGAGTTTTTTATTCAACATTTCACCCAGAAATTTTTTCGCTGGTGAAGATCT
13	50	CGCCCGCATTTTGCGCAGTTTTGTATCGGCCTTCAAACGGGTAGAACCT
14	50	TAGGGCGCTGGCAAGTGTTTTTGTCACGCTGCGCGTAACCACCACCGTGA
15	50	CGTCAGACCCCAATCATATTTTCCCCGGTTGTAATTCGCGTTAAACAGGC
16	50	GGGTTGAGTGTTGTTCCATTTTGGAACAAGAGTCCACTATTAAAGTAGCT
17	50	TTTTGGGGTCGAGGTGCCTTTTAGCACTAAATCGGAACCCTAAAGCAACT
18	42	GCCTCTCCACCGCGTTGCGCTCTAGAAGGCGTTAAACGAATA
19	42	AGGGGACGACGGCTACAGGGCGCACATTAATTCAAGAGTTTG
20	42	CGAAAGGGCCTACCCGCCGCGCTTAATGCGCCACGAATTCGA
21	42	TGACGCCGGGCCTTTTCGGGGAAATGTGCGCGTATCCCGTAT
22	42	CGGTCGCCGCATTTCTTAGACGTCAGGTGGCAAAGAGCAACT
23	42	TCAGAATGACTTTAATGTCATGATAATAATGGTACACTATTC
24	42	ACAGTAAGAGATGGCGAGAAAGGAAGGGAAGAGGATGGCATG
25	42	GCTGCCATAACGACGGGGAAAGCCGGCGAACGATTATGCAGT
26	42	AACACTGCGGCGGAGCCCCCGATTTAGAGCTTCATGAGTGAT
27	42	GTTGGGAACCGGAGGACCGAAGGAGCTAACCGCGCCTTGATC
28	42	TGTAGCAATGGGGCGATGGCCCACTACGTGAACCACGATGCC
29	42	GCGCAAACTATGGGCGAAAAACCGTCTATCAGCAACAACGTT
30	42	ACTACTTACTCAACGTGGACTCCAACGTCAAATAACTGGCGA
31	42	TTTATTGCTGAGACTGGATGGAGGCGGATAAAGGCTGGCTGG
32	42	CGAGCGAACGAGAAGCCGCATAAGTGTTGTAGAAACCAGGTA
33	42	TGGGGCCAGATCGGCAAAATCCCTTATAAATCATTGCAGCAC
34	42	CCCGTATCGTATTTTAACCAATAGGCCGAAATGGTAAGCCCT
35	42	CGACGGGGAGTTTTTTGTTAAATCAGCTCATTGTTATCTACA
36	42	ATTGAAAAAGGTCTAAATACATTCAAATATGTCTTCAATAAT
37	42	CTCATATATACTAGACAGATCGCTGAGATAGGACCAAGTTTA
38	30	CGATGTGACCTTGTTTTGATACTCTATGTT
39	30	AAGCATCTTACAAGCGAAAGGAGCGGGCGC
40	30	CGAGCGTGACACCATCACCCTAATCAAGTT
41	30	CTCGCGGTATCAAAAGAATAGACCGAGATA
42	30	TAATTTAAAAGGAGTTTTCGTTCCACTGAG
43	27	CCTATTTTTTTTGTTTATTTTAAGAGT
44	26	AGCACTTTTAAAGCTTGAGAGTTTTC
45	26	TTACATCGAACTGAGTAAAAGATGCT
46	26	TTCCTGTTTTTGCTCCGTGTCGCCCT
47	26	AATCCCTTAACGTGATCTAGGTGAAG

48	26	GCCCGGGTGGCTCTATACAAACTAAG

Supplementary Table 8 Staple sequence for the 6HB-based pentagonal DNA origami of 84-bp edge-length. The sequence is represented by colors; unpaired nucleotides with blue, crossovers with orange, the 14-nt seed dsDNA domain with green, and the 4-nt dsDNA domain with red.

Staple ID	Length (bp)	Staple sequences
1	59	GCTAATGACCACCCGCCGCCAGCATTGACAGGAGGCCACCCTCAGAGCCCAGAACGCGC
2	59	TCAGAAAATACATATCATTACCCAAATCATATAAGAACGATCTAAAGTTTTTTTTTGTC
3	59	AAATCAAGCCAGCTGTATAACGTGCTTTCAGTGAAGAATACCACATTTTTTTTAACTAA
4	57	GATTTTAAGCTAAACTTTCCAGTCGGTTTTTTAAACCTGTCGTAAGAATAGTCTTTA
5	55	ATGCTGAAAATCATTTCCTTATCATTCCTTTCGGTGTAAGCGTCATACAATCATA
6	55	TGCATGTTGCGTTGCGCTCAACAAGAGTGAGGCGGTCAGTATTAACCGTTAAAGG
7	55	AGTTGCAGCAAGTTTTTTCCACGCTGGTTTGCGTGGTTTTTCTTTTTTTTTCACC
8	55	ACAGTTGTAAAGCAACAATTTCATTTGAGTTTAACAGATAATACATTTGGTTTTT
9	55	TAGTAAAATGTTTTTTTACTGGATAGCGTCCACCAGACGACGTTTTTTTAAAAAC
10	55	CGGATGGGCAAACTGCCTAATTTGCCAGATCAATACAGTTGATTCCCAAAAGAAC
11	55	TTTAATGCGCGATTTTTGATAGCCCTAAAACAAAACTATCGGTTTTTTTTTGCTG
12	55	CCAAAAAAAAGGTTTTTCAAAAGGAGCCTTTACGGAGTGAGATTTTTTTAGAAAG
13	55	GAAGGCATTTGAGGTCTAAGAACGCGAGGAGCCAGGATTTGTATCATCGATTTTC
14	55	GAAAGCCGGGTCGAACGGGAGAATTAACAATAATATAGCATTGCTTCTGTTGTTA
15	54	AAACAGTACATTTTTTTATCAATATACAATGACAATTTTTTCCATCGCCCTGAC
16	53	TCTGACCTATTTTTTAATGGTTTAATAAGAATATTTTTTTTAACACCGGAATC
17	52	GGCGTTAGAAATACAACAATAACGGATTTTTTTTCGCCTGATTGTTTCATCT
18	52	AGAACCGCTTTTTTTTCACCCTCAGAGCCACCACCCTCCCTGATACCATTTG
19	52	AAAGAAACTTTTTTTTCACCAGAAGGAGCGGAATTATCTGGCTTTGTTTTCA
20	52	ATCATGGTTTTTTTTTCATAGCTGTTTCCTGTGTGAAAGTGCCGGAACCGAG
21	52	ATTTCAATTACCTTTTGCAAAAGAAGCACGACCAGTTTTTTTTTAATAAAAG
22	52	CGCCAGGCCCAGCACCCGAAAGACTTCAAATATCGCTTTTTTTTGTTTTAAT
23	52	TAATCATTTTTTTTTTGTGAATTACCTTATGCGATTTTTTCTGCGTTTATTT
24	50	AGGGATAACCTAAAACAACGGACAAAATCACCAGTACCGGTATACTAAAG
25	50	TTAGACTTTTTTTACAAACAATTAAGTGCCGTTTTTTAGAGGGTTGAACG
26	50	GCCAACAACCACCACGAAAAAGTTGACGCTCAATCGCTGGCCAATGTTCA
27	50	ATAATCAGGAATTGACGTCAATGTCAGATGAATATAACATTTATCACCTT
28	50	TGGCTCTAGAGCTTACATATAAGAAAATTCATATGGTTCCAGACCAACAG
29	50	TTCCTGCAAATCCAACGTTCCACATAGCCCCCTTATGCTGTCTAGGTCTG
30	50	TCCGCTCGAACGTGGATAGTAGACGGAATACCCAAAGCATTAGGGTGCCG
31	48	GGACATTTCTGAAATGTTTTTTTTTATTTACATTGGCAGTCTAAATAA
32	48	CATAATTTTTTAAAAATCAGGCCCGAGATTTTTTTGTTGAGTGTCGCC
33	48	GATGGCAATGAGTCAAGGTATTAAACCAAGGTAGCGCTGATGATACAG
34	48	CACAACATATCACCCACCTGAACAAAGTCAAGAAGGAAAACCAGGCAA
35	48	TCAGGACGTTCAAAGCATAAACAGCCATATGAATAAGAACGAGTAGAT
36	48	ATAGGAACCGGTAGCAAGCGAACCTCCCGAACTTGAGAATTGTGTCGA
37	48	TAACATTTTTTCTGCTCATTCCTCGTTAGTTTTTTCAGAGCGGGGACA
38	48	GCAAATTAACACTGTTCCAGTTTGGACTGCCCGCAGGAGGCCCAAGCT
39	47	GCTAGGGTCATTTGACGCAGTATGTTAGATAGCAGTGGCCCTGAGAG
40	47	TATATTTCCTCATTATCACCGGAACCAGCCCATCCGGCGAATTATTC
41	46	ATCGAGAACAATTTTTAGCCGTTTTTCAGTAGCGACATTTCAAGTT
42	46	GTTTTGAAGCCTTTTTAATCAAGATACGGAAATTATTTTTAAAGGT

43	46	AATCCAAATAATTTTTACGATTTTTTAAAGGTGGCAATTTTAAAAG
44	46	GAGCGCTAATATTTTTGAGAGATAACTTTTTAAGAAATTTAGCAGA
45	46	CTGTTTATCAATTTTTTAGATAAGTCCTCAGAGCCGCTTTCTCAGA
46	46	TGCAATTTTTTGTGCCACGCTGAGAAGAGTCTGTCTTTTTCATCAC
47	46	GGTACCGACGCCAGGAGTAGAAGAACTCTCGCCATAACCGTCTATC
48	46	ACCGTTGAGGCTTTTTTCAGACGATTGGCCTTGATATTCACCGGAA
49	46	AACATTAAGAGGCTTTCAACAGTTTCAGATTGTATGAAAACATAGC
50	46	TGCCGTAATAATTTTTTTAACGGGGTCAGTGCCTTGAGTTAGCAGC
51	46	GAATCTGCTCCTTTTTTTACTTAGCCGGAACGAGGCGCAGAAGGTA
52	46	AAACCATTAGATTTTTATTTCGCAAATGGTCAATAACCTAGAAAAT
53	46	TAGCGCCATTCTTTTTCTGCGCAACTGTTGGGAAGGGCGATCTTAC
54	46	GGAACTTTTTTTACGCCAGAATCCTGAGAAGTAGGATTTGCGTAGA
55	46	ACCCTCAATAGGCTATAACCCTCGTTTAATACTGCCGTCACCCTCA
56	45	AGGCAGAGGCTTCTGTCCCTGAAAGCGTAAGGAAAAACTATACAA
57	45	ACCGTAATATTTTCATCGTAGGAATCATCCATCGAAACAGTGCCC
58	45	AATATTGTAGTTGCTATTTTGCACCCAGAGGGAGGGACGGTCAAT
59	45	CGAAGCCCCCACAAGAATTGAGTTAAGCAATAGCTATCGGTGCGG
60	45	CTAACTCACGTGGACAGCAGAAGATAAACTTTGATGTAAAACGAC
61	45	GAAAGTATTATCATTTTTGTTTGGATTATACAATCCTTCGGTTTA
62	45	GACCAGGCGCGAACCGCTTTCGTCACCAGTAGCGGGATGGAATCG
63	45	CACCCGCCGCTTTAATTTAATAAAACGAACTGAGGAAGGGCGAAA
64	45	AAGTTGGGTAAGCTCGAGTAAAGCCTGGGGTGGCGAAATAAAAAT
65	45	ATAATTACTACAGAGCCTCAGAGCCGCCACCAAACAACACAGAGA
66	44	GTATAAACATGCGTCACCAATGAAATACCGCGCGTTGAAAATCT
67	44	CATAAGGGAATCATTCAACCGATTGCTACAATCAGAGGGGGTAA
68	44	GCCTCTTCGGCAACAATGAAATAGCCCAATAATGGCTATTAGTC
69	42	CCCGATTCTAAATCAAAAACAGGGAAGCAGAACTGCATCAAT
70	42	TTTTCATGTAGAAATAACCTCCGAAATCGCGCAGATAATTTA
71	42	ACAATATTACCGCCAAAGCCAACCTATTACGCCAGATCCAGA
72	42	AGCAAGGAATCAGAAAGTTTCCAATAATTTTTTCACCCAATA
73	42	TAGGGCTCCAGTATAGCCATTGCAACAGAATACGTAAAAGGT
74	42	AGACAAATACCAACGTACCTTTAAAGAAGTTTTGCTTTATCC
75	42	TAAGACTATAACATGGAACCCTACCCTTCACCGCCCCTTTAC
76	42	AAAGTAAATTTTCGGAATCGCCATATTTTATGCGTGCTCATG
77	42	TTTACATATCAAGAATCAAACCCATTCACCAGTCAATGATGA
78	42	CCGCCTCCCTGAACAAGAAAAATAATATAGCCACCACAAACA
79	42	ACATACATGTTTAACGTCAAAAATGAAACAAACGTGTTTAGC
80	42	AACAAACCGGGAGACGACCGTGTAGCACTAACAACAGTACCT
81	42	AGCGGGGTTTGCCCCCATATCAAAATTACTTGCTTTGATACC
82	42	CAAGAACGGTGTATGACAGCCCTCATAGTATTCGGAAACAGT
83	42	GCTTTGAACGAGAAGATTCATCAGTTGATATTATACCCTTAT
84	42	ATTCTTATAATTGAAGCCAGTAATAAGAGAATAGATCAACAG
85	42	AATAAATTAGTTAACTTTGAATACCAAGTTACAGCTTTCAAA
86	42	CGAGCATAATCAAAAAAGCCAGAATGGAAAAACTTTTAGGTT
87	42	GGGTTATACCTTTTCCAATCAATAATCGTAGCGTTGTCTCTG
88	42	AGAGACTATAACTAACAAAGAACGCGAGAAGCGCATGCCATC
89	42	TAAAGCATAGAGCTGGAAGGGAAGAAAGAAGGTGGGCATGAT
90	42	ACAATATTTTTGAATAAGAGCAATAAAGTACCGACGGCACAG
91	42	AGAGAGACCTTATTGGGCGCGAGCTGAACGAAAGGGGGAGCC
92	42	TAAATCCTTTTGCTGTTAGTAAATGAATAACAGCTCTGTAAA
93	42	AGGGTTATATTAATAGGTGAATTTCTTATTTCTGTTAGGATT
94	42	TCGTCGCGAACCTAGAACGTTATTAATTGAAGGATATGGGAT
95	42	TTTGCTAGCTTTCGTAATTTTCCCTTAGTTCTGAATTTGAGT
96	42	TCAGCTTAACAACTGAGACTCCTCAAGATTAAAAGTAATGGA

97	42	ATTGCGAATATTACTCATCTTGTTAATGCCCCCACTAAAGGA
98	42	GCAAGCACCGGAAAACCCCCAGCGATTACTAAAACAAACGGG
99	42	TAAAATACATGAGGTATAGAAGGCTTATGCACCATCGCGAAA
100	42	ACTTTTTCGTAATGGGCAAAAGAATACATACCAAGTACCATT
101	42	TTAAAGAACATTAACCTGCAGGTCGACTCGACGTTTAGTAAT
102	42	CGGAATACGGATATACGCCAAAAGGAATATGCTTTTCGCTGA
103	42	TGTAGCAAGGGAGTTGAATCCCCCTCAATACGAGGATCTTGA
104	42	GGCTTGCTTCCACACACCGTACTCAGGAAAGAGTACATAGTA
105	42	AGAGCAATATTCATTAAAGGCCGCTTTTCAAACTATACCGCC
106	42	TCATAAACACTATCGGCTGACCTTCATCGGTTTAGCAACGCC
107	42	AACATCAGAACCACCTCCAACGTCAAAGGCCTAATATCCCCG
108	42	GCTTTTGCAAATATGTTTTAAACCGAACTGACCTAGCGAGAG
109	42	TGAATCTAGGGCGAATGCAACTAAAGTAGCTCAACTGCTCCT
110	42	TTTGATATTAGAGAGCTAACGAGCGTCTTTTACCATGGAAGT
111	42	GTCAGGAAGAGGTCAATATAATGCTGTACGGTGTCGCGCCAA
112	42	ATTAATTCAGTAACTAATAGATTAGAGCGGAAGGTCAAATCA
113	42	ACCGAACCTTGCCTGGTTTTCCCAGTCACTAGAGGGAGTGAG
114	42	CCCTGACGATTTAGTAAGGCTTGCCCTGCGAGCACGCATTAA
115	42	TGAATCGGCAAAATGTCAGAAGCAAAGCGTAGAAAACACCAG
116	42	ATTACAGGGATTGCTGGTTCCGAAATCGGCCAACGTATGGTT
117	42	AACGAGTCGCGTACCGCGGGGAGAGGCGTTGATGGATCAAAA
118	42	ATCCTGTGTTTGCGGCGCCGCTACAGGGAGTAAATCAACATT
119	42	AGATTAAAACGGAATGGGCTTGAGATGGGCTTAATTATTGGG
120	42	TATATTTCGCTGGCGACGGGCAACAGCTGATTGAAAGCGGGC
121	41	GATAGTTTCCAGACCAGTACCAGGCGGATCGACAAGTAAAA
122	40	TATCTTTAGGGAAATCAATATCTGGTCTCAAATAAACAAA
123	40	TCGGCATAAGAACGTAGTGAATTTATCATGATTATCAGAT
124	40	GTCTTGCGCCGATGTGAGTGAATAACTTTGCACCTCGTAT
125	40	TTTTCAGATTACCTGCAAATGAAAAATCAAAGTGAGGCCA
126	40	GGAATTAGCGTTTTACGGCTACAGAGGCCCAGCAAGCCCA
127	40	TGCAGACGAGAAACGCATAACCGATATTAGCGTTATAGCC
128	40	TGTCACATTACAAAGAACCAGACCGGAACTATTATACCAG
129	40	GAAACGCTGAACACAATCAAGTTTTTTGGGCACAATTCCA
130	39	GAAATACCTTCTGACAGACGACGACAATAGATGTAATTT
131	39	CTGAGAATCATCAATATTTTTTTAATCCTGATGCGGAAC
132	39	GAACATGCCTATTTCGGAACCTTTTTTATTCTGAAACAT
133	39	GAACGAGCATGTACCGTTTTTTTAACACTGAGCACCCTC
134	39	CAAAAAACTTTGAAAGAGGACATTTTTGAACGGTGTACA
135	39	TCGAGCTTGGGAAGAATTTTTTTAATCTACGTTCAACTT
136	39	AGTGAAAGTGTAGCGGTCACGCTTTTTGCGTAACCACCA
137	39	TGAACCACGAGCCGGATTTTTTTGCATAAAGTATTCGTA
138	39	GGCCAGTGCGATTGTAGCAATACTTACAGAGGTCCACTA
139	39	GTAATCTGGCGAAAGGGGGATGTTTTTTGCAAGGCGATT
140	34	GAGTGTACTGTTTAGCGTCAGACTTACCGCACTC
141	34	AATCCGCGACTATCACCGTCACCGCTTGCGGGAG
142	34	TTAGTTTGACGCAAAGACACCACGTATTTATCCC
143	34	AGCGCCATTCCGAACAAAGTTACCGAGGGTAATT
144	34	TAGAACCCTACATTCCTGTTTAGTATCAAACAAC
145	28	CAGAAATTTTTTAAAGAAATTAGAAGTA
146	27	GATAGCTTAGATTTTTTTTTTAAGACG
147	27	GCAGCGAAAGATTTTTTTTCAGCATCG
148	27	AGGGCGATGGCTTTTTTTTCCACTACG
149	26	CCGAGTAAAGCCAGCATTTTTAATGG

150	23	GCTGAACCAGTTGGTATCTAAAA
151	21	AATTTACTCGCAAGTATGTAA
152	21	CAAAGTACGAAAGACCACTAC
153	21	TTCATTCATTGCTGATTTTTG
154	21	TCTACTACGAGAAATGACGGG

Supplementary Table 9 Staple sequence for the DX-based hexagonal DNA origami of 84-bp edge-length. The sequence is represented by colors; unpaired nucleotides with blue, crossovers with orange, the 14-nt seed dsDNA domain with green, and the 4-nt dsDNA domain with red.

Staple ID	Length (bp)	Staple sequences
1	58	GATTTAAAACTTCGTGTATTCATTCATTGTCAGTTATCGGACCACATTTTTAATTTAA
2	58	TAGCAAATACGTATGGGAACCGGAGCTGAAACTCGCCTTGATCGTTCTTCCCTATATT
3	58	TATTGACGCCGGGTGAGTATTCAACATTTTGAAAAAGGAAGAGTACAAGAGCAACTCG
4	58	GTCACAGAAAAGCCGCCTATTTTTATAGGAAAGGGCCTCGTGATAATCTTACGGATGG
5	58	ACGTTGCGCAAACGCTGAGATAGGTGCCTACGAAATAGACAGATCTATTAACTGGCGA
6	58	ACACTGCGGCCAAACCTTGCACTCTGAATGGTACGAGCGAACGAACTTACTTCTGACA
7	56	CTCCCGGGTTTGCACACGTCTGGCGGACATCGAAGAGGTTGGTTTTTTTTGTTAAT
8	55	CTCATTTTTTTATAGGCCGAAATCGGCAAAATGTGGGTTACATTTTTTTGAACTG
9	55	GGCAAGTGTATCACGCTGCGCGTAACCACCACTGTAGAAAGTCTTTTTTAATCCT
10	55	AGAACGTGGATACGTCAAAGGGCGAAAAACCGTCCGCTCATGATTTTTTACAATA
11	55	TGATAATCTCTCAAAATCCCTTAACGTGAGTTTCGTAGTTATCTTTTTTACACGA
12	55	CTAAATCGGATAAAGGGAGCCCCCGATTTAGACCTCTCCACCCTTTTTTAGAGTT
13	54	GTTCTGTTTTTTCTATGTGGCGCTATTCTCAGATTTTTTATGACTTGGTGAATT
14	54	ATGCAGTTTTTTTGCTGCCATACGAAGGAGCTATTTTTCCGCTTTTTTAGCGTG
15	54	ACACCTTTTTTTCGATGCCTGTGCTTCCCGGCATTTTTTACAATTAATATTAAA
16	43	GATCTTTATTGCTGATTTTTTTAAATCTGGAGTGTTAAATCAG
17	43	ACCCTTTTGCGGCATTTTTTTTTGCCTTCCTTCCACTATTAA
18	43	ATCTCAAAGCTTTAATTTTTTTCAAAATCTGGGCTAGGGCGCT
19	43	CGGGTGTCAGACCAATTTTTTTTTTACTCATAAAGATCCTTTT
20	43	TGAGTTTCTTAGACGTTTTTTTCAGGTGGCACGCCGTAAAGCA
21	42	TTTTGCGCAGCGCGTTGCGCTCAGCGAAGGATTTCGCCCGCA
22	42	GCCTTCAAACGGCTACAGGGCGCACATTAATTACCTGTATCG
23	42	CGCATAAGTGTACCCGCCGCGCTTAATGCGCCGGTAGGAAGC
24	42	GACCGCGTTGTTTTGTTTTTTATACTCTATGTAGTGACGTAA
25	42	AAACTAAGAGAGTGAAGAACCAGGGAAAACCACTGTCTATAC
26	42	GCTCAACAACAAAGAAAGCGAAAGGAGCGGGCCGCCCGGGTG
27	42	AAATTACATGCAACGTGGCGAGAAAGGAAGGGGAGAATAAGT
28	42	TAAACGAATAGGCTTGACGGGGAAAGCCGGCGGATGTGACCT
29	42	GATAATAATGGGGGACGACGACGAATTCGACGTTAATGTCAT
30	42	AATGTGCGCGGCAAGTTTTTTGGGGTCGAGGTTTTTCGGGGA
31	42	TGTTTATTTTTTACGTGAACCATCACCCTAATAACCCCTATT
32	42	TCAAATATGTATCTATCAGGGCGATGGCCCACCTAAATACAT
33	42	CTTATTCCCTTTGATAAATGCTTCAATAATATCCGTGTCGCC
34	42	ACCCAGAAACGTGTTCCAGTTTGGAACAAGAGGTTTTTGCTC
35	42	TAAAAGATGCTGACCGAGATAGGGTTGAGTGTCTGGTGAAAG
36	42	TGGGTGCACGACCCTTATAAATCAAAAGAATAGAAGATCAGT
37	42	AGGCGGATAAAGTTTTCCAATGATGAGCACTTGACTGGATGG
38	42	CACTTCTGCGCAGAGTTTTCGCCCCGAAGAACGTTGCAGGAC

39	42	CGGCTGGCTGGTCAACAGCGGTAAGATCCTTGTCGGCCCTTC
40	42	TGGGTCTCGCGTTGTAATTCGCGTTAAATTTTCCGGTGAGCG
41	42	AGCACTGGGGCCCCCAATCATATGTACCCCGGGTATCATTGC
42	42	GCCCTCCCGTATTCGTTCCACTGAGCGTCAGACAGATGGTAA
43	42	ACCAAACGACGGCACAACATGGGGGATCATGTATGAAGCCAT
44	42	GCATTGGTAACGAGTCAGGCAACTATGGATGACACTGATTAA
45	42	CTTTAAAAGAAAGTGATAACCATGTTACTTGGTGCAATCCTA
46	26	ACGATCGGAGGACACCATGAGTGATA
47	26	CATGACAGTAAGATGAGTACTCACCA
48	26	GTCGCCGCATACACGGTATTATCCCG
49	26	ACTACTTACTCTAAGCAATGGCAACA
50	26	AAGGATCTAGGTGTATACTTTAGATT
51	26	CCTAGATTAGAAGCCAGAAAGCATGG

Supplementary Table 10 Staple sequence for the 6HB-based hexagonal DNA origami object of 84-bp edge-length. The sequence is represented by colors; unpaired nucleotides with blue, crossovers with orange, and the 14-nt seed dsDNA domain with green.

Staple ID	Length (bp)	Staple sequences
1	59	AGTATAGCCAGACAGGTAGAAAGATTAACGGAAAGCGCGAAACATTTTTTAGTACAACG
2	59	TGGTAATAACTTTCCATGACCAACTTTGAAAGAGGACATTTTTTGATGAACGGTAATAA
3	58	GCCAGCTTTGCACGACGGCCAGTGCTCATTTGTTCAGGTTTAACGTGAGGATTATGTG
4	58	CTACGATTTTTTTGGCACCAACTTCGAGGTGAATTTCTTTTTTTTAAACAGCTCGGAA
5	58	GGAATACCCACGCGACAGAATCAAGCTATTATGAGAGACTACCTTTAACAATAATGAT
6	58	CCAGCAGGCGCCCACCACACCCGCCAGTACGGCAATTACCTGAGCACTCAATCTGATG
7	58	GCAGATTCACGCAGCTACAATTTTAGGTCAGGATAACCTTGCTTCTCCGCACTCCAGT
8	57	CTCAGAAAACCGCCAACAATGAAATATTTTTTCAATAGCTATCACCACGGGTACAGA
9	57	TTCTGAATAATGAGTAACATTATCATTTTAACAAAGAAACCACATCAATAGATAAAA
10	57	AAATAAGGCGGAATCGCCATATTTAACAAAACATGTAATTTAGATTTAATATAGTAA
11	57	CCTTGATAGGTTGAGCGCCAGGGTGGTTTTTTTTTTCTTTTCAAGTCCACTGATAGC
12	57	GTCTCTGCATTAAAAACGTTAATATTTTTTTTTGTTAAAATTCCGCATCGACGTCAA
13	57	TATATGTAAACAACATGTTCAGCTAATGCCGCGCCTGTTTATCTTAACCTGGTCTTT
14	57	CACCAGACCCTCAGGTAATATCCAGATTTTTTCAATATTACCGAATGGCTTCAATAG
15	57	AGAATCCTTGTTCCTTATCATTCCAAGAATATTAAACCAAGTAGTAAATCAGCAAAC
16	57	CAAAATTAATCTAAAGCATCACCTTGCTGTCAAATATCAAACCAAAGAAGAAATATG
17	57	ATCGGGAGAAAACTAATAGATTAGAGCCGTAGATAATACATTTCAGATGAGTTTAGC
18	56	GAGATTTATCGTTGGGAAGAAAAAGACTGGATCTTCTGACCTAAGCAGAGGATAA
19	55	ATTAAGTAAGGGCGAATCCTTTGCCCGATATCAAATAGCATTCATTATGGAGAAT
20	55	AACTAAAAATTTTCTCAATCATAAGGGAGCAAAAGGTATCGGTTTATCATTTTGA
21	55	GGGAGGTATCAGATCATTACCGCGCCCAAATTACCTTTTGCGGATGGCTCACCGA
22	55	ATTACCTCGAGAAAAGGTAAAGTAATTCAAGACAATCCCCCTCAAATGCTCACCC
23	55	GCGGGCGCCGATTTGGAAGGTTATCTAATGCTTTGTGCGAACGAGTAGATAACTC
24	55	GGAAACGCGACTTGCGAGCATGTAGAAATTAAGACGCCCGAAAGACTTCCATTAG
25	55	GATAAATAAGCAAATATTTAAGTATCGCACAGACAGCCCTCGAGTAACAAGCGCA
26	54	CAATCTTTTTTATATGTACCGCTATCAGGTCATTGTTTTTTTCCTGAGAGTCTG
27	54	GTTTATTTTTTTTTTGTCACAAATTAGTCTTTTTTTTTATGCGCGAACTATTA
28	54	AAGAATTTTTTTGTGGACTCCATAACCGTGCATTTTTTTCTGCCAGTTTGCGTA
29	54	CCGTTTTTTTTGTAGCAATACCAGAATCCTGAGAATTTTTTTGTGTTTTTATAA
30	54	CGCCATTTTTTTCATTGACAGGATTCACAAACTTTTTTTAATAAATCCTAATTT

31	54	TTCCATTTTTTGTCGGGAAACATAAAGTGTAAAGCTTTTTTTCTGGGGTGCCTA
32	54	CCAGATTTTTTTCCACCACCGGCCGCCACCCTTTTTTTTAGAGCCACCAGCCGC
33	54	AGTCATTTTTTGAGGGTAATGCCTTTACAGAGAGATTTTTTTATAACATAAAAA
34	53	GCATCGGAAAGTGAATACGACGACAATAAATGCTGATAGTTCAGAAAACGAGA
35	51	GAAACCGTATGTTATTCATTACCCAAATCAACGTAATTTTTTTCAAAGCTG
36	51	AAGTGCCCCGCCACTAGGGCTTAATTGATTAAATAAGAGGCTTTTGCAAAA
37	51	AGGGAGTTTTTTTTTAAAGGCCGCTTTTGCGGGATCGTTTAAACGCAAATC
38	50	ACATTAAGGGCGCTGCCAATTCAATACCAAGTTACAGAATTGAAGAGCTT
39	50	TGATACAATTGCGAATTTAATTAATACACTAAAACACAGACGGTGTATGG
40	50	TCAGCATGCGATTTTCATTGAAAGAACGCGAGAAAACGACAAACACCAGA
41	50	CGATGAGGTAACGCCATAGTAGATTATTTGCACGTACGTATTAATCGGTG
42	50	ACGGGAACCAATGAAAGAGGAAGCTGAGAAGAGTCATAATTTAAGCCATT
43	50	GTAAAATGAAGCCTTAGGTCATTTTTTTAATGGAAATAGGAATATAGAAG
44	49	GTGGTTTTTTTTTCCGAAATCGGCAAAGGTCCACGCTTTTTTGTTTGCC
45	49	TAAGATTTTTTTCTCCTTATTACGCAGAGGAAACGCATTTTTTAATAAC
46	49	AGCGATTTTTTTGTAACAACCCGTCGGGCGTCTGGCCTTTTTTCCTGTA
47	49	AATAATTTTTTTAAGGGACATTCTGGCAATGGATTATTTTTTTACATTG
48	48	CCAATTTTTTTTAGGAACCCATGTACCACATGAAAGTTTTTTTTAAGA
49	48	ACCGTTTTTTTTTCCAGTAAGCGTCATACATGGCGCTTGCTCTAAAAC
50	48	ACGATTTTTTTTCTAAAGTTTTGTCGTGTTTTAACGGTTTTTGTCAGT
51	48	AGTTGTTTTTTTAGATTTAGGAATACCACATTCAAATAGCGAGAATAA
52	48	GCCTTATAGTTAGAGGGGACGACGACAATTGTAGCCAGAATGCTAGCT
53	47	ATTGTTTTTTTTTCGAAATCCGATCACCGTACTTTTTTTTCAGGAGG
54	46	ATACGAGGATCCCCATCAAAAATAATTCATTCTCCCAAGTTTTTTG
55	46	AGAGATCAAAGGCCCCTATTATTCTGAAGTAACACTCTGGTGCCGG
56	46	AAATGAATCCAGAGGCTCAATCGTCTGACAACAGAGGGAGGGAAGG
57	46	ACAGGAATGGTTGCGAGTTGCAGCAAGCATCCCTTGGTGAGGCGGT
58	45	GGCCAACGGCCCACACCGTAATGGGATAAGCTCATAATCATGGTC
59	45	CCTGAGTACGAACCAGATAGGGTTGAGTGATTGCCGCTTTCCTCG
60	45	AGCGCGTTTTTCGCTGAAGAGGCTTTGAGGACCGGATAGCAAACG
61	45	CAGGGAAGCGAAATATCAGCGATAGCTTAGACCAATCAATTATCA
62	45	CGAGCGTCTTAATAGCATGAGCGCTAATATCCGATTGAGATAGAA
63	45	GCGCGTACTACGGTACGCTTCTTTGATTAGTAAACAGAATAAATC
64	45	CAAGAATGACAAAAAAGCGTAAGAATACACGCTCATAAACAGCCA
65	45	ATGAGTGAGCTTTAGTTCGGATTCGCCTGATAATATCTCCTAAAG
66	45	GACTCTAGAGCCGGAAGCCTGTCGTGCCAGCCCCAAATGTGGGAA
67	45	GAGCAAACAAACCCTGTGTTAGAACCTACCAACGTTATGCGCAAC
68	45	AAAGGGTGAGTACAAAGCCGGTTGATAATCACACCGCTTGAGTTT
69	45	ACAGGAAGCACTCCACTACAACGCCTGTCAGTTAAAATATGATAT
70	43	TCAGTGAGGCTAGAGCTAACAATTTCATTTGATCCAGCAGCAA
71	42	ATGACCATAAAAAATCACCGGCTTAGTTTTTGGTTATATAAC
72	42	TTCAAAGCGAGACCGGAGTCGCTATTTTTTTAATTTTCCCTT
73	42	TGCTGTAGCTATGTTTTATGATGAAATTTTTCATCAAGAAAA
74	42	TTCGCAAATGATAACCTATATACAGTTTTTTGTACCTTTTAC
75	42	AAGCCTTTATACGCAAGTAATCCTGATTTTTTTGGATTATAC
76	42	GAAGTTTTGCGGGGGTAGGTTTGAAATTTTTGACCGTGTGAT
77	42	TTTTAGTTAATAAGGGGCTTGAGGTATCATCGCCTCATTTTC
78	42	AAAATCAAAGAAAAAGCAAAATCAAAAGAACTGGCGATAAGT
79	42	ATATATGAAGCCGTCTAAGAACGCCAGTCACACGACATCGAG
80	42	CGAATTATGGCAAAGCGAACGTGGAAAATCCTGTTAATATCT
81	42	AAATTGCTTTACAAATTACGCCACATCAACATTAATAGAAGT
82	42	CCAGCGATTACTTAACTTTCAACCGGAATAGGTGTCGACCTG
83	42	CCAACGCAATCATAACGATAAAAACCAAACTAATGGGCGGAT

84	42	AAAATTCAGCAAGATCCCTCAGATCCCAATCCAAAAGCCCAA
85	42	ACACCGGTCAACAGCCTCAGAACCGCCAAGTACCACAGATAC
86	42	CCTAAAACTTGCTGAGCCGCCACGAGCGGGAGCTACTCAAAC
87	42	AGGGCGAGTATTGGGGCAGGTCATCCTGTGTGAAAGAGAGGC
88	42	ACTATCAGTATCATATCCTGATTATCACATATATTTAGCAAC
89	42	CTCCATGTTATACCACAACATTACCAAAAAAAAGGTTGACCC
90	42	TTTGCCACAATGACATTAAACGGGTAAACATAGGCAGAAACG
91	42	CGGGCCTTGTGCTGTCCCAGTCACGACGAAGGTGGAATAAAG
92	42	AAGGATTGAGCCACCGTTATACAAATTCCCTGTTTATAACCC
93	42	ACCCTCAAGGATTAAATTACGAGGCATAGTAAGTATCAAGAG
94	42	TCGTTTACAAAAGGGCGGGGTTTTGCTCCCCTCAGTATAAAG
95	42	ATAACGCCCAGACGATTACTAGAAAAAGTTACCAGAACCGCC
96	42	GAGCCAGTAATTTCATAGCGTCCAATACGTCAGGAGGTTTAA
97	42	TTTCAACGTAAATTAGAATATAAAGTACCTTTTTCTCGTCAT
98	42	ACGAGTATTTAATCGGCTCATTATACCATGCGGAAAAATATA
99	42	TGTTGGGTGACGGTAATCGTAAAACTAGTCAGGCTTAATTTT
100	42	AGGGGGACTTCGCTACAATTCGACAACTAAACAGACATCAAT
101	42	CCAGGCGATACGTAGATAGTTGCGCCGATCTTTTCAGCCCTT
102	42	TTTAAGAATATAAATGGCTGACCTTCATAGTTTCCAACAACC
103	42	ATGAGGACAAGAGTATAAAGGTGGCAACAAAGTAAATTAGCG
104	42	ATCGCCCCCCCCTTGCAGATAGCCGAACTACATACAATCTTG
105	42	TAGAAAAAAAGTTAATTTTCGGTCATAGACGCATACTTTTTC
106	42	ACAAGAACTAAAGAACCGATATATTCGGCATCGGCCCAGAAG
107	42	CCTGAACTAGGTCTAGTCAGAAGCAAAGATCAGTACAGTAGC
108	42	ACCATTATAGAGCCATAATATCCCATCCATAGTGACATCAAA
109	42	TGGGAATCCATTAGATAGCAGCACCGTACGGATTGATTTATC
110	42	CGAGGCGCTCATCTCTCCAAAAGGAGCCTAATAATAAGGAAC
111	42	CCGTCACTCGAATTAACTGAACACCCTGAAGGTGAATAATCG
112	42	TGCCTATAGTACAAAGCCAGCTTTCCGGGAAAAGCATTTTTG
113	42	TAATAAGATATGGTAGACAATATTTTTGCCAGCCACGCCACC
114	42	CGCCAAATGAGTTATAAGAAACGATTTTTACAAAATGGAAAT
115	42	ACCTACAGACCTGAGGGCGACATTCAACAGAGAGAACGTCAA
116	42	CCCTTCTTTTTGACCCTAATTTGCCAGTTTGTTTATAACCCA
117	42	AACAAGCTGAGTGAATTAGAGAGTACCTTTGCACCAGGCGTT
118	42	TTAGCGACGGTATTTTTTATTTTCATCGCAGTACACTCCTTT
119	42	GCTTATCACCTCCCGATTAGTTGCTATTTTAATTGTAAATCA
120	42	CATCACGCAACCACGCTGAGAGAGCAAGCAATTGAGTCTGTC
121	42	CGTCACCTTCGGAAGGAGACAGTCAAATGGTAGCTCCCAAAA
122	42	TATCGGCCATCGCCCAGTTTGGAACAAGCCAGTGAGAACCAC
123	42	GAAGATCGATTGTATTAATGCCGGAGAGCACCATCTGCCCCC
124	42	ATACCGAAGAAGAAAACAGGAGGCCGATATAACGTCTTCACC
125	42	GCCTGGCTAGCCCGACCAGCAGAAGATAAATAACAATTTTAG
126	42	AAAAGAACCTGAGATTTGACGAGCACGTTAAAGGGTCACTTG
127	42	GGTCAGTTTCATTTTGTCTGGAAGTTTCCGCGTAAGAGAAAG
128	42	GAAGGGAAAAGCCGTCAACAGTTGAAAGAAATCGCATAACAG
129	42	GACGGGGAGAAAGCTAGCGGTCACGCTGATTCCATGCAGAGG
130	42	GGAGCCCCTATTGCGTTGCGCTCACTGCTCGGAACTTAGGAG
131	42	GGTTTGCAAAACCGTTGGTGTAGATGGGGCATTAACGATTGG
132	42	GGGCGATGCGCGGGTTGTTATCCGCTCAAATTCGTTTTTTAA
133	42	CCAATAGCGGATTGTACGTGAACCATCATGCATTAACACAAC
134	42	CAAACGGGAACGCCGGGTACCGAGCTCGCAATTCCATGAATC
135	42	ATTAGACGTAGATTGGGCGCGAGCTGAATTGTAAATGGCGAA
136	41	CAAAGACTTACCGAATAATCAAAATCACTGATACCATGCCA
137	40	ATCATCATATTGCACCCTCATTTTCAAGACTCCAATGCAA

138	40	TGCCTGAGAACCCTGATGATGGCAATTCCAGAAGGCAAGC
139	40	TTGAAAATCTTTTTTCAGCGGAGTGACTAAACAGCCGGAA
140	40	CAATCGCTGTCCAGAAGGCTTGCCCTGATAGCGAAAGACA
141	40	GAAAGAGACCGAACGACGTTAGTAAATGGGAGGAGTGTAC
142	39	AATGTTTATCTACGTTATTTTTTTTAAAACGAACTCATC
143	39	CTCATTCCGAGGGTAGCTTTTTTAACGGCTACGGCTTGC
144	39	TATTATTTAGCTTGCAACAGGAAAAGTGGCACTTACCAG
145	39	TTAGAATCACAGACGGGCAACAGCTGTTGTTCATTAAAA
146	39	ATAGCTGTTGAATTTTTGTTAAATCGGTCACGTCTATCA
147	39	TCAACCGTTGAAGTGCCCGTATAAAAGCATTCGCCTCAG
148	38	TAAATATTGACTTTTTTTGGAAATTATTCATTAAACAA
149	38	CAGTATTAACATTTTTTTCCGCCTGCAACAGTGATTAA
150	38	GGGTCGAGGTGTTTTTTTCCGTAAAGCACTAAACCGCT
151	38	AAACCAGGCAATTTTTTTAGCGCCATTCGCCATCATGT
152	36	ACCCTGATTTGCCTTTATTTTTTTGCGTCAGACTGT
153	36	TCCAACATCCTGAATCTTTTTTTTTACCAACGCTAA
154	36	CAACTAAGCGCTTAATGTTTTTTTCGCCGCTACAGG
155	36	TATATTTCAAGCTTGCATTTTTTTTGCCTGCAGGTC
156	36	ATTTTTAGTAATGTGTATTTTTTTGGTAAAGATTCA
157	32	ATGAAAAATTACATTTTAATTGCTGAATATAA
158	30	GCTGTCTAAAACATGCGTTTTAATTCGAGC
159	30	CACTAACACAATAATGACCATTAGATACAT
160	30	AAAAGTTTGGAAGGAATACTTTTGCGGGAG
161	29	TTTAGTAGTCGAGAGGGTTTTTTGATATA
162	23	GATTTTGGAATAGATTTTTCACG
163	21	AAATATTAAGAACTATTGTGA
164	21	AAGATTAACCATCGCAAGGCC
165	21	TGATAAGAAATCAAGACTTGC
166	21	TTGATTCGCAAGTGGAAAGGA
167	21	TCTACTAAGGGTTTCAAGGCG
168	21	GGCTGGGGATAGAGCGGAATT

Supplementary Table 11 Staple sequence for the 6HB-based hexagonal DNA origami of 63-bp edge-length. The sequence is represented by colors; unpaired nucleotides with blue, crossovers with orange, the 14-nt seed dsDNA domain with green, and the 4-nt dsDNA domain with red.

Staple ID	Length (bp)	Staple sequences
1	60	TTTTGCTAAGAGAAAGTTTATTTTGTCAAACAGCCACTCCAACAGGTCAGGGATAAGTGC
2	60	TTTCAGGACAGAAAGTATAAAGCCAACGCGCGCCCTGACCATTAGATACAATGAATATAC
3	60	AATGCAGAGATTTACATGATTAAGACTCAGCGCATGAAGCAAAGCGGATGCAAAAGGAAT
4	60	AGTTTCCAGGACTAATGGTTTAATTTCATGACAAGCTAAAACACTCATCAGAAATACGTA
5	60	AAATCACAGCCATTACGGAAATTATTCAAAATCAATAGCTCAACATGTTTTTACCATTAG
6	60	TTTTTGAGTAAGAAGAGAATATAAAGTAAATCGGCTAGCATTGCCCCCGAGCTTTAATGC
7	59	GTCAGATATCTGGTCTCAAAATGTAGGGCTTAATTGTTTCATCAAATGGTCAATAACCT
8	59	AACGCCAGACGGTCAGGTAGAACGCAGTATGTTAGCAGAATAAAAAGATTAAGAGGAA
9	59	ATTAGTGAGGCCGATGAACCCTAAGGTAAAGTAATTAGCATGTGCTTGACGGGGAAAGC
10	59	CAGGCGAACCGCCTCATTAAGATAGAAAATTCATATTAATTTGAGAGTACCTTTAATTG
11	58	ATCATAGGTGACCAATGAAACCATCTAGAGCTCAGCTACAATTTTACCGATTGCTTTT
12	58	TGAGTAGAACAAAACATCGCCATTAAAGGTGGGAACGGGTATTAAAGGCAGAGTCGGC

13	58	GGAACGAGGCTGCATCACATAAAAACAGGGACTTATTAAGATTCATCAGTTGATACAT
14	58	CGTATTAAATGCATCGGGAGAAACACCAATTCAGAAGGCTTATCCGATCATATACGTT
15	58	ATCCTCATTTTTAAGTATAGCCCGGGCTTCAAATAAGAAACGATTTAAAAGAAGAAAG
16	58	GTAACATTTTTTAGCTGCTCATTCAGTGCAGCAACACTATCATACAGGTCTCACCCTG
17	57	CCATCGCGAATAATGACTGGATAAGCGGGCGCTAGGGCTTTTTTTGCTGGCAAGTGT
18	57	CTTTACAGAGAAACGTAGAAAATACATACAGGTGGCAACATATTTTGTTTGCGTTTT
19	57	TTCCAGAGCCGGTTTACCAGCGCCAAAGAAGGGCGACATTCAATCCTGAATTTTGCG
20	57	CCTCCCGACTCACCGGAATCATAATTACTAAAGCCTGTTTAGTGTATTCTATAACAG
21	57	CCGTTTTTATAGAATCGCCATATTTAACACAACATGTAATTTACCAAGTAGGGCGCG
22	57	TCGTGAAAGACAGCTTTTTTTATCGGAACGAGGGTAAATGAATTTTTTTTTTGTATG
23	57	GATAACCCACCCGAACAAAGTTACCAGAAACCGAGGAAACGCATCAGAGGCATAAAT
24	56	GCCCAATATTTTTTTGAACCCATGTACCGTATAATTAATTTTCCTTTTTTTTAGAA
25	56	AGTTTCGTTTTTTTTACCAGTACAAACTACACCTAACAACTAATTTTTTTGATTAG
26	56	CGGGTAACGTTAGTAGCAACGGTCATTGTGAATTACCTTATGCGTTTTTTATTTTA
27	56	ACAACTTTTTAAAGGAATTGCCCACGCAACGCGCCTGCAATAGCTATCTACAACAA
28	56	GTAGCATTTTTTTTTCACAGACAGCCCTCACGAGGCCACCGAGTTTTTTTAAAGAG
29	55	AAACTAGCATTTTCGAGCCAATTCCAACATCAATTCTACTAAGCCCTAGACAATA
30	55	AGCTTGGAGTAGTAAATTGGTACCCAACGAAAGAGGCAAAAGAAGGCAATGAGGA
31	55	GCTTGCCGGAATACCCAAAAAACTGAATTACCCTGACTATTAGTAAGATTCAACT
32	55	AGAATGACGCAAAGACACCAAATCCAAAGCGAACCAGACCGGTTGATAAGCGGGG
33	55	GACTACAGGGAGGGAAGGTATTGCACCTAATTGCTGAATATAACGTCAGCCAGCA
34	55	GCCCGAGCGTTATACAAATTCAGATATTGCGAACGAGTAGACCTTTTACGTAGAT
35	54	AGAAAATAAGTTTTTTTTTTTACGGGGTCAGGAACGCGAGATTTTTTTAACTTT
36	54	TTCAATCATCATATTTTTTTTCCTGATTATCTACATTTTGATTTTTTTGCTCAA
37	54	AAGGGTTTTTTAACCGAACTCCGCGACCTGCTCCATTTTTTTTGTTACTTAGCC
38	53	TACGAGGCATATAGTCATAGACGGGAGAATTGAACTGGGGAATACCAAATAAG
39	53	CGTCGAGAGGGAAGCAAATATTATTTATCCCCGGAATAGGATTAGGAAAAGCC
40	53	CAAGGCCGGAAATGCTGGATTAGTTGCTATTAATATTGTGGGAATTACTGAGA
41	53	AGTAACAGTATTTAGTTAATAGCAAGCAAATCTTACCATAAAGAAATTCCTTT
42	53	GCGAACTGATATAGTAGTGTCTTTCCTTATCGTAATAATACGTGGCAGAACTC
43	53	ATGCCACTACGAATACAAACCGGATATTCATGCTTGAGAAGACTTTTTTTATC
44	52	TTCAGGGATAGAACCACCACCTTTTTTGAGCCGCCGCCTACTGGTCTGGCTC
45	52	GTATCGGTCCCAACCTAAAACCAAAATAGCGAGTTTTTTTAGGCTTTTGCAA
46	51	AGACTTTTAACATTGTTTGAAATACCGACCGTGTGATTTTTTTTAAATAAG
47	51	TATCTTTCATCAATAAAAACGCTCATGGCCATCACCCGCCTGCAAACGCCT
48	51	TCAGAGCGTGCCCGCAAATCCAATCGCAGAAAACACGCGTTTTCATAGCAA
49	51	AAGCCTTTTAAAGGTGAATAAGAATAAATGCGGGATGCAACTAAAGTACGG
50	51	ATAACATGTTCAGCTAAAGTCCTAAGGGAAGAAAGCGAAAGGGCTATCAAC
51	51	AAGAGTCGTTATATGCCTATTTCGGAACCTATTATTTTTTTTTCTGAAACA
52	51	ATCAAAATTTTTTTTCACCGGAACCAGAGCCACCACCGGATTAGCCAGTTA
53	51	TTGATTACAATATTATACTTCTGAATAATGGAAGGGTTTTTTTTTAGAACC
54	51	ATTACATTTTTTTTTTAACAATTTCATTTGAATTACCTTAAATAGGTTTTG
55	51	AATAGATATGCAGATAACCGATATTTTTTTTATTCGGTCGCTGAGGAAGGA
56	51	TGCTGAACTTTTTTTCTCAAATATCAAACCCTCAATCATTTCGCGTAGGAA
57	51	GAGGTTTTTTTTGAATTTCTTAAACAGTCCAAAAGGATTTTTCCTTTAATT
58	51	TCCTCGTTTTTTTTTAGAATCAGAGCGGGAGCTAAACATTTAGAAGAAACC
59	51	TTTTATATTACATTGGGATCGTCACCCTTTGCTAATTAATGCGCTAGTTAG
60	51	AAATCGTAATTTCAACCACCAGAAGGAGTCAATAGTTCATTTCAAACACTG
61	51	CGTAACGATTTTTTTTCTAAAGTTTGTCGTCTTTCCTTTGACCATCAAGA
62	51	GAATAGACTTGCAGACCAGTAATAAAAGGGACATTCTTTTTTTTGGCCAAC
63	49	CTTGCTTTTTTTTGGTAATATCCAGAAGTAATAACATTTTTTACTTGCC
64	49	ATTAATTTTTTTTTTTAAAAGTTTGAGACAAACAATTTTTTTGACAACT
65	49	TAACCTTTTTTTTCCGGCTTAGGTTGGAATAGTGAATTTTTTTATCAAA
66	49	ATTATACCTTTTTTAAGCGCGAAACAGAGCCACCTTTTTTTCCCTCATT

67	49	CCACCCTCAAAGTAGCGCATAGGCTGTTTTTTTCTGACCTTCCCCAGCG
68	49	CGCAGTTTTTTTTCTCTGAATTTACCGATATTCACAATTTTTCAAATAA
69	47	CAAAAATACCCTCGTTTTTTTTTTCCAGACGACGATAAAAAATCAAC
70	46	AACAAAGATAATAACCTGACGAGATTTTTTTACACCAGAACCTTTC
71	46	AATTGTGTACCGCCGACGATTGGCCTTGTTCCAGTCGTTAATAAAA
72	45	GGAGGTTTAGTCGAAATGACCAACTTTGAAAAAATCTAAAGCGTC
73	45	GCGACAGAATGCCATCTAGAACCGCCACCCTGCCCCCTAACTATA
74	45	TTGCGCCGAAAATCGGGGTAATAGTAAAAATCGTCGCAAGAAACA
75	45	TTGAATACCAAGAAAACATATGTGAGTGAATTTTAATGATCATTT
76	45	CAGAAGATAACTAAAGCGAAAGGAATTGAGGTTGGATTACCGCCA
77	45	AGCGGTCACGCGTATAAAACGGTACGCCAGATCACACGGGAGTTA
78	45	AAGAAGTTTTAATCCCCGAGTTAAGCCCAATTTAAGAACCGATAG
79	42	GCCCGAAAGAAAATATCAACGTCAAATTTTTAAAATAGCAGC
80	42	CTCCTTTTGAAGGTCATTCTTACCAATTTTTAACGAGCGTCT
81	42	TGTCTGGAAGATTCCATAAGAACGCGTTTTTGTTTTAGCGAA
82	42	GTTTAGCTATTCATTTGCCGCACTCATTTTTGAACAAGCAAG
83	42	CGGCGAACGTAGAAAGGGAACAAGAATTTTTAATATCCCATC
84	42	AGTTCAGAAAGAATGACGTAATTGAGTTTTTAATATCAGAGA
85	42	TCACGTTGACAATGTACCGAAGCCCTTTAATAAGAATAAATA
86	42	TTCATTGGCCAGAGTCCAAAAAAAAGGCCTTGATAAAGTAAG
87	42	TCACCAGATCCTGATGCTTTGACGAGCACTGCGCGGGAGTGA
88	42	GGTGTACCAGTCAGATGATACAGGAGTGAGCATTGAGAACCG
89	42	ATTATACAGACCAGCAACGGAGATTTGTCACCCTCACAGGAG
90	42	GTTGAGGGCTTTTGGACGTTGGGAAGAAGAGGACACCTGATA
91	42	ATACATGCAGGTCAACCCTCAGAACCGCATCATCGGATGAAC
92	42	AAGGCCGTTTCAGCTAACCACCACACCCCTATGGTGAAGTGT
93	42	TTCGGTCACTGTAGTAGCGATAGCTTAGGCTGATGTATAAAC
94	42	TGTAAATATTAAGAGCCTTTAGCGTCAGATAGCCCACCACCC
95	42	AGTTAATCAGAGCCCCTTATTAGCGTTTCAAGTTTCGCTGAG
96	42	CACCGACTTGCAGTAGCACCATTTTTAATGGATTATCACCGT
97	42	GCAAAAGCGAATTAATAATACATTTGAGCAAAGAATCTTCTG
98	42	TGCGGAAGATTTAGAAATCGCGCAGAGGAAGATGACTTCTGT
99	42	ACCTAAAAACCTTGTGAAACAAACATCAAGTTACAAAGTATT
100	42	GCGCGTAGCCGCGCACAACTTTCAACAGCTTTTGCGGCAGAT
101	42	CGCTGAGATTAACAGCAAATTAACCGTTCAACAGGATAATCC
102	42	GCCATTGGTAGCAAGTGAGGCGGTCAGTAGCCAGCTCTAAAA
103	42	TGATTGTAAGGTTAAGCAAATGAAAAATAACAGAGTACTTCT
104	40	TCCTTAGACAAATGCCTTGAGTAACACGCCACCCGGCATT
105	40	AGCCGCGGAATTAATATATTTTAGTTCGCTATTTACCTGA
106	40	TCTGTAAATACCAGATGATGGCAATTAGGAGCAAGTGCCA
107	40	GGATTCAGCAGCCTGAAATGGATTATATCAGTGCTACAGG
108	39	ATGAAATAGTTGTCCAATACTGCGGATGTTTAAATTTTT
109	39	TGAAAGTCCTCAGAGCCTTTTTTGCCACCCTCTTTCATA
110	39	GCGTTAAAAACAGTACATTTTTTTAAATCAATAAAATTA
111	39	TACCATAAGTTGGCAAATTTTTTTCAACAGTTATCACCT
112	39	AGAGATATAAAGGGATTTTTTTTTTAGACAGGCGTGCTT
113	38	CGAACTAACGGTTTTTTTAACAACATTATTACAATCAT
114	36	AATTCGAAATAGGTGTATTTTTTTTCACCGTACTCA
115	36	GATGGCTGATAGCAGCATTTTTTTCCGTAATCAGTA
116	36	TTGATTCATAACGGATTTTTTTTTCGCCTGATTGCT
117	36	AGCTGAAAAAATACCGATTTTTTTACGAACCACCAG
118	34	CAAAATACAATCAAGGCTGAGACTCCTCCAGTAC
119	34	TCATTACCTCAACATATTTGCACGTAAATTTAAC

120	34	AATCAATCCGACAATCTGACCTGAAAGCATGGCT
121	34	GTAATCTACTTTAACTACAGAGGCTTTGATTAAA
122	30	CAGATAGAAGAATTCTCAAATGCTTTAAAC
123	27	CTAATTTACGCTGTCCAGACGACGACA

Supplementary Table 12 Staple sequence for the DX-based triangular DNA origami of 128-bp edge-length without internal mesh. The sequence is represented by colors; unpaired nucleotides with blue, crossovers with orange, and the 14-nt seed dsDNA domain with green.

Staple ID	Length (bp)	Staple sequences
1	77	CAAAGGGCGAATTTCTATCAGGGCGATGGCCCACTGCTCATTTTTTTTTTTTTACCAATAGGC CGGGACTCCAACGT
2	77	GAGAAAGGAAGTTTAGCGAAAGGAGCGGGCGCTAGATCTAGGTGAATTTTTTTATCCTTTTTG ATGGCGAACGTGGC
3	77	CAGGACCACTTTTTCGGCCCTTCCGGCTGGCTGGTACTATGGATGATTTTTTTCGAAATAGAC AGCGGATAAAGTTG
4	58	GACCGAGATAGGGGGGGCCAGATGGTAAGGTATCATTGCAGCACTTTGAGTGTTGTTC
5	42	TAGGTGCCTCAGCGTTGCGCTCTGGATGGAGGATCGCTGAGA
6	42	ATTGGTAACTGGCTACAGGGCGCACATTAATTCTGATTAAGC
7	42	TTTACTCATATACCCGCCGCGCTTAATGCGCCTCAGACCAAG
8	42	TTGATTTAAAACACGCTGCGCGTAACCACCACATACTTTAGA
9	42	AATTTAAAAGGGGCGCTGGCAAGTGTAGCGGTCTTCATTTTT
10	42	CCAAAATCCCTTAGAGCTTGACGGGGAAAGCCAATCTCATGA
11	42	TTTCGTTCCACCCCTAAAGGGAGCCCCCGATTTAACGTGAGT
12	42	ACCCCAATCATCCGTAAAGCACTAAATCGGAATGAGCGTCAG
13	42	GTTGTAATTCGAAGTTTTTTGGGGTCGAGGTGATGTACCCCG
14	42	TTGTTAAATCAACGTGAACCATCACCCTAATCCGTTAAATTT
15	42	AATCCCTTATAGAGTCCACTATTAAAGAACGTAAATCGGCAA
16	42	TCGTAGTTATCCCGGTGAGCGTGGGTCTCGCGCCCTCCCGTA
17	42	GGAGTCAGGCATTATTGCTGATAAATCTGGAGTACACGACGG
18	26	CAGTTTGGAACAAAATCAAAAGAATA

Supplementary Table 13 Staple sequence for the 6HB-based triangular DNA origami of 128-bp edge-length without internal mesh. The sequence is represented by colors; unpaired nucleotides with blue, crossovers with orange, and the 14-nt seed dsDNA domain with green.

Staple ID	Length (bp)	Staple sequences
1	58	GAGTATGGACCGAATGATAATCTCATGAAGAAAGGAAGGGTTTTTTAGAAAGCGAAAG
2	58	TGAGAGTTGACGCCACCGAGATAGGGTTTCTATCAGGGCGTTTTTTTGGCCCACTACG
3	58	TTTATAGCGGCAACCACGACGGGGAGTCAAAAGGTGGCATTTTTTTAATAGGTGAGAT
4	57	GGTAAGCCCTCCCTTTAGTTATCTAAATTAATAGACTGGATTTTCGGATAAAGTTGC
5	57	GCAAAATCCCTTATTTAAAGAATAGGGGCAAGAGCAACTCTTTTCGCATACACTATT
6	57	TTTAAAAGGATCTTTTGATCCTTTTGGAGCTAACCGCTTTTTTTCAACATGGGGAT
7	52	AGCTTCCGTTAATGGACCCCAGAAAGTTTTTTATGGTAGCAAATACGCACTA
8	52	ATCGGAGAGTATTCTTAGTGACGTAATTTTTTTCCCGGGTTGGTTTGGATCT

9	51	GTAATCTTTTTTGTACTATGCAGTAACTTGTCCGAGGTTTTTTTTTCCCAG
10	48	CTAAGAGACAAATTACATTCGCCCGCATTTTGGAAGCCGTGAAGAACC
11	48	AATCGACCACACGTTCATGAGACAATAACTTGATCACTTCATTTTTAA
12	48	ATCATAACTCAGGAGTGCACGAGTGGGTTGGTTGATAGGCCGAAATCG
13	45	GACAGGAGATTTGCAGCGCCCTTCCGGCTGGAAGAGTTCCTATAT
14	45	GAGCGGGCGCATTTAAAGTTGGGAACCGGAGTATCCGCCTGGCGG
15	45	TGAACCATCATAACCAAGTACTCACCAGTCACAGTTGGTAGTTAT
16	44	GCGGCCACGTTAGAAGTAGCCTCTCCACCCCTGGTTTGTCTCGC
17	43	CATGTAACTCGCCCCTGATAAATGCTTCAATTTTAAAAAGGAA
18	43	CTCAGAATGACTTACATCGAACTGGATCTCATTTTAAGATCCT
19	43	AGGACCACTTCTCGAATTCGACGAAAGGGCCTTTACGCCTATT
20	42	TCAACAACCTGTATCACTAGGTACATGTGAGCACTTGGTGGC
21	42	CTGGCGCCCGTTCCACTATTACAGGTACGAGCGAAACAAAAT
22	42	CACATTAATAGACAAACTATTAACTGGCGTTTCTTGAAAAGA
23	42	TAACCACGGTAACTGTGACACCACGATGGGAACCCCGGACCA
24	42	TTGACGGTTTCGTTCACTGCGGCCAACTGCCCTTATCTTCAC
25	42	AGCACTAGTTGTAAAGTAAGAGAATTATGCTCACCACAGTCT
26	42	TCCTAGACGTCGACATATAGAACAAAGTAGCGTGGATTGCTG
27	42	ATGACAAAGTCAAATCTCTGAATCTGTGAGCCGGTGCAAATT
28	42	GGTTGGTGAAACAATGCGCTCCAGGCTGAGGCAACTTACTCT
29	42	AATAATGGAACTACTATGGATGAACGAAATTGCGTGGTAATA
30	42	ACACCAATTTAAAAAGACGTCAGGTGGCTTGCGCAGATCGCT
31	42	AACAACGACTTTTCTTGTGCAATCCTACCCGGTACCAGGGCG
32	42	GAGATAGGCCGCTACCAGGTATTATTTATCATTCAGGGGAAA
33	42	CGTGTATTCGTGTAGCCGCGCTTAATGCGTGCCTCCAATGGC
34	42	TGTGCGCCCTGTAGACTGATTAAGCATTCACACCCTACTCAC
35	42	ATCTGGACAGTTATCTATTTGTTTATTTGACGAGCGTCAGAC
36	42	ACCAAACTTCTAAACCATGTTACTTGGTGAGAAACCTGCGCG
37	42	CAAGTTTGGTCACGATAGTGTTAAATAAGTGATAATACATTC
38	42	ACATCGAATTTTCCCTGGCAAGTGTAGCACTCATAAAGCCAT
39	42	AAATATGCTGAATGTATACTTTAGATTGTAGGGCGAGCAGAT
40	42	ATAAGTAAAGCTTTAACGAAACCTTGCACCTATCTGCATAAG
41	42	GACGTAGGCGAAATCGGCGAACGTGGCGCCAAAATGACAACG
42	42	CCGTGTCTACTTCTCCCTTAACGTGAGTGGAAAGCTCTTATA
43	42	TGTGTAAGTAAATATTCCCTTTTTTGCGGTGATAACCACTGA
44	42	ACCATGAGCATTTTCTCGATATTCACAAAAGGAACTTAGAGC
45	42	GCGTCAGCCCCGATTGCTATGCAAAATATATTACCGCCTTCC
46	42	ATTATCTGAATCGGACCCTAAAGGGAGCACCCCAATGCCATA
47	42	TGTTTTTGCAGTGCTCATATGTACCCCGAATCGGATATCCAA
48	42	AGATGAGACTAGTCCAGAAACGCTGGTGGCATGACTTCGCGT
49	42	ACGGATGAAAGTAAGACAGTCTGGCAATAAGTGGCGCCGTAA
50	42	TAAATTTTCGAGGTTAACGCAAGCAAGAAGGTAGCAAGATGC
51	42	GTGTTGCTATTAATCAAGTTTTTTGGGGTTGTTAAGCATCTT
52	42	TGAAGATCAGAAAAATCAGCTCATTTTTCCCTAATACCGTGT
53	42	TGCTATGCCAATGATTAGAAGTTAGAAAATTCTGAAGGGCGA
54	42	GGTATCACATAGTCTAGTACAACCTTATTATCTTCTGAGGGG
55	41	AAAACCGGAGTGTTGTATTATCCCGTATTTTCGCCTCGGGT
56	41	AGCTATTTTTGACCCGAAGAACGTTTTTGGCGCGGTTCCAG
57	40	ATAAATCAAACGAAGCGTTGTTTTGATAGAAGAATAATTG
58	40	TGAGCTGGTTGGGAACGTGGACTCCACAAGAGTAAAGTTC
59	39	GGGGTTTTTTTTGGTCATACCTATGCGAAGGAAACATTT
60	39	AGCGCTTTTTTTGATTAAGCACTTACGTTAATTCATGAT
61	39	AGGGAAAACAACCTTCAAACGGGTAGCGCAGCACAGAGA

62	39	TGTTGTACTCTATGTGCGATGTGACCTTTGCTATACAAA
63	27	ACGACGAGCGCTCGACTGGGGCCAGAT
64	23	TTTGGAAACGTCAACATAATTAT

Supplementary Table 14 Staple sequence for the DX-based hexagonal DNA origami of 74-bp edge-length without internal mesh. The sequence is represented by colors; unpaired nucleotides with blue, crossovers with orange, and the 14-nt seed dsDNA domain with green.

Staple ID	Length (bp)	Staple sequences
1	78	AGCAATGGCAACTTTTTTGCGCAAACTATTGAGGCGGATAATTTAGTTGCAGGACCACTTCTG CGCCACGATGCCTGT
2	78	TCAAGTTTTTTGTTTTCGAGGTGCCGTAAAAAGCATTGGTATTTACTGTCAGACCAAGTTTAC TCACCATCACCCTAA
3	78	AACCACCACACCTTTTGCGCTTAATGCGCCAGCCGGTGAGCTTTGTGGGTCTCGCGGTATCAT TGTCACGCTGCGCGT
4	78	ACTATTAAAGAATTTTGACTCCAACGTCAAAAGGATCTAGGTTTTGAAGATCCTTTTTGATAA TCGGAACAAGAGTCC
5	78	AGGCCGAAATCGTTTTAATCCCTTATAAATCGTCAGACCCCTTTAATCATATGTACCCCGGTT GTTTTTTTAACCAAT
6	78	CGGCGAACGTGGTTTTAAAGGAAGGGAAGATCTACACGACGTTTGGGAGTCAGGCAACTATGG ATTGACGGGGAAAGC
7	47	AATAGACTGGATGAACTGGCGAACTACTTACTCTAGCTTTTGTTAAA
8	42	CCGGCTGGCTGGCGTTGCGCTCGAGCGTGACACTCGGCCCTT
9	42	GATAAATCTGGGCTACAGGGCGCACATTAATTGTTTATTGCT
10	42	GCCAGATGGTAGGGCGCTGGCAAGTGTAGCGGCAGCACTGGG
11	42	TATCGTAGTTAAAGCGAAAGGAGCGGGCGCTAAGCCCTCCCG
12	42	GACAGATCGCTGGGAGCCCCCGATTTAGAGCTGAACGAAATA
13	42	CCTCACTGATTGCACTAAATCGGAACCCTAAAGAGATAGGTG
14	42	TAGATTGATTTGGGCGATGGCCCACTACGTGAATATATACTT
15	42	TTTTAATTTAAAGGGCGAAAAACCGTCTATCAAAAACTTCAT
16	42	AATCCCTTAACGGTTGAGTGTTGTTCCAGTTTTCATGACCAA
17	42	GTTCCACTGAGCAAAAGAATAGACCGAGATAGGTGAGTTTTC
18	37	TCAGCTCAAATTCGCGTTAAATTTCCCGGCAACAATT

Supplementary Table 15 Staple sequence for the 6HB-based hexagonal DNA origami object of 74-bp edge-length without internal mesh. The sequence is represented by colors; unpaired nucleotides with blue, crossovers with orange, and the 14-nt seed dsDNA domain with green

Staple ID	Length (bp)	Staple sequences
1	55	CATTCCATAAAACGAACTAGAGCCACCATACAACGCTGAGACTCCTCAAGTCGAG
2	50	GTAAAATACGTAAATTTCATCGGCATTTTCGGTTAGCCCCCTTATTTGCC
3	49	ACCAGAATGAGAATAGTGTACTGGTAATAAGTTAACGGGGTCAGATAAA
4	49	ACGGTCAGCCCACGACCACCAGAGCCGCCGCTTATTGACAGGAGAGGTG
5	48	CGCCTCCCTTTGCCGCCACCCTCATTAGCAAGTTTGCCGGAAATGCAA
6	48	GGATGAGTACCGGCACCAACTTTTAACGAAAGAGGAAGTTTCAAACGG

7	48	ATAGCCCGGACACCCTCTTTCAGGGATAGCAAGTTTCGATTAGGATTA
8	48	ATTACAGAAAGATTTTGGGAAGATTTTTCTACGTTAACAGACAGCATA
9	48	AGCCAGAATTTAGCGCAGTCTCGCGCCAAAGATTTCAAAAGGGAATTC
10	48	TATTATTCTTTCATGAAAGTATCTGGCATGATTTTTAAGACTCAGGAA
11	46	TCATTAAGTTGAGGCTTGATACCGATAGAAAGAGGAATGCTTTAAA
12	46	GTTAGCGTAACCAGGACGCATCAGTTGAGATTTACCAGACTTATTA
13	46	TTTACCATGAATTTCGTTGAAAATCTCCAAATCAATTTTGCCAGAG
14	46	TTTCAGCGGAGCGAGTAGCATAACCCTCGTTTAAGAGCACACCACG
15	46	ATCAAGTTAGCGTTGACTTTTTCATGAGGCAAAAGCCAACAGGTCA
16	46	AAAGGAGCCTTATCTTGATGTTTAGACTGGATTGGCATCCGACATT
17	46	GCAACATTGCCTTGATGGGATTTTGCTAATTTCAAACGAGGCATAG
18	46	GCAGCGAAAGAGTACAACCGAAAGACTTCAAATTAAATACGTCACC
19	46	CAACAACCATCATCATAAGAATGACCATAAATTAGTTTGACCGACT
20	46	CCATTACCAGAACCAGGCCGCTTTTGCGAAATTGTTGCATCAAAAA
21	45	TAAGACACTATTAAATTGGGTTTTAGATGGTTTAAACAACTTCAG
22	45	GGGGAGTAAAACAAGAACCGTTTTTTCATTACCCAAAAAAAACCA
23	45	CAGTAAAACGAGGGAACCGATTTTACCAACTTTGTTGCGCCGTGA
24	45	GATTGGAAGCCGGAGATTTGTTTTATCGCCTGATGGATCGTCTCA
25	43	ACAAAGTTAGGAATCTCATTATACCAGTGATCTAATCGGAACC
26	43	ATCTTACCAGGAATTCTTTAATCATTGTGTTTCTGTAGTAACA
27	43	GAAAAGGAGCGTCCCTTCATCAAGAGTATAATTGTCTCATTAA
28	43	GTCAATAACCCCTCAACAGATGAACGGTGTAAACAGCAGGTCA
29	43	ACATGTTTATCGCGAAGCGCGAAACAAACAGCATCACCGGAAC
30	43	GGATGGCTGCAAACTAATACACTAAAACAGACTAAATGCCATC
31	42	ACCGCCTAGGAACCCTGCCACTACGAATTTAATTGCTTTAGT
32	42	CCACAAGAAATAGCGTGAATAAGGCTTGAAAGGAACATGGCT
33	42	GATTCCCGACTATTCATGTTACTTAGCCTCGGTCGCTCAGAG
34	42	ACTCAGGAGGTCTGTAGCGCGTGCTCAGTACCATATCACCGT
35	42	CTTTGAGCTCATCTGAACCAGACCGGAATAGAGCTCGACAGA
36	42	CGCAGTAGCCTATTAGTTTTGTCGTCTTGAACTGGACCACAT
37	42	ATTTTAATCCAGACAGTTAATGCCCCCTTGTTAGCTAGCCGA
38	42	TCAACTAAAGCAGAAAACGTAGAAAATATATAAACGTTAGTA
39	42	GTGCCCGCATACATTTTTTAAGAAAAGTATGCAGATTATGCG
40	42	AATGAATAATTACCTACATAACGCCAAAGAAGCCCAAAGGTG
41	42	GAACCGCATAGGTGGGCGGATAAGTGCCGAGAAGGTCACCAG
42	42	GAATAAGATACAGGAGAAAGGAACAACTCCCTGACGACGATA
43	42	TTTGATGTTTATTTTTAAGCCCAATAATACCAGACGAGAAAC
44	42	AAAACCAAATTGAGTGTCACAATCAATACGTCATATTGCGAA
45	42	AAGCTGCTTTTTCAACCGTTCCAGTAAGGAAAATTAGATAAC
46	42	TAATAATTCATTCAGAGAGGCTTTTGCAATCAGAGCATATGG
47	42	CAACCGAATAAATCATCGGTTTATCAGCGGCTGACAATACTG
48	42	ATAGGCTTTGCTTTATATTCACAAACAATTGAGGGGCGAGCT
49	42	CGGAATCTTGGGGCAGGGAAGGTAAATAGGCCTTGCGAGGTG
50	42	GACGATTTTGACGGAGCTATATTTTCATGTCATAACAGGCGC
51	42	AATTTCTTACAGACATATTCATTGAATCCCTGTTTAAATTAT
52	42	TGAGCCACAGAACCCATAACCGATATATGGAACGACAGGTCT
53	42	CCGCCACTTTGGGACGAACGAGTAGATTCAAAAATGGCGCAG
54	42	TTACCCTAATTCTGATTAGAGCCAGCAACACCACCCTGAGGC
55	42	TCCGCGAGAGTTAAGCCACCCTCAGAGCAATCACCAACAGTT
56	42	TTGCAGGCCTGCTCATAGTCAGAAGCAATCCATATAGTAGCA
57	42	AATGAAAAGCCACCGGAACGAGGGTAGCTTATACCTTTTAAT
58	42	CCAGCGAAACGGCTATCACCGGAACCAGCCATCGATAGCTCA
59	42	TCGAGCTAATGCTGTAGCAGCACCGTAAAATCAAAACAGAGG

60	42	TTTTCATTCAGTAGTAATTGCTGAATATTCAAAGCTTGACCC
61	40	ACCGATTACGCAATAATAACGGAATCAACGGAACAACATT
62	39	GCGGGGTTTTGTACCGTAACACTGAGCCCAACCCTCA
63	39	TACTATTTAGTAGCATTGCGCTAATAAAGAAGCGTAACA
64	39	CTAAATTGGTGTCTGGAAGTTTCATAGCGGATGTCGAAA
65	39	TACAAACCCCTCATAGAACCGCCACCCTACGATATAAGT
66	38	AGAAATTTCGCAAAGAAGAAACAATGTTAGCAATAGCT
67	38	AATTATTTTCACCGTCACCATTAGATTTTTCGCAAATG
68	38	TTTAGTTTCGTCAGACCTTTTGATAATTTCATTTTTGC
69	29	AGGGTTCCAAAAGAATAAGAGGCCTGTAG

Supplementary Table 16 Staple sequence for the 6HB-based square DNA origami of 105-bp edge-length without internal mesh. The sequence is represented by colors; unpaired nucleotides with blue, crossovers with orange, and the 14-nt seed dsDNA domain with green.

Staple ID	Length (bp)	Staple sequences
1	57	GTTTCCAACAGCATCATCTTTTTTTTCAAAATCACCGGTCAGACTGTTTTTAGCGCG
2	55	TAACACTGCGATTTTAAGACCCTCAGAAGAACCCAGCTGAGACTCCTCACGTCGA
3	53	GCTGTAGTCATTGACAACGGAGATTTGTTTGATACAACCACCTTTTGCCGCCG
4	53	ACATTTCTCGTTTACGGTGTACAGACCAGGAACAACTGAATTTTTTTCCAGTA
5	53	CAATAATCGGAACAAGAACGAGTAGTAACAGACAGTGCCCCCTTTTTTTCGGA
6	52	ATCAAGAGTTCAGCGGAGTGTTTTTATAGAAAGGCGCATAGGTTTGACCTTC
7	52	GAAATCCGCGAGGTGAATTTTTTTTAAACAGCATCATCGCCTTTTTTGTGTC
8	52	CCACTACGTCGTCACCCTCATTTTTGCGAAAGTTAAACGGGTTTTCGTAATG
9	52	TCAACTTTCTACAACGCCTGTTTTTCATTCCAATTGGGCTTGTTTTTTAATT
10	49	TAGTAAGAGTTTTTCACTATCATAACCCGCAAATGTTTTAACCTGTTTA
11	49	CGGATTGCATTTTTAAAAGATTAAGAGGAGACCGGTTTTACTCCAACAG
12	49	CAATACTGCTTTTTATCGTCATAAATATCTCAACATTTTAAATATGCAA
13	49	AAATCTACGTTTTTATAAAACGAACTAAAACGGAATTTTAAAGAACTGG
14	45	TATAGCCCGGTACCGCCCCTCAGAGCCACCACCCTCTTCTCAGGA
15	44	CCAGCATCACCAGTATTTTGCACCATTACCCCACCAGCGATAGT
16	44	TATACCAGTGACTCCTACATACATAAAGGTTTATTCTGTCACCA
17	44	CATGATTAACAGGACGGTGAATTACCTTATGAGTTTCGAAACAT
18	44	ACCTAGGCAACATATTTTTAAAAGAAACGCCAGTTAACCCTCAT
19	44	GCTATATTTAAAGGAAGACAAGAACCGGATTGCTAAAGGCTTTT
20	44	CTAAAGTACTTAGACTCTCCATGTTACTTAATCGGTTGGAGGTT
21	44	AGCGTTTCAACCGATTTTTTGAGGGAGGGAGCAGTCTCTAAAGG
22	44	GTCAGGATTTATAGTCCAACCTAAAACGAATTAAAGGAGCCACC
23	42	TAGCGGGGTTATTACAGAGGCATTTTCAGGGATGATTAGGAT
24	42	TTATTAGATCGGCACTTCAAAGCGAACCAAGCCCGTGAGGAA
25	42	AGTACCGTATCACCGTTTTAATTCGAGTTTTCGGTCTTGCTC
26	42	TCTACTATTTAGGACTGCTCATTCAGTGGTCTTTCGGGGTCA
27	42	TCCCAATTTTGCAATAAGGGAACCGAACAAAATCTACAAATA
28	42	TCATTTTAGAATGAATCTTTGACCCCCACACGCATAACCGCC
29	42	GGTTTAGAATAGGTAGGCGGATAAGTGCAGAGAAGAGCAAGC
30	42	TGACGAGGTAACGAAGTGCCCGTATAAAAAGACAGGAAACG

31	42	AGTTAGCAAACACCACATTATTACAGGTAAACCGACCACGGA
32	42	ATAAGTTGAGTAACTCTAAAGTTTTGTCAATAAGGTTCATCA
33	42	GTGCCTTTATTTTGTAGCATTCAGAAGGAGAAAGACTTGCCC
34	42	GTTGAGAATAGTAGTCACAATCAATAGATTTTAACCAGACGT
35	42	TCAACGTTGAATTTGTACTGGTAATAAGAAATTCACATCAAT
36	42	TAGTAAAAACAAAGATACCACATTCAACAAGGTGGTATGGTT
37	42	TACCAGCCAGGAGTTCTGTATGGGATTTATTCATTGATACAT
38	42	GATGATAGCCAAAGGGGCGCGAGCTGAATAATGCAACCCAAA
39	42	AACGCCATCATTTGACAAAAGGGCGACACATACATCAACTTT
40	42	AGAGGACAATAATACCAGAATGGAAAGCAGGTAAAATTAGAT
41	42	AATTGCGAGATGAACCAGACGACGATAATTTGACCTATTGAC
42	42	GGAAATTATTAAAGATTTTTTCACGTTGTGACCAAAATAGCG
43	42	AATCCTCATTCATTACGAGTAGATTTAGAAACCAACTTTGAA
44	42	AGAGGCTTCTGCGAAAAGGTGAATTATCTTCACAACCAAAAA
45	42	CAGACGGCCAAAAGGATTGGCCTTGATAACCGTCAAGTTGAT
46	42	AAAGGCTTCAATCAAAGAAGTTTTGCCAATATAACCCGACTT
47	42	GAGCCATGTCAGACGAGCCTTTAATTGTGCCGGAATAATAGT
48	42	GAGGCAGTTGGGAAGGAAGTTTCATTCCGAGGGGGCGAGGCG
49	42	AAAATGTGGTGTCTTTAGAGCCAGCAAAATTGACATATCAGC
50	42	CGCGAAAACAATGACACCCTCAGAGCCGATTAGCAATATAAT
51	42	TGCGCCGCAAAGTAATCCCCCTCAAATGTTGCTGAAGGCCGG
52	42	AAACGTCGAGCCACCAACAACCATCGCCGCGATTACAGTTCA
53	42	ACCCTCAACCAATGGGCTTAGAGCTTAACTTTAAATACCAAG
54	42	GAAAACGTGCGGATAAACCATCGATAGCCCCTCAGAACCGAT
55	42	ACACTAAGTCGCTGCCTCAGAGCCGCCAAGCACCGTAAGAGG
56	42	ATATTCGAACACTCCCATAAATCAAAAACTTTTGATAATCAG
57	42	TAGCGACCCGCCTCAGGCTTGCAGGGAGAGAGGCATTTACCC
58	42	ACCGGAAAGAATCAACCTTTAATTGCTCTCAGGTCAAAGAAT
59	42	TGACTATAGAGAGTAGTTTGCCTTTAGCGAACCAGCCGCTTT
60	42	TTTTCCGTTTGCCGGAACGAGGGTAGTTTTTCAAAAGACTTC
61	40	GAAAGTAAGAAAATTATTACGCAGTATGCAACTGGCTCAT
62	40	AAATATCGCGTATGAGGACTAAAGACCAACGGCAGCCCCC
63	39	CCAATAGCCGCCACACCCTCAGAACCGCTTTGATATAAG
64	29	GAGGGTAGCAAACGTTTAAGAGTGTACCG
65	24	CAACAGTTAATCTTTTACGAGGCA
66	24	TTGCTTTCGACCTGGGATAGCGTC
67	24	TGCGGGAAAGGCACAGAAGCAAAG
68	24	GTACAAAAATCATTTTGGGAAGAA

Supplementary Table 17 Staple sequence for the 6HB-based octagonal DNA origami of 57-bp edge-length without internal mesh when using Design Type 1 shown in **Supplementary Fig. 26**. The sequence is represented by colors; unpaired nucleotides with blue, crossovers with orange, and the 14-nt seed dsDNA domain with green.

Staple ID	Length (bp)	Staple sequences

1	58	CGTCATTTCTCAGCAGGAGTTAGGCCGCTTTTGTGTATCACCGTTAAACAGTTATTAC
2	56	TCATAGCCCCTTTTTTAGCGTTTGGCGCCAAAGATTTTAAGGGCGACATTATTGCT
3	56	CCACCGGAACTTTTTCCCTCAGAGAAGACACCACTTTTATAAGTTTATTTTGCTGT
4	56	GGTCAGACGATTTTCCTTGATATTGAAACGCAATTTTTAACGGAATACCCGCAAAT
5	56	CTCAGAGCCGTTTTCAGAACCACCAGTATGTTAGTTTTACGTAGAAAATATATAAC
6	56	GTACTGGTAATTTTTTTTAACGGGACTTGAGTTATTTTCCAATAATAAGAATATCA
7	56	GCGCAGTCTCTTTTTTTACCGTTCGAAGCCCTTTTTTTAGAAAAGTAAGCTCAATT
8	56	AGCGACAGAATTTTGTTTGCCTTTAAATTATTCATTTTAAGGTGAATTATAGCTTC
9	52	CATTAGTTTTGCCGGAAACGCCAGCAAAATTTTTCAGTAGCACCAACGGATT
10	51	AAGAATTTGGATATTACCCATACCGTAACACTGAGTTAGTAATCTATACCA
11	51	CATGTTTTCTTAGCCTCTGTAGGATTTTGCTAAACAACGCGACCGCCAAAA
12	51	CCTAATTTCGAAAGATTTAATTATCGGTTTATCAGCTCGAAGGCTAGACTG
13	49	GAGAGATTTTTCACAAGAAATGAAAGTACAGTACCAGGCGACGAGTAGA
14	48	CGTCGAGAGGGTACCGCCCTTGAGTAACAGTTTCGGAAGCTGAGACTC
15	46	CGCCACCCTCAAAGGCTTTGAGATGGTTTAATAGCGCTAGCAAGAA
16	46	TTGACGGAGCGTCATTGATACCGATAGTGAGGAAGTCATTGAATCC
17	46	ACAACAACCATGCTTTGATAAACAGTTCAGAATAATTCGCACCGTC
18	46	TAGCGTAACGACCAACTTTACAGGTAGAAAGAACATTTCAAAAGAA
19	46	AAACGCACCGCCACAGAAAGGAACAACTAACGGAGAACCCTCGTTT
20	46	ATAATAATTTTATTATACAAAAACCAAAATAGAATATAATGTCACA
21	46	AACCGAGCACAAACTAGCATTCCACAGAGGTGTACAACGAACTAAC
22	45	GTAATTGTTCAACTCTCATTCAGTGAATGAGCCACATACAGGAGT
23	45	CTGAAAACGTTGGGTGACCTTCATCAAGTCGTCACAGAATGGAAA
24	45	ATTAGATTTCATCAGGGAACCGAACTGATCTAAAGGGTTGAGGCA
25	45	GAAGTTTCGAGGCAATTGTGTCGAAATCCTTTCAAAGCCACCACC
26	45	ATTGCTGCGAGAGGTTTGACCCCCAGCGTTCACGTACCAGAGCCA
27	45	AGAGAGTTCCAATAACGTAATGCCACTATGCTTTCGCATTTTCGG
28	45	CGCGTTTAACGAGACAACGGCTACAGAGCGCCCACCGTAATCAGT
29	42	AGGTTTAGTTGATAAGCGGGGTTTTGCTTTAAGAGCCTATTA
30	42	TAAATCAAAGACTTTTTGGGAATTAGAGTCACCAAGCTGAGG
31	42	GGCAACATTATTGAAAGAGGATTTGATGAACCAGCCCATAGT
32	42	ACGACGACGATCAAGCGCGAATTTAAAGTACAAAGGATGCGA
33	42	CCCAAATGCTTGGACTAAAGATTTTTTTCATTGCGCCCAATG
34	42	CCCGGAATAGGCCAGAAGCAAAGGATTAGGATTTAAGTATAG
35	42	CTACTAATTTTAGTAGCATTACACCCTGTTACCTTCAAATCA
36	42	GGTCAATTTTTTGTTTAGCTATATTTTCTTAATAAAGACCAG
37	42	AGTTGATTTTTAATTCTGCGAACGAGTAATACCACGGCGCAG
38	42	AGCTCAATTTTTTTTAAATATGCAACTACTATCATATTTGTA
39	42	CCTTTTGTTTTGAGGTCATTTTTGCGGAGTTTTGCGAATACA
40	42	AAAGCGATTTTGACCGGAAGCAAACTCCTAAATATTTTCCAT
41	42	GCATCAATTTTATTAAGAGGAAGCCCGAAAAATCAACAGCAT
42	42	ACAATGATTTGATGCACCCTCATTTTCAAAAGCTGTTAATCA
43	42	ACGTAACGGGATAGCGTCATACATGGCTAATAGCATCAGAGG
44	42	TTGTGAAAACAAAGATAGCTATCTTACCCAGTAAGCAAGCCC
45	42	GTCAGGAGGTGGCAAGATAGCCGAACAATAAAGCCCAGTACA
46	42	GCGCATAACGCCTGAAATAAATCCTCATAGTTACCGCGCGAG
47	42	AACTACAGGCTGGCAAGAAAAATCTACGATTTGGGAGAAGGA

48	42	CTGGCATGACAGGATTTTGTCGTCTTTCATCATAAGTTGAGA
49	42	ACGGTCACAGACGTCCGCCGCCAGCATTGATTAAGTTTGACC
50	42	TTTAGGAGATTTAGACTCCTTATTACGCACCAGAGTAGTAAA
51	42	GGAATTACATTCCACATACATAAAGGTGCCCTCAGCAGTTTC
52	42	TCATCGCTGAGAATCCTCAGAACCGCCAGCAACATGTGTCTG
53	42	AGCGGAGCTGATAATAGTAAGAGCAACAAAGTACGATAAAAG
54	42	ATCAATACACCGGATGAAAATCTCCAAAACTCATCCTTTTGC
55	42	CTAAAACAAAAAGGTTCATAATCAAAATGAAAATTGAGCTTA
56	42	AAAAGAATGGCTTACATATGGTTTACCACCATCTTCTCCAAA
57	42	GATAGCGACCTTTACAACCGATTGAGGGTTCATCGGAGGTGA
58	42	TAAACGGAAACAGCGACTGTAGCGCGTTAGGGAAGCAGGATT
59	42	ATTTCTTGTAAAATCTGCGGAATCGTCAAACAGGTGTAAATA
60	42	ACCGACTGCAGCACGCATAACCGATATAAGGGTAGATGACCA
61	42	CGGAACGTTCGGTCTGAAACCATCGATATGAGCCACAAATAT
62	41	AATAGGACATTACCATGCGATTTTAAGCTGGCTCATTTGAC
63	41	TGAATTTGGAACGAATTCAACTAATGCATACATAACTGCTC
64	41	AGGAGCCGGCAAAACAGAGGGGTAATTAAAATGTTACCAA
65	41	CTTGCAGGCGAAAGGGTCTTTACCCTGTATTATAGTGGGAT
66	39	ATTGGGCTGCCCTGACGATTTAACACCACCACCCAGAAC
67	39	CTCAAGAGAAGTGCCCCCTGCCTATGCCCGTAACTCAGG
68	39	TTCTGAAGTCAGTGCACCCTCAGAACCGGAGATAAGTGC

Supplementary Table 18 Staple sequence for the 6HB-based octagonal DNA origami of 57-bp edge-length without internal mesh when using Design Type 2 shown in **Supplementary Fig. 26**. The sequence is represented by colors; unpaired nucleotides with blue, crossovers with orange, and the 14-nt seed dsDNA domain with green.

Staple ID	Length (bp)	Staple sequences
1	58	TAGTATTTAGCAACAATAACGAAAAGGAATTAATACCAAGCGCGCCACCGGAACCAAA
2	56	ATACATAAAGTTTTCAACATATAAGAAGCCCTTTTTTTAGAAAAGTAAGCTCAATT
3	56	ACCGACTTGATTTTTTTGGGAATTTGGCTTTTGATTTTTACAGGAGTGTATAAGTA
4	56	GATTGAGGGATTTTAGGTAAATATTAACAGTGCCTTTTATAAACAGTTAAGATTAG
5	53	GCTACAGCTTTGAAAGCGACAGAATCAAGTTTGCTTTTAGCGTCAGACTGACA
6	53	ATGCCACCTGCTCCTCATAGCCCCCTTATTAGCGTTTTCCATCTTTTCCCTCA
7	53	TAAAGGCTAATCTTTTACCATTAGCAAGGCCGGATTTTTCACCAATGACATTA
8	52	AGAACTTTTTTGATTAAGACGAAACGCAATTTTTAACGGAATACCCGCAAAT
9	51	AGCTTTTTTTTCGAGGGGCTTGATGGTTTAATTTCAATATCGGTGTAGCAT
10	51	CTAAATTTCTTTTTCTCATAAGAACCGAACTGACCAAAGGCTTTTCGCGTT
11	49	CTACTAATTTTAGTAGCATTACACCCTGACAACTTTCAACTGCCAGAGG
12	47	GGAGGTTTTAGGCAGGTCAGATGCTGTAGCTCAATTTTTTTTAAATA
13	47	GAGCCTTTTACCCTCAGAGCCTATAACAGTTGATTTTTAATTCTGCG
14	47	AAGCCTTTTATGGAAAGCGCAATTGCTCCTTTTGTTTTGAGGTCATT
15	46	TCAGGACGTTGATTGCGATTAGTAAATGAATTAGCGCTAGCAAGAA

16	46	ATTCAGTGAATATGACAAGCAAGCCCAATAGGGTTGATACTGGTAA
17	46	AAATCCGCGACTACGAAGATTATAGTCAGAAGCATTCCAGCCACCA
18	46	TCATACAAGAGCCAACCCAAATCAACGTACCGATACCACCACCCTC
19	46	TTCATCAAGAGCGCTTTTGAGTACCTTGAACCGCCACCCAGGGAGT
20	46	GAAAGATTCATTAGCGAGAAAATGTTTAGACTGGTGGCAAGATAGC
21	46	AAATCCTAACCATCCAGACCAGGCGCATCAGCGAAAAACTCCAACA
22	46	TTCTGAAGTTTACCATTTTAAGAACTGGGAAAATCAAAGTTTTGTC
23	45	CTCCTCAACAGCCCGGAGCCTTTAATTGCTTTAATACATTCAACC
24	45	CTGAAAAGGATAGCGATAAAAACCAAAACAGTTGATAGAAAATAC
25	45	TCGCTGAATTCATTGCAAAATCACCAGTCGTTCCATTTAGTACCG
26	45	CGGAACCCGCCACCTTTGACCATTAGATGTTCAGATCTTTGACCC
27	45	CGAGAGGAACCCATTAGTTGCGCCGACAAAGGCTTTATCACCGTC
28	44	TAAAGGAGGAAGAATTTATTTGTCACAATCAATTTTTAATTCA
29	43	AACGAGTAATAAATCAAGAATACACTAAAGGAGATTAAATCAC
30	43	TGCAACTAGATTAAGAGTTTCCATTAAACGAACGAGCGTTTTC
31	43	TTTGCGGACGGAAGCAGACAGCATCGGAACGGTGTAGATAGCA
32	42	GAACAACTTAGTTTCAGCGGAGTGAGAAAAAACGAGCAAAGA
33	42	GTTTCCAGACGATAATAATTTTTTTCACGTTCTCATTACCAG
34	42	ACCGGATGGCTTGCTCAGAACCGCCACCCAGGAGGGTAAGCG
35	42	CTACCCTGACTGCACCAACCTTTTAACGAAAATAAATTGTCG
36	42	GGAGGATTAGAGCGGGATCGTTTTCCCTCAGAGGCTGTGACC
37	42	ATTCAGGGATACAACCATCGCTTTACGCATAAACAAATGCTC
38	42	GAGAGATTTTTCACAAGAAATGAAAGTAACGATCTTCCAAAA
39	42	GGTCAATTTTTTGTTTAGCTATATTTTCAATCGTCAACCCTC
40	42	TAGCCCGTTTTAGGTGTATCACCGTACTCTCAGAGTATTCGG
41	42	GATTAGCTTTTTTTTGCTCAGTACCAGGAGTTTCGTCTTAAA
42	42	TCCACAGAGAGAAGTGCCCCCTGCCTATAAGGGCGCATTGTG
43	42	AAAAGGCTTATGCGAGCGCCAAAGACAATTCGGAAGCTGAGA
44	42	AATTACCTCCAAAATCATAGTTAGCGTATTAAGAGCCTATTA
45	42	AACACTGCGGATAAGTCAGTGCCTTGAGTGACGGAACGAGTA
46	42	ACAATGAGAATAAGAAATCTACGTTAATTAGAAAGTGGGATT
47	42	CACCACGAATAGCATCAGAGGGTAATTGTTCTGTAGAACAAC
48	42	TTGCTAAAACAAAGATAGCTATCTTACCAAGAAACACTAACG
49	42	CGAACAAGCAAACGGATTTAGGAATACCAGACGACGTCCAAT
50	42	GTTTACCACATTCATACGCAGTATGTTAAGTTACCGCGCGAG
51	42	ACTGCGGATTTGGGAGAAGGAAACCGAGTCCTTATACTAATG
52	42	TAAGTTTGGTGAATGCCCTGACGAGAAAATACCGAGTACCGT
53	42	TCAGAGCAGAGCCAAAACAAAGTACAACACACTCAAAACGAG
54	42	AATGACCGATTTAGCTCAGAACCGCCACATAATCATGTATCA
55	42	GAACCACTTTTCGGATGTTACTTAGCCGGGGTAAAGATTGCA
56	42	ATCGGCACACCAGAGTGTCTGGAAGTTTCAAAGCGATACGTA
57	42	TCAAAAAAAGTACGGCCGCCGCCAGCATTGTAGCGGCGCAGA
58	42	TTAATTCAATATAACGATTGGCCTTGATAATCAGTAGAGGAC
59	42	GCACCGTATTCACAGAGCTTAATTGCTGGAGCTTCAGCAACG
60	42	AGATGAACGAGGGTAAAGCGAACCAGACTGGCTTAAACAAAT
61	42	CAGCTTGCACCAGAAATTATTCATTAAATAACGGGGTGCCGT
62	41	CAGATACCTATCATATAAATATTCATTATCCCCCTCAGGCA

63	41	CGGTCAAATGAGGAAGGAAGCCCGAAACTTCAAATAGAGGA
64	41	GTAAATTGTGAATTTCACCAGTACAAAACAACGCCTTTATC
65	39	GTAATAGTAGGCTTTTGCTTTAAGAAGTATTATTAGGTA
66	39	CCAGCGATTCGAAATGCTTTAAACAACATTTCGCCTCCC
67	39	CCACCCTCATAGTCTCTGAATTTACAGCACCAGACAAGA
68	36	TATGACTTGAGTTATTTTCCAATAATAAGAATATCA
69	25	TCGCCTGGAGGCAAAAAATCAGGT

Supplementary Table 19 Staple sequence for the 6HB-based star DNA origami of 42-bp edge-length. The sequence is represented by colors; unpaired nucleotides with blue, crossovers with orange, and the 14-nt seed dsDNA domain with green.

Staple ID	Length (bp)	Staple sequences
1	58	CATACAGCGTTTGCCGCCACCATCAGAGCCACCACCCGGAACCGCATTTTCGTTATTC
2	58	CTAATGCAGCTATCAAAAATCAGGTGCGACCTATATCTGGTCAGTTAGGCGGTAAAAG
3	58	GAAGGTTTTAAATATTGACGGAAAGTCATAATCAAGGGATAGCAAGTTATAGGAACCC
4	57	CAAAGTTTAAGAAACAAAAGAAGTTTTGCCAGAGGGTTTTTTGGTAATAGTACTTTA
5	57	GGAATTAGGCGACAGAGTTTCGTTAATCTTGACAAGAATTTTTTTCCGGATATTCAT
6	56	AACACCTCAAGAAGATTTTGCTAAACAGAGGTTTAGTTTTTTTACCGCCACCCTC
7	56	TGACCTCAACTCGATGCCACTACGAAGAACAGGTCAGTTTTTTTGATTAGAGAGTA
8	56	AGGCATTTTTGTCAGACGATTGATACAGTAATTACTAGAAAAAAGAAGAACAGTTT
9	56	AATCATTTTTGTTTTTTGGGGAGCGAAAGGCATTTTCGAGCCGGTTTAACGAGGTG
10	56	CTCAATTTTTCTATCGGCCTTAACCAATTGAACAAGAAAAATATTATCAACCCTCA
11	56	CGCCATTTGCCATTGCAACAGGAATTCGAACCTCCTAATTGCTGAATTATGCTGTA
12	56	ATTATTTTTTTATCCCAATCCCTGAACATAAGAATACGTGGCTAGACTTCCTAAAA
13	56	GAACCTTTCTAAAGGGAGCCCCCGAGTTTAGACAGTAGATACATTTTTAATGGTCA
14	56	AGCGCTTTTTAAAGACAAAAGGAGCCAGGCAAGACAAAGAACTATTAATCCAGTAC
15	56	TAATTTACAGTAAATCGGTTTATCAGCCGCGAGCTGATTTTTTTAAAGGTGGCATC
16	56	GTCAATTTAAATGAAAATAGCAGCATAAACGCAATAAATATCGCGTTTATTCGAGC
17	56	GAGGTGGGCAAATTTGTGTCGAAATCCCTTTACCCTGTTTTTTTACTATTATAGTC
18	56	AATAGAATCAATAGGCCGCTTTTGCGGACGGTGTCTGTTTTTTTGAAGTTTCATTC
19	55	CACCTTTTTGCTGAACCTACGAGGCGCAGTTTCAATCCAGTGATTGGCTTGCCCT
20	55	ATTAAACCCCCTGCCTATTTCTCAGAGTCACCGGAACCAGAATCTTTTCATAGCC
21	55	AACCGTTTTTATCAGGGCGATAACCACTACCAGTATAAAGCAATCGCGATAATAA
22	54	CTACCTAGGCCGGAAACGTCTCAATAGCAAAGCTGCTCATTATAAGGGAAGCAT
23	54	TATGCGTTATTAATTCTCACACCTGCGCTTCTGAATTTCGTTCCAGTAAATAAA
24	54	AATGGCTATTTCTTTAAAGACGATAAAAACAATAACTTAAACAGGGAAGTTAAC
25	54	AAAGTACCGTTAAGGTACCGCACTCGAGAAACGGGGTTCCGGCGAACGTAATCG
26	54	AAATATATTTTTTAATTGTATAATTTAATGGGTGAATTCACCGTCACCGGGAGG
27	54	GTTCAGAGAATCCCCAGTGCCACGCTGAGAGCTTAGCAAAAAACCATTTAGCAG
28	53	TCCTCTTTATTAAAGCCAGAATGGTTGATAAGTGCCGTCGATAAATCGCGAAA
29	52	TGAAATGTTATTTACAAGAATTTTAAGCAAATACTTATTTTGACGCTATTAC

30	52	AGAATCAAGAAACGAACGAGTAGATGTTAGCAAACGTATTTTTTTGAAAATA
31	51	CACCGAATAAGTTTATTTTTTTGTCACAAACCAATGTGAAAAATCGAGAGA
32	51	GCGGGGTCTCCTCATACTATGGTTGCTTTGACGAGCTTTTTTTACGTATAA
33	51	ATTCTAAATCAGATAATGAAATAGCAATAGCTATCTTTTTTTTTACCGAAG
34	51	TAAACAGTCCTCGTCATCGTAGGAATCATTACCGCGTTTTTTTCCCAATAG
35	51	ACAAACAGGAATCAGAGTGTACTGTTTTTTTGTAATAAGTTTTAACGGTTG
36	51	AATTCGAGAAAGCGCCCTGAACAATTTTTTTAGTCAGAGGGTAATTGCCAT
37	51	CAAGATTATTTTTTTGTTGCTATTTTGCCAGAAGGAAAAAGAACCTACGTT
38	51	GCAATTCTAAGTCCCAATAATCGGTTTTTTTCTGTCTTTCCTTATCAAGAA
39	51	CCCTTAGTCCAATCCAAAATCACCTTTTTTTAGTAGCACCATTACCTTACC
40	51	TTTATAATTTTTTTTCAGTGAGGCCACCACGCGAGGCTTGAAGCTCCTGAA
41	51	TGAATATAGGCAGAGGAGCGGGCGTTTTTTTCTAGGGCGCTGGCAAACCCA
42	51	GTTGTTCCTTTTTTTAGTTTGGAACAAGGGCCGATTATCCTGAGCATCACG
43	51	CGCCTCCCTTTTTTTTCAGAGCCGCCACTGCTCAGTAAGATAGGCGTGGAC
44	50	GCGGAATCGTTTAAATATTCATTAATACCTTATTTTGCGATTTTAATTTC
45	50	AGATAATTTTACATTTGAGAATACACTAATTCTCATCAGAGGATCGAAAG
46	50	GAAGGATTTGCGGAATTACATCGGAACGATTAGCAACTTTTGCTGGCTTA
47	50	CAGAAATTTTAAAGAAATCTTGATACCGATTTGCGCCGAGTAGTAGTTTG
48	50	TGAATATTTACCTTGCTTGTAGCATTCCATTCAGCCCGAGCCATCCTCAT
49	49	ACGGAGATTTGTATTGATAAACAACAGTTGAAAGTTGGAAGGTTATCTA
50	49	AGTTTCCATTAAATATACGTATATTAAATCCTTTTTCGTTATTAATTTT
51	49	CGGTCGCTGAGGCTAGTTAAATAATCCTGATTGTTTATACTTCTGAATA
52	49	AAAGGCTCCAAAATTAATTGTCAGTACCTTTTACTTGAAACAATAACGG
53	49	GACGTTAGTAAATTTGTATGGAACAAAATTAATTTTACAATTTCATTTG
54	49	AACGGTGTACAGATATAGGCTGAAAACATAGCGATTGATTAAGACGCTG
55	49	GAATTTTTTTTTACGAGGCATAGTAAGTAGGAATACCTTTTTCATTCAA
56	49	GCTCAACATGAAGACAGTCATCATATTCCTGAATATCCCATCCATCGTC
57	48	GCGTCTTTTTTTAGACTGTAGCGCGTTTATAAAGGTGTTTTTCAACAT
58	48	ATAAACAGAGCGCTACGACCAGTAATAAAACATTATTTTTCATGAGGA
59	48	GACAGGAGGGGTCAATGGTTTGAAATACTAATGGATGTCGTCTTTCCA
60	48	TGAGTAGATTCCAAGACAATAAACAACAGAACCTATAACCGATATATT
61	48	TGAGATTAGCAACAAACATCGCCATTAAGAGCACTGAAACAAAGTACA
62	47	ACCATGATAGCAATACTTCTTTTTGATTAGTAACCAAGTAAAGTAAT
63	47	TTTCAAACCGCCACCAGAATTTCACCACCAGAGAGTGCCCTCATCTT
64	47	ACTTCAAACGGAACAACATTTTATTACAGGTAGGTTTACCTGCGCGA
65	47	GAGCTCGACGAGCGTCTTTTTTCAGAGCCTAATAACCCACATTGGCA
66	45	AGAAGCAAAGCAAGCGCAACAACTAATAGATGCCCTAACTATCAT
67	45	CCTTTAATTGTAAAGACTCATTTTGCGGAACCAGTCACAATATCA
68	45	TTCAAAGCGAGGCAAAAGGATTTAGAAGTATACAGACAGACGGGA
69	45	CATATAACAGCCACGCACCATATCAAAATTAAGACGACGAACGGG
70	45	AATTCTACTAACGTTGAATACCAAGTTACAACAACGCTGGTCACG
71	45	ATAACCTGTTTAAACAGTGCGTAGATTTTCAAGTAATAAAGGAAG
72	45	AGAACCGCCAAAAGTTTAACAGTACATAAATAAATTTAGTGCCTT
73	45	AGGAACTTAAAGGAATCGGCTTCCCTTAGAGGGTTTATAAGTATA
74	45	AGCATGTAGAGCTGGTATATGCAACTAAAGTGATCGTCGATGATG
75	45	TACCCAAATCACTTTGATTATCAAAATCATAGGTCTTAAACCGAA
76	44	CTTGAGAACACCAGCAAAGACACCACGGTAATCAGTCCAATACT
77	44	AAACTACTAACACTTTCAACCGATTGAGACTTGAGAACTTTTTC
78	44	CAGCGGATAGGTGTATATTCACAAACAAGCGTCATTTAGTATCA

79	44	CGAAAGAACCAGACAAACGATTTTTGTCGCATTAATATTTTTG
80	44	AATTTCTTAGCTATGCCGTAAAGCACTAGGCGAGAAGAGAATAT
81	43	GCCCGGAAGTGAGAAAATTACCTGAGCAAAGCCTGTACATGGC
82	43	GACGAGAATGGTTTAAGAACTGGCTCATTGATAGCGTAGCGAC
83	43	ATGTACCGAACGCCTCTGTAAATCGTCGCGCGAGAACCATTTG
84	42	CAAACCCCCTGCAACCTCAAATGATACATAACGCCCAGTATT
85	42	CGCTACATCAAAGGAAAAGAATAGCCCGCCAGGCGAGGATTA
86	42	AATAAAAGCTTTTGAGTAAGCAGCTTTACAGAGAGCAAAATA
87	42	TCTTACCAAGAAACATAGAAGGCAAACGCTCATGGCCAATAA
88	42	CAAATTATTATTTTTAGAATCAGATTTAGAGCTTGCAAGCAA
89	42	TCCAACGGGGCGCGAGAGAAGGAAAAGCGCAGTCTAATGCGC
90	42	CTGACCTCAATATATAGCGTAACGATCTCCCTCAGCAGCATT
91	42	ACTGATATAGAGCCCCCAGCGATTATACCGGATTGCATCAGT
92	42	AACACCGTCAATCAGCTCCATGTTACTTACCATAATTAAACA
93	42	GATTCACAAAGAAAGAGGCTTTGAGGACCTCCTTTTTACAAA
94	42	GAATTAAAAATAAGCGGAAGCAAACTCCGCACCAATACAAAC
95	42	GCGAGAGCGAACTATAACGGAATACCCAACCGAGGAGCCGAA
96	42	TTTTGATGGCCTTGATCACCGTACTCAGACTTTCATGATGAA
97	42	TCTGTCCTTTGCACACAACAACCATCGCTTGATTCACTTGCC
98	42	TAAGAGCAACGCTAACTTGCGGGAGGTTGTTTTAGATCCGGT
99	42	CTGACCAAACGTAAAAAATTCATATGGTATTAGCATTTTAAC
100	42	TTTTTTCATAGTAGCTACGTGAACCATCGTGTAGCCAACAGT
101	42	GGAAGAATCGAGGTATTTTCATTTGGGGTTGCTTTCGTCAGA
102	42	GCCGTTTACCGTTGACGGTACGCCAGAAAAGGGATCGGGAGC
103	41	TGCAAAAATCCTTGGCTGACCTTCATCAAGAGCATAATTTT
104	41	AACTTTTTTATTGTGAATACGAGAATGAGCCGGACAAATAT
105	41	AACCCTCAAAGATTCATCAAAAAGATTATTTGACCGTCAAT
106	41	GAGAGATTTGCCAGTGATAAGAGGTCATGGCTACACCACCA
107	41	TATTAAATAACATCCCAATTCTGCGAACGACAATGGTAAAA
108	41	CTGCGCGTGGCCCATAGCATTTTCCGAAATTGCGACAGAGG
109	41	GAGTAACCCGCCGCAACCGCCACCCTCATCATAGTTGTGAG
110	40	AAATATCTTTAGAAATACCGAACGAACCACCTATAAAACA
111	40	AAAAGTTTGAGTAAGGGACATTCTGGCCAACTAACCCTTC
112	40	ATGGAAGGGTTATGTTCAGCTAATGCAGAACTTTATCAAC
113	40	ATTCGCCTGATTAATTGAGAATCGCCATATTTCCAACATG
114	40	AATTACCTTTTTCGACCGTGTGATAAATAAGTTAAGAATA
115	40	AGAAGAGTCAATTTAGGTTGGGTTATATAACTAATGCTGA
116	40	ATAAAAGTTTGCAAATGTTTAGACTGATACCAGAATTGGG
117	39	CCCTTTTACACCCAGCTTTTTTTACAATTTTACTTAAAT
118	39	GCAGCGATTTTAAAATATCCAGAACAATCATAATTTACG
119	39	CAAGCAAGAGAGTAAAATTTTTTGAGTCTGTCAAGTGTT
120	39	CGTGCTTGAAGTCCACTTTTTTTATTAAAGAAGTTGAGT
121	39	GCTGAGATTCCTCAGAATTTTTTCCGCCACCCCCGGAAC
122	36	TTAAGACTTTTTTTCCTTATTACGCAGTTATCAGGA
123	30	CGTTGGTTTTTTTAAGAAAAATTGGCATGA
124	27	AGGGCTTGCTTTGAAAATCTCCAAAAA
125	27	CTCCGGCAGTGAATAAGAGGACAGATG
126	26	AACATGAAAGTTTTTTTTATTAAGAG
127	25	CCCTTATTTCATCGTATTATTCTGA

128	23	CGAATTTTTATTCATTTCTAGAA

Supplementary Table 20 Staple sequence for the 6HB-based curved beam DNA origami of 42-bp edge-length. The sequence is represented by colors; unpaired nucleotides with blue, and crossovers with orange, the 14-nt seed dsDNA domain with green, and the 4-nt dsDNA domain with red.

Staple ID	Length (bp)	Staple sequences
1	60	CTAACTCACGGTAAAACGAACTAACATACCAGAGCTGCATTAATGAAGCACTGCCCGCTT
2	59	ATGAATTTTTTGTATGGGATTTTGCAGAACCGCCATTTTCTCAGAGCCACCGTTAGTAA
3	59	AGGGAGCCCCTTTTTTTAGAGCCACCAACCTAATTTAAAGAGGCAAAAGAATAAAATAT
4	58	GTTTTCCCAGTTTTTCGACGTTGTAAAAGGGATGTGCTTTTTTTGCAAGGCGCACACG
5	58	AATCAGTTATCAAAATCATATTCTGAGAGACTACCAATGGAAACTTTTTCATAAATCA
6	58	GATTATCGAGCCGGAAGCATTTGTGTAAAGCCTGGCAGGAAGATTTTTTCTCCAGCCA
7	58	GGCTACAATAAGAGGGAACCGTTTTCTCAGAGCCGCCCTTTTCATATTTTTCAAAATC
8	58	AATCGCAACGTTATTAATTTTTAAGTTTGAGTAACGGAATTGAGTTTTTGTTATCTAA
9	57	CAGGAAAAACTGAAAGCGTAAGATACGTGGCACAGACAATATTTAATATCACTTTCA
10	56	ACGTTACCAGCGCCGAGCACCTGAGGCTTGCAGGAAGTTTTCACAATCAAAATAGC
11	55	GTTGCGCTTATCGGCCTGGTGCCTCATTATTACAGGTAGATTTTAAACGCGCGGG
12	55	AGTCAATGAGTGAAATATGCGTTATACAAATTCTTTAACCAATTGAACGCCATCA
13	55	CGCGAGGCGTTTTTCGAACCTCCCGATTACGCCAACTTTTATGTAATTTCTTAAT
14	55	TCATTCCGTTTATCAGCCTAATTTGCCAGTTACGAAGATTGTTTAGCAAATATTT
15	55	GATTGTTTGGTTTTACTTCTGAATAATTTGAGAGCCTTTTAGCAGCAAATTAACA
16	55	TTCCACAGCACCGCAATACCGAACGAACCACCACCGTGCATCTTCAGTTTGAGGG
17	55	AAATCCTTAGGAGCCAGTACCTTTTACATCGGGACAACCCGTTTATTCTCCGTGG
18	55	TATCTTAGTGTACTGGTAATGAGTTAATATGGTTGCTTTGACAAAGACGAAAATT
19	55	ATATATTTTATTTTTTTCATCTTCTGGTCGAATTATTTTTTCATTTCAAATACCA
20	54	AAGAGTGCAAAAGAAGTTTTTGCCAGAGGGGGTAATAGTAGACCAGGTCCCTTA
21	54	CCCTGAGGCATAGTAAGAGTCAACACTATCATAACCCTCGACAAAGCTGCAGCA
22	54	TTATGCGAAAGATTCATCATGTTGAGATTTAGGAATACCATTAATTTGCCAGGG
23	54	CGCGTTTTTTTTTCGAGCTTCAAAGCACGAAGGTTGACGGGGAAAGCTTTAGTT
24	54	TCGCCTTCTTTACCCTGACTTATTATAGTCAGAAGCAAAGGCGAAACAATCAAG
25	54	GGGAACTAAATATTCATTGTAATCCCCCTCAAATGCTTTAGCCGGAAAGAACGT
26	54	AAGGTGAATAATTCTACTTTAGTAGTAGCATTGTAAAACTGTCTATCTGTGTCG
27	52	GTCAGACGATTTTTTCCTTGATATTCACAAGGAAACCGAGGAATTCAATAAT
28	52	CGCAGTATGTTTTTTCAAACGTAGAAAATGGTATTAAACCAAGTTCGCACTC
29	51	ACACTGATTTTTTTGTTTCGTCACCAGTAAATCTACGTTAATAACGCCAGG
30	51	AACGGATTGAGCGCTTTTTTTCAGAGAGATAACCCACTACCAGAAACAAAT
31	51	GGATAAAGTATTTTTTTTACAAACAATTGATTAGAGCTTTTTTCAATAGAT
32	51	CCACCACGCGTTTTCTTTTTGCATTTTCGGTCATAGCAGAGCCAACCGGAA
33	51	AGAGGGAAAACATTTTATAGCTTAGATTTAAATCGTCTTTTTTATTAATTA
34	51	ACAGGACCGAAGTTTTTTTAAGAAAAGTAATAAGAGCTTTTTGAAACAATG

35	51	ATCGAGCAACATGTTTTTTTTAATGCAGAACGCGCCTAAGAACGACATACA
36	51	TAAACCATGTAGTTTTAATCAATAATCGCCTGAACAATTTTTAAAATAATA
37	50	AGTTTTTCAAAAATGTTTTAAAATAGCAAATAAGAAACGATTTTTGTTTA
38	50	CCGTTATCATTACCATTTTTTAGCAAGGTAGAGCCAGCAATTTTCCAGTA
39	49	AGAGCTAGTAAGCGTGCAGCGAACACCCGCCGCGCTTAATTTGCCGCTA
40	49	CAGGGCAACCGATTTTGGAGGGAAGGTAAATATTGTGGGAATCCGGAAA
41	48	CCCACTACGTTGAACCATCACCCAAAAGTACAACGGAGTTTTGTATCA
42	48	TTGGAACAAGTAGTCCACTATTAACGAGGCGCAGACGGTTAATCATAA
43	48	TGGTGGTTCCTGAAATCGGCAAAACGCATAGGCTGGCTTTCCTTCATC
44	48	TCACCGCCTGTGCCCTGAGAGAGTTGCTCATTCAGTGATTAAGGCTTG
45	48	GAGAGGCGGTTTGCGTATTGGGCCAACTTTAATCATTTTGAATTACC
46	47	AGTCTTTTCAAACTGTGAGAATAGAAAGGAACTCTAAAGGAAGAATA
47	47	GACGACGACATCGGCCAGAACTGGCTCATTGGAACAAAATGAGTGAG
48	47	GTGTACAAAATGTTTAGGAATCATTACCGCGCCCTTTTTAGCAAGCA
49	47	CAACGTATTTACCATATAACTATATGTAAATGCTTTTTTGCAAATCC
50	47	AGATGGTCATTCAACCAGAAGGAGCGGAATTATCTTTTTATATTCCT
51	46	TTCTGGCCAACTTGACGCGCGTAACGATCTAAAAGCCCACAGTGCC
52	46	AGGAGCGGGTTTTGGGCGCTGGCAAGTGGAAGTTTTTTCCATATAA
53	45	TCCAGACACCCTCAGCAGGTCGACTCTTAGGATCCCCGGGGGAAG
54	45	CGAAAGGCGACGGCATAGGAACCCATGTATAGTTATCAATCGTCT
55	45	CAATAAAAACAAGCCTGCGGAATCGTCACGAACTGTTGTTCCAGT
56	45	TATATTTGGGTCGACGATTATACCAAGCCGGATTGGTTGAGGCAG
57	45	AGGGTAAATACCCAAATCAAAAATCAGGGATAAATAGGGCGATGG
58	45	CAGTTGATTCAAGGGAAGTTTCCATTAAACGCCAACAGGAACCGC
59	45	ATGTACCAACGTCACTCCATGTTACTTAAACAGTTCTCCTTATTA
60	45	ACTGTAGCCAGAGCGCCCGAAAGACTTCCACTAAAGGAACCCTAA
61	45	TGCTTTCCTTTTTAGAATCAGAGCATCCTGATTTTACCAACGCTA
62	45	CGTCACCCATTAAATAGCCGAACAAAGTAAGAATTTTATTCATTA
63	45	CTTTTTTTTTTTAAAGCGAGAGGCTTTTAATCTTGTCCTGTTTGA
64	45	ACAGGAACGTTTCGCCAGAATCCTCCGGAATTTTTTTACTAGAAA
65	45	GTTGAAAATTATCAAAAAGGAATTACGACGAGAAATGATTGCCCT
66	45	AAAGAGTCTTTTCATCACGCAAATTAGATTTTTTTTTTAACGTCA
67	45	CATCACTTGTTTGAGTAGAAGAACAATGCGCTTTTGATAGCCCTA
68	44	AAGACTTTTTCAGTACCTTTAATTTTTCTTTTGGAGGCTTTGTA
69	44	AATTGACCAGGCAATTTTTCGCCATTCGCCCATGGTCAGGTTTA
70	44	TGAGGCGCATTAAATTCTGGTGCCGGAATTATCCGTATAATCCT
71	44	ACTGTTGTACCGAGAGAACCGCCACCCTCTAAACACAGAACAAT
72	44	CCTGATTACAGTAAACTAACAACTAATACGACAACCTTTTTCAA
73	44	AAATTGTAATTGAGTGACCAACTTTGAAAGTCCAATAAAGCCGT
74	44	ACGAGCGTCCAATCCAGCCTTTACAGAGAGGGCAACATTCCTTA
75	44	GATAAGTGCTGTCTTATAAAAGAAACGCAAAGATTTTCGGAATA
76	44	AAAATAATTGCGAAAAACAAGAACCGGATACCAAAATCCTCCGG
77	44	AAGCCTGTTAGTAGGGAGGCAGAGGCATTTTCCGGTATGAGAAG
78	44	GCTCAACTAGTATCTAACCTTGCTTCTGAAGACGCTTCTAAGAA
79	44	GAACAAACGCAACAGCCACCAGAACGAGTATAACGCCTTTTGCG
80	44	GATGAATATGCTTTGATTACCTGAGCAAAAGAGAAAATCGTATT
81	44	GCCCAATAAGCAGAGCCAGAATGGAAAGCGCAGTTTTGAATTTA
82	44	AAACATCGCGTCAGTATGAAAAATCTAAAGTCATCAACTCACAA
83	44	TTGCCATACCCTCAGTCAGGATTAGAGATGAGGAAGAAAGCGAA

84	43	CATATGGATTTTGTAACGGGGTCAGTGCTCGGTCGGTATAACG
85	43	AAAATTATGGAAGGATAAGTATAGCCCGTTGCGAAAGTAATAA
86	43	AATATCTTTTGCCCGAAGACAAAGAACGCGAAGATGGGATTCG
87	43	TGACCATTTATACATTTCGCAAATGGTCACTAAATCACACTCA
88	43	CGACCGTACCTAAAATTAGGATTAGCGGCCTTTAAACCGAGTA
89	43	TGAGAATTTTATATTTAACAAGTTGCTATTTAAAGGTAGTTGC
90	43	AGTTACTTTTCGCGCAGAGGGATAAATAATGAGGCCTTGTATC
91	43	CCGCCTTTTTAGTGCCACGCTGCACGTAATTTGATTTAATAAT
92	43	ATCAAGACTTGCGGTCGGAACCTATTATATACCGAGATTTTAG
93	43	ATATATGTAGTGAATATATAGAAGGCTTATCGAGCCAGCCAAC
94	43	GCTTTCCGCAACATACAGATGATGGCAATCATCACCACAGAGG
95	42	ACAGTTTCACCCTCCTCGAATTCGTAATATTCAGGGGCTATT
96	42	GCCGACAGCCTATTGAGGTTTTGCTAATTTACGAGAGTTAAT
97	42	TCGAGAGCGTTGAATGTAGCAATACTTCAACAGAACCATATC
98	42	GGTTTATGAGAAGGTTTAATGGTCTTAGAATCCTTCTGAGAC
99	42	TTTTTCAGGTTGATGTTAGAACCTTGAGGATTTAGAGTGCCG
100	42	GTACCGCCAGCGGAATCGGCCTTGCTGGTTTGAATCTGCGCA
101	42	TGGGAAGAAACAGATTCACCAACCTGTCGTGCCTCAGGACGT
102	42	GAGCTGATTTTTGGTGGCATCTATCACCGTCACCCATCGATA
103	42	AGAAAAGTTTTTCAAAAACAGAAAATAAACAGCTAAAAACAG
104	42	TTGTTAATTTTTAGCTCATTTTTACCAGTATAAAGTAATAAG
105	42	CATTAAATTTTTGAGCGAGTAAGAAACAATAACATGAAACAA
106	42	GTAGATGTTTTTGCATCGTAAGCAGAAGATAAATTGCTGAAC
107	42	AAGCTTGATTACGCGAACCCTTCTGACCGCTCATGTCGTCTT
108	42	TTTTTTGTCATTTGACAGAATCAAGTTTACAGGAGCATCAAA
109	42	TCTTTGAAGAGGAACGCCGCCAGCATTGGCCTTTAGTTTAGC
110	42	AAGATTACCCCCAGGGTGCCGTAAAGCAATAACCTGCGTCAG
111	42	CACCCTCCCCCTTATGCGAACGAGTAGACGGCGAATACGTAA
112	42	GCAAACTGGTAAAACGTGGCGAGAAAGGCCAATTCTTAGCGT
113	42	TTGCGGAACGAGGGGTCACGCTGCGCGTATGCAACTGCTGTA
114	42	GCTCAACCCTAATTGCTGAATATAGAGCCACCACCGTCATTT
115	42	GGATAGCAGTTTTGGAAATACCTACATTAGAGATACAGCTGG
116	42	GGACTCCCCGGTTGATTAACTGAACACCATTAAGACAGAAAA
117	42	AAATCCGGACCATAAAAGAACTGGCATGCTGAACACAATCAT
118	42	CGAGAATCGACCTGAAGGGCGAAAAACCAGCATGTAAGTCAG
119	42	AAATCCTAATGAAACGACTTGAGCCATTACGGAAAGAGTTAA
120	42	TAAATCATCGCATTAGGTAAAGTAATTCTTCATCGTAGACTG
121	42	TTTTATTTGTCCAGTATTTTGTTAAAATAAAGAATATGAACG
122	42	GATAGCGAGGACAGAGCCCGAGATAGGGACGTTAAACGACGA
123	42	TAAAGGTAATAACACATATTATTTATCCTTTCCAGAACAATA
124	42	GCCCCCTATGACAAAAACAGGAGGCCGATTTGCACGCCTTAA
125	42	AGCGGTCCAGCTTTTTACATTTAACAATTGGGTTAGACGACG
126	42	CTTAGGTTTCATTTGGCCTTCCTGTAGCCACGCTGCCCAAAT
127	42	ATAAAAATTCATTAGTTTGCCCCAGCAGCGCGTCTGAATTAC
128	42	TCCTCAACAGCTTGGTTTTTATAATCAGGGCGTTAGAAATAC
129	42	TGGTTTTAGGTCACCAATATCTGGTCAGGAAACCACTAATGC
130	42	GAACAAATTGGCAAGACCGTAATGGGATTCTTTTCGGGCTTG
131	42	AGATACAGTAAATTACCAGTGAGACGGGGCGGATTATCAACA
132	41	GGCGATTTTTGTGCGGGCCTCTTCGCTCATGCCTTTTTCAG

133	40	GCACGTTTTAAATAACCACCAAGACAGCATCGGATGGCTT
134	39	TTGCGGGATTTCACCCTCACATACATGGCTTTTGATGAT
135	39	CCCACGCATTCCGATATATCTTGAGTAACTTTGCCCGTA
136	39	TGAATTTCTTAACAGCTTGTCTGAAACATTTTAGTATTA
137	39	AAAAAAGGCTCAAAAGGAGGTTTTGCTCTTTACCAGGC
138	39	ACCGGAACCAATAAAGTACGGTGTCTGTAGCGTAGCAAC
139	39	AAATAGCTAAAAAGGGCGACATTCGCGTACAGGCCGCTT
140	39	TCCCATCAACCAGCTACAATTTTGGGAGCTCAACCATCG
141	39	ATTTTCCTTAATAAGAATAAACAGAGAAGTCTTTCGAGG
142	39	AATACATTAATAAAGAAATTGCGTAACCGTAATCTCCAA
143	38	GGTGTTTTACCGTACTCAGGATAGCTGTTTTTGTGTGA
144	38	CGCCTGTAGTTTTTTTTCCACAGACAGCCCTCACCGTA
145	38	GAAATGGATTTTTTTTTATTTACATTGGCAAACTACAA
146	37	TGCCACTGAACCAGCCACCCTCAGAGCCTTACCAGAA
147	32	TCCAGTCGGGAAGTATTAAGTTGATTAATTGC
148	27	GCAGCACCTTTTTCAGTAGCGGGGCGC
149	27	GGAAGCGCTTTTGACGGGAGAATAATC
150	27	AGAATATATTTTACCGACAAAAAATTT
151	27	ACATCAAGTTTTCAAAATTAACATCAA
152	27	CTCAAATATTTTACCCTCAATGTTGGT
153	22	ATTACCGCCTTTTCCATTGCAA
154	21	ACCAGTATTTTTAAAGGGACA
